# Dwarf Mistletoes (*Arceuthobium*, Viscaceae) of North America: Classification Systems, Phylogenetic Relationships, and Taxonomic Characteristics

**DOI:** 10.3390/plants14132051

**Published:** 2025-07-04

**Authors:** Shawn C. Kenaley, Robert L. Mathiasen

**Affiliations:** 1 Department of Environmental Conservation and Horticulture, Finger Lakes Community College, Canandaigua, NY 14424, USA; 2 School of Forestry, Northern Arizona University, Flagstaff, AZ 86011, USA; robert.mathiasen@nau.edu

**Keywords:** *Arceuthobium*, geographic distributions, host specialization, morphology, parasitic plants, species, subspecies

## Abstract

*Arceuthobium*—the dwarf mistletoes—is a clearly defined genus of hemi-parasitic plants in the family Viscaceae. The genus occurs throughout much of the Northern Hemisphere; however, the greatest concentration of species and subspecies occurs within coniferous forests of western North America, where considerable research was executed in the mid-to-late 20th century to determine their geographic distributions, host specializations, and taxonomic boundaries. However, the last monograph of *Arceuthobium* presenting morphological, phenological, phylogenetic, and physiological information for N. American dwarf mistletoes was published in 1996, and since that time, no subsequent publications have presented taxonomic information for the present classification of N. American *Arceuthobium*. Thus, herein, we provide updated phylogenetic and taxonomic data for 44 taxa of *Arceuthobium* indigenous to N. America while simultaneously addressing knowledge gaps and suggesting future research to improve our understanding of these ecologically and economically important forest tree parasites. The present classification systems for and recent treatments of N. American *Arceuthobium* are also discussed.

## 1. Introduction

*Arceuthobium* M. Bieb (Viscaceae)—the dwarf mistletoes—is a well-characterized genus of angiospermous, obligate hemi-parasitic plants that infect conifers within the family Pinaceae or Cupressaceae across the Northern Hemisphere [1,2,3,4]. Given their complete dependence on host trees for water and mineral nutrition, as well as their near complete dependence on host trees for carbohydrates [5,6,7,8,9,10,11], the ecological and economical importance of dwarf mistletoes in managed and unmanaged forests has long been recognized [1,12,13,14,15,16,17,18,19,20,21,22,23,24,25,26]. Severe infection of individual trees can significantly alter host anatomy and physiology (e.g., hypertrophy, increased respiration, and the stimulation of witches’ brooms), reduce host growth, fecundity, and longevity, and increase host susceptibility to drought, fire, insects, and pathogens [1,25,27,28,29,30]. Likewise, scaling up the negative tree-level effects of dwarf mistletoes to forest stands and, thereafter, to forest ecosystems, dwarf mistletoes affect forest composition and structure by persistently influencing tree mortality and host recruitment while simultaneously altering forest susceptibility to catastrophic wildfires, insect attacks, and disease outbreaks [1,25]. Thus, where they occur in the New and Old World, dwarf mistletoes are serious pathogens of economic concern, affecting forest health and forest productivity. The latter is particularly true in North America, where millions of hectares of coniferous forests are dwarf mistletoe-infested throughout western Canada, the western United States, and Mexico; resulting in an approximate annual loss of >17 million cubic meters of wood and a combined loss of billions in USD annually [18,19,21,24]. Although a demonstrated threat to timber quality and yield in commercial forests, dwarf mistletoes are also important ecologically as biological disturbance agents, and their interactions, directly and/or indirectly, with diverse assemblages of forest-borne animals, flora, and microbes significantly influence short- and long-term forest dynamics, as well as organismal biodiversity [1,25,31,32,33,34].

Although present in the New and Old World, the species diversity of *Arceuthobium* is greater in N. American coniferous forests when compared to Europe, Africa, and Asia combined [1]. A total of eight species occur outside of N. America, whereas 44 taxa—constituting 30 species and 14 subspecies—are recognized to represent the genus across N. America (Table 1) [1,17,25,35,36,37,38,39,40]. Two areas of *Arceuthobium* diversity exist in N. America: (1) northern California and southern Oregon in the USA (n = 17 taxa); and (2) Durango, Mexico (n = 11 taxa; Table 1) [1,39,41]. Examining taxa by country reveals that six taxa occur in Canada (*A. americanum* Nutt. ex Engelm., *A. douglasii* Engelm., *A. laricis* (Piper) St. John, *A. pusillum* Peck, *A. tsugense* (Rosendahl) G. N. Jones subsp. *contortae* Wass & Mathiasen and *A. tsugense* (Rosendahl) G. N. Jones subsp. *tsugense*), and the geographic distributions of these taxa also extend into the USA, while 17 taxa are indigenous only to the western USA (Table 1) [1,42,43]. Likewise, the geographic distribution of eight taxa (*A. abietinum* (Engelm.) Engelm. ex Munz subsp. *mathiasenii* Kenaley, *A. apachecum* Hawksw. & Wiens; *A. blumeri* A. Nelson, *A. campylopodum* Engelm., *A. divaricatum* Engelm., *A. douglasii*, *A. gillii* Hawksw. & Wiens, *A. vaginatum* (Willd.) Presl. subsp. *cryptopodum* (Engelm.) Hawksw. & Wiens) occurs in the USA and extends into Mexico [1,44]. The biogeography and species diversity of *Arceuthobium* in Mexico and Central America remain understudied [1,17,35,41,45,46,47,48,49,50,51,52,53,54]. However, 12 taxa presently are unique to Mexico (Table 1), whereas three taxa are present in Honduras (*A. globosum* Hawksw. & Wiens subsp. *grandicaule* Hawksw. & Wiens, as well as *A. hondurense* Hawksw. & Wiens subsp. *hondurense* and subsp. *hawksworthii* (Wiens & C. G. Shaw bis) Mathiasen) [1,36,53], and one taxon occurs in Belize (*A. hondurense* subsp. *hawksworthii*; Table 1) [36]. Likewise, Nicaragua and the island of Hispaniola (Dominican Republic and Haiti) possess a single species—*A. hondurense* subsp. *hondurense* and *A. bicarnatum* Urban, respectively (Table 1) [1,55]. The historical and continued deforestation of highland pine forests in southern Mexico and C. America, as well as on the island of Hispaniola, has complicated efforts to delimit the geographic distributions and/or species assemblages of *Arceuthobium* across these regions [1,50].

**Figure 1:**
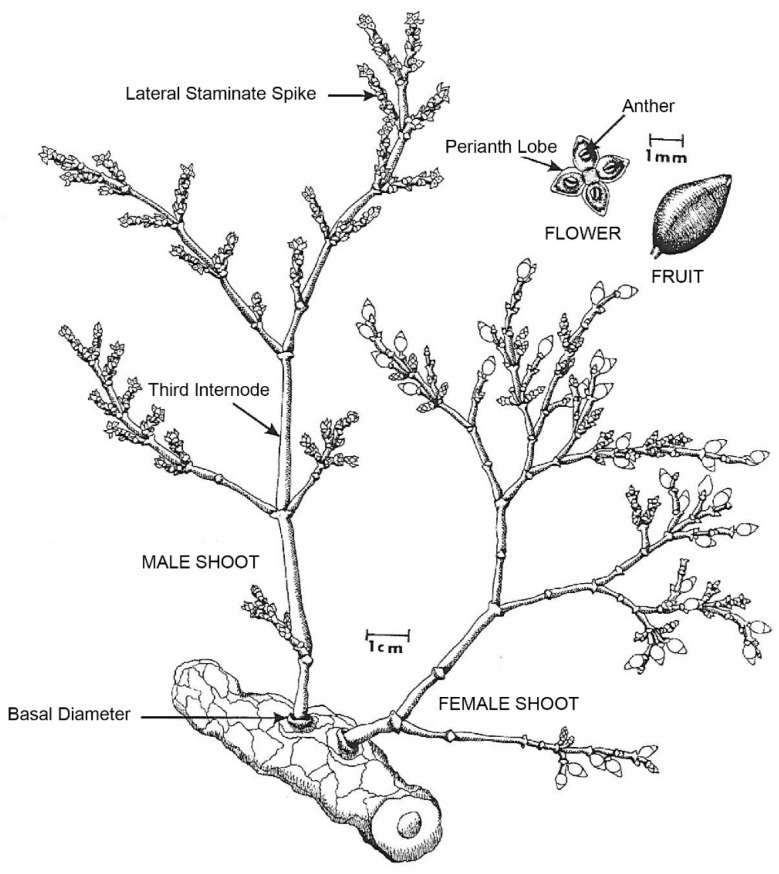
Generic illustration of the morphology for male and female plants (shoots) of *Arceuthobium*—dwarf mistletoe.

In addition to their herbaceous growth and exclusive aerial parasitism of conifers, the genus *Arceuthobium* is distinctive, given several morphological and physiological characters (Figure 1) [1]: (1) plants dioecious, often sexually dimorphic; (2) branching habit flabellate (i.e., fan-like) or verticillate (i.e., whorled); (3) leaves, opposite, fused, and reduced to minute scales; stems minus a central vascular cylinder; (5) inflorescence, typically a spike; (6) female and male flowers simple, small, and deltate; and (7) mature fruits are bicolored and, for all but one species, seeds are dispersed by a hydrostatic ballistic mechanism. Taxa in *Arceuthobium* also exhibit varying proclivities to inciting localized and/or systemic infections, as well as varying capacities to stimulate witches’ brooms on their host(s) [1]. Lastly, *Arceuthobium* taxa have discrete host affinities, and hence, host susceptibility in forests can be ranked utilizing a five-class host susceptibility classification system [1,17]. The latter host susceptibility system is dependent on natural infections and quantified by determining the percent incidence of infection across suspected host trees in close association (<6 m) with a diagnosed *Arceuthobium* taxon occupying a severely parasitized conifer. Suspected hosts attaining >90% or 50–90% trees infected are classified as a principal or secondary host, while those attaining 5–50%, >0–<5%, or 0% trees infected are classified as an occasional, rare, or immune hosts, respectively [1,17]. The susceptibility class per host-dwarf mistletoe combination is analogous to host preference [56] as plant growth and reproduction of *Arceuthobium* on occasional and rare hosts are remarkability reduced in comparison to plant performance on principal and secondary hosts [1]. Therefore, *Arceuthobium* taxa solely parasitizing a single-principal host or those with restricted principal and secondary hosts are considered highly host-specific taxa [1].

Several morphological and physiological characteristics also unify *Arceuthobium* taxa in N. America (Figure 1). Most taxa possess decussate primary branching and flabellate secondary branching—few N. American species lack or express limited secondary branching (e.g., *A. pusillum*, *A. rubrum* Hawksw. & Wiens, *A. strictum* Hawksw. & Wiens, *A. verticilliflorum* Engelm.) and, when secondary branching is evident, only one species (*A. americanum*.) clearly demonstrates a verticillate branching habit [1,57,58]. However, Kuijt [57] determined that *A. verticilliflorum* also exhibits verticillate branching. Similarly, minus taxa with incomplete phenological information (i.e., *A. abietis*-*religiosae* Heil, *A. guatemalense* Hawksw. & Wiens; *A. yecorense* Hawksw. & Wiens) [1], the staminate flowering period for most N. American dwarf mistletoes can be generalized and grouped as either spring or summer-fall flowering with most taxa (n = 32 taxa) producing male flowers across the summer and/or fall months. The latter is particularly true for *Arceuthobium* in the western USA, where only four taxa (*A. americanum*, *A. gillii*, *A. douglasii*, and *A. vaginatum* subsp. *cryptopodum*) flower in the spring. Anthesis for the remainder of taxa in the western USA (n = 22 taxa) occurs in the summer and/or fall.

One of the most distinct and unifying characteristics of N. American *Arceuthobium* is its host specificity. Collectively, N. American *Arceuthobium* exclusively parasitize conifers in the Pinaceae, including Douglas fir (*Pseudotsuga menziesii* (Mirb.) Franco) and various species of fir (*Abies* Mill.), hemlock (*Tsuga* (Endl.) Carr.), larch (*Larix* Mill.), pine (*Pinus* L.), and spruce (*Picea* A. Dietrich) [1]. Although the latter host distribution appears broad from a host-diversity perspective, the majority of N. American *Arceuthobium* demonstrates a high degree of host specialization, as approximately two-thirds of taxa (n = 31) either parasitize only one principal host or possess only one or more principal hosts while lacking secondary, occasional, and rare hosts [1]. Moreover, for taxa with two or three principal hosts (n = 8 taxa or n = 5 taxa, respectively), the principal hosts are most often in the same genus, and individual taxa with four or more principal hosts (n = 15 taxa) separately parasitize only phylogenetically closely related true pines in the genus *Pinus* subgenus *Pinus* (i.e., the hard pines) or *Pinus* subgenus *Strobus* (i.e., the soft pines). Likewise, *Arceuthobium* taxa predominately parasitize a single host species and infrequently two host species within a forest stand, whereas the reported parasitism of three or more hosts within a stand is exceedingly rare [1]. Thus, with few exceptions (e.g., *A. laricis*, *A. microcarpum* (Engelm.) Hawksw. & Wiens, and *A. tsugense*), dwarf mistletoes are among the most host-specific mistletoes worldwide, particularly in comparison to the broad host ranges demonstrated by taxa in sister genera of *Arceuthobium* in the family Viscaceae (e.g., *Viscum* L. and *Phoradendron* Nutt.) [1,39,56,59,60,61,62].

Of the 44 *Arceuthobium* taxa presently recognized to occur in N. America, 23 of these dwarf mistletoes—such as *A. americanum*, *A. abietis*-*religiosae*, *A. divaricatum*, *A. douglasii*, and *A. vaginatum* subsp. *vaginatum* and subsp. *cryptopodum*—have host and geographic distributions, quantitative morphological and/or phenological characteristics, and molecular phylogenetic evidence that unambiguously support their classification as species or subspecies [1,3,38,63]. However, anatomical reduction, sexual dimorphism, and similarity in gross morphologies of aerial parts (e.g., shoots, staminate and pistillate flowers, and fruits) across closely related dwarf mistletoes have yielded divergent interpretations and approaches to the classification and treatment of nearly one-half of the N. American *Arceuthobium* recognized to date [1,3,14,17,39,40,60,63,64,65,66,67]. Moreover, inconsistent and incomplete sampling historically has made difficult the morphological, physiological, and/or, more recently, molecular comparison of N. American dwarf mistletoes [1,2,3,38,68,69], and hence, significant knowledge gaps remain in the morphologies, life cycles, and/or phylogenetic relationships for some of the most cryptic to the most distinct taxa. Thus, *Arceuthobium* in N. America has long been considered a taxonomically difficult group relative to their field identification and their formal classification [1,3,14,17,40].

The monograph of Hawksworth and Wiens [1]—a revision to their first monograph [17]—remains the standard and often standalone encyclopedic reference for scientists and non-scientists alike to examine all facets of the dwarf mistletoes—from anatomy, life cycles, and parasitology to formal taxonomy and early molecular studies of the group. Therefore, Hawksworth and Wiens [1] have served as the foundation upon which to further investigate the geographic and taxonomic boundaries of *Arceuthobium* in N. America, as well as analyze and refine the morphological, molecular, and physiological characters that delineate taxa. However, no publication post-1996 has integrated and summarized the nearly three decades of taxonomic and phylogenetic studies that considerably improved our understanding of the dwarf mistletoes in N. America. To that end, we discuss herein the present classification system of *Arceuthobium* and important treatments in regional N. American flora published post-1996 and, thereafter, present current phylogenetic and taxonomic data for the recognition of N. American *Arceuthobium*—building upon the work of Hawksworth and Wiens [1] while highlighting knowledge gaps to be addressed in future research. Given the large number of taxa discussed, comparisons between or among taxa were organized according to their ascribed taxonomic section [1,3,38]. Likewise, *Arceuthobium* section *Campylopoda*—the section with the greatest representation in N. America—was further sub-organized to ease comparisons between and/or among morphologically and physiologically similar taxa.

**Table 1:** Summary of Arceuthobium in North America by species, subspecies, and section following Hawksworth and Wiens [1], Nickrent et al. [3], and Schneider et al. [38]. The description year and geographic distribution are listed with a supporting reference (or supporting references). Geographic distributions are abbreviated with a three-letter country code (bold type), followed by a two-letter code (not bold) for departments, provinces, or states. a—Department, province, or state by country (three-code): **CAN** (Cananda)—AB (Alberta), BC (British Columbia), MB (Manitoba), NL (Newfoundland), NS (Nova Scotia), ON (Ontario), PE (Prince Edward Island), QC (Quebec), and SK (Saskatchewan); BLZ (Belize)—CD (Cayo District) **DOM** (Dominican Republic)—AZ (Azua), BH (Barahona), JU (San Juan), ST (Santiago), and VE (La Vega); **GTM** (Guatemala)—AV (Alta Verapaz), BV (Baja Verapaz), CM (Chimaltenango), HU (Huehuetenango), QC (Quiché), SM (San Marcos), TO (Totonicapán), and ZA (Zacapa); **HND** (Honduras)—CR (Cortés), EP (El Paraíso), FM (Francisco Morazán), LE (Lempira), and OL (Olancho); **HTI** (Haiti)—OU (Ouest); **MEX** (Mexico)—BN (Baja California), CH (Chihuahua), CS (Chiapas), CU (Coahuila), DG (Durango), DF (Distrito Federal Mexico), GE (Guerrero), HD (Hildago), JA (Jalisco), MC (Michoacan), MR (Morelos), MX (Mexico), NA (Nayarit), NL (Nuevo Leon), OA (Oaxaca), PU (Puebla), QE (Queretaro), SI (Sinaloa), SL (San Luis Potosi), SO (Sonora), TA (Tamalulipas), TL (Tlaxcala), VC (Veracruz), and ZA (Zacatecas); **NIC** (Nicaragua)—NS (Nueva Segovia); and **USA** (United States)—AK (Alaska), AZ (Arizona), CA (California), CO (Colorado), CT (Connecticut), ID (Idaho), MA (Massachusetts), ME (Maine), MI (Michigan), MN (Minnesota), MT (Montana), NH (New Hampshire), NJ (New Jersey), NM (New Mexico), NY (New York), NV (Nevada), OR (Oregon), PA (Pennsylvania); RI (Rhode Island), TX (Texas), UT (Utah), VT (Vermont), WA (Washington), WI (Wisconsin), and WY (Wyoming).

Species/Subspecies	Section	Description (yr)	Country ^a^	Reference(s)
			Dept., Prov., or State	
***A*** **. *abietinum***	*Campylopoda*	1872		Engelmann in Gray [70]
subsp. *abietinum*		2019	**USA**—CA.	Mathiasen and Kenaley [61]
subsp. *grandae*		2020	**USA**—CA; OR; WA.	Kenaley [44]
subsp. *magnificae*		2019	**USA**—CA.	Mathiasen and Kenaley [61]
subsp. *mathiasenii*		2020	**USA**—AZ; NV; UT. **MEX**—CH; DG.	Kenaley [44]
subsp. *wiensii*		2009	**USA**—CA; OR.	Mathiasen and Daugherty [71]
***A.*** ***abietis*-*religiosae***	*Americana*	1923	**MEX**—DF; HI; JA; MC; NL; PU; TA; TL.	Heil [72]; Hawksworth and Wiens [1]
***A*** **. *americanum***	*Americana*	1850	**CAN**—AB; BC; MB; ON; SK. **USA**—CA; CO; ID; MT; NV; OR; UT; WA; WY.	Engelmann in Gray [73]; Hawksworth and Wiens [1]
***A*** **. *apachecum***	*Campylopoda*	1970	**USA**—AZ; NM. **MEX**—CU.	Hawksworth and Wiens [74]; Kenaley et al. [75]
***A. bicarinatum***	*Pusilla*	1912	**DOM**—AZ; BH; JU; ST; VE. **HTI**—OU.	Urban [76]; Hawksworth and Wiens [1]
***A*** **. *blumeri***	*Campylopoda*	1913	**USA**—AZ. **MEX**—CH; DG; NL; SO.	Nelson [77]; Kenaley et al. [75]
***A*** **. *californicum***	*Campylopoda*	1970	**USA**—CA.	Hawksworth and Wiens [74]; Kenaley et al. [75]
***A*** **. *campylopodum***	*Campylopoda*	1850	**USA**—CA; ID; NV; OR; WA. **MEX**—BN.	Engelmann in Gray [73]; Mathiasen and Kenaley [39]; Kenaley and Mathiasen [40]
***A*** **. *cyanocarpum***	*Campylopoda*	1906	**USA**—CA; CO; ID; OR; MT; NV; UT; WY.	Rydberg [78]; Coulter and Nelson [79]; Kenaley et al. [75]
***A. douglasii***	*Minuta*	1878	**CAN**—BC. **USA**—AZ; CA; CO; ID; MT; NM; NV; OR; TX; UT; WA; WY. **MEX**-CH; CU; DG; NL.	Engelmann in Wheeler [80]; Hawksworth and Wiens [1]
***A. divaricatum***	*Minuta*	1878	**USA**—AZ; CA; CO; NM; NV; UT; TX. **MEX**—BN.	Engelmann in Wheeler [80]; Mathiasen et al. [81]
***A. gillii***	*Rubra*	1964	**USA**—AZ; NM. **MEX**—CH; DG; SI; SO.	Hawksworth and Wiens [35]; Kenaley and Mathiasen [54]
***A. globosum***	*Globosa*	1965		Hawksworth and Wiens [35]
subsp. *aureum*		2008	**MEX**—CS. **GTM**—AV; BV; CM; QC; ZA.	Mathiasen [53]
subsp. *globosum*		2008	**MEX**—CH; DG; JA; SO.	Hawksworth and Wiens [35]; Mathiasen [53]
subsp. *grandicaule*		2008	**MEX**—DF; GE; HD; JA; MC; MR; MX; OA; PU; TL; VC. **GTM**—CM; HU; SM; TO. **HND**—LE.	Mathiasen [53]
subsp. *petersonii*		2008	**MEX**—CS; OA.	Mathiasen [53]
***A. guatemalense***	*Penda*	1970	**MEX**—CS; OA. **GTM**—HU; TO.	Hawksworth and Wiens [74]
***A. hondurense***	*Vaginata*	1970		Hawksworth and Wiens [74])
subsp. *hawksworthii*		1994	**BLZ**—CD; **HND**—OL.	Wiens and Shaw [82]1; Mathiasen [36]
subsp. *hondurense*		1970	**MEX**—CS; OA; **HND**—CR; EP; FM; LE; **NIC**—NS.	Hawksworth and Wiens [74]; Mathiasen [36]
***A*** **. *laricis***	*Campylopoda*	1906	**CAN**—BC. **USA**—ID; MT; OR; WA.	Piper [83]; St. John [84]; Mathiasen and Kenaley [42]
***A*** **. *littorum***	*Campylopoda*	1992	**USA**—CA.	Hawksworth et al. [85]; Mathiasen and Kenaley [86]
***A*** **. *microcarpum***	*Campylopoda*	1878		Engelmann in Wheeler [80]; Hawksworth and Wiens [74]
subsp. *aristatae*		2009	**USA**—AZ.	Scott and Mathiasen [37]
subsp. *microcarpum*		1878	**USA**—AZ; NM.	Engelmann in Wheeler [80]; Hawksworth and Wiens [74]
***A*** **. *monticola***	*Campylopoda*	1992	**USA**—CA; OR.	Hawksworth et al. [85]; Kenaley et al. [75]
***A. nigrum***	*Rubra*	1965	**MEX**—CS; DG; GJ; HD; MC; OA; PU; VC; ZA.	Hawksworth and Wiens [35]; Hawksworth and Wiens [48]; Kenaley and Mathiasen [54]
***A*** **. *occidentale***	*Campylopoda*	1878	**USA**—CA.	Engelmann in Wheeler [80]; Mathiasen and Kenaley [86]
***A. pendens***	*Penda*	1980	**MEX**—PU; SL; VC.	Hawksworth and Wiens [46]; Mathiasen and Daugherty [87]
***A. pusillum***	*Pusilla*	1872	**CAN**—MB; NB; NL; NS; ON; PE; QC; SK. **USA**—CT; ME; MA; MI; MN; NH; NJ; NY; PA; RI; VT; WI.	Peck [88]; Hawksworth and Wiens [1]
***A. rubrum***	*Rubra*	1965	**MEX**—DG; OA; SI.	Hawksworth and Wiens [35]; Mathiasen et al. [52]
***A*** **. *siskiyouense***	*Campylopoda*	1992	**USA**—CA; OR.	Hawksworth et al. [85]; Mathiasen and Kenaley [86]
***A. strictum***	*Vaginata*	1965	**MEX**—DG.	Hawksworth and Wiens [35]
***A*** **. *tsugense***	*Campylopoda*	1903		Rosendahl [89]; Jones [90]
subsp. *amabilae*		2007	**USA**—OR, WA.	Mathiasen and Daugherty [91]; Mathiasen and Kenaley [43]
subsp. *contortae*		2003	**CAN**—BC. **USA**—WA.	Wass and Mathiasen [92]; Mathiasen and Kenaley [43]
subsp. *mertensianae*		1992	**USA**—CA; OR.	Hawksworth et al. [85]; Mathiasen and Kenaley [43]
subsp. *tsugense*		1903	**CAN**—BC. **USA**—AK; CA; OR; WA.	Rosendahl [89]; Jones [90]; Mathiasen and Kenaley [43]
***A*** **. *vaginatum***	*Vaginata*	1825		Berchtold and Presl [93]
subsp. *cryptopodum*		1965	**USA**—AZ; CO; NM; TX; UT. **MEX**—CH; CU; SO.	Hawksworth and Wiens [35]; Hawksworth and Wiens [1]
subsp. *durangense*		1965	**MEX**—DG; JA; SI.	Hawksworth and Wiens [35]; Hawksworth and Wiens [1]
subsp. *vaginatum*		1825	**MEX**—CH, CU; DF; DG; HD; JA; MX; NA; NL; OA; PU; QE; SI; TA; VC; ZA.	Berchtold and Presl [93]; Hawksworth and Wiens [1]
***A*** **. *verticilliflorum***	*Americana*	1880	**MEX**—DG.	Engelmann [94]; Hawksworth and Wiens [1]
***A*** **. *yecorense***	*Rubra*	1989	**MEX**—CH; DG; SO.	Hawksworth and Wiens [48]

## 2. Species and Subspecies Concepts in *Arceuthobium*

*Arceuthobium* is a taxonomically challenging genus, given the morphological similarity displayed across taxa, and hence, the characters utilized to classify taxa have changed over time [1,3,14,15,17,39,40,66]. Hawksworth and Wiens [1,17]—the principal architects of the modern classification system for *Arceuthobium* worldwide—define species in *Arceuthobium* “as population systems that exhibit suites of characteristics that remain constant within prescribed limits of variation, from generation-to-generation, on different hosts, and when they occur with other taxa.” Therefore, Hawksworth and Wiens’s species concept is a “whole-data” approach to the taxonomic recognition of dwarf mistletoes, whereby populations with multiple, consistent, and demonstratable discontinuities across morphological (e.g., basal diameter, branching habit, third internodal length and width, flower diameters and morosity, and fruit and seed dimensions) and physiological characters (e.g., flowering and seed dispersal periods, host distributions, and plant color) warrant recognition. In this regard, the species concept proposed by Hawksworth and Wiens is most similar to the unified species concept advocated by de Queiroz [95] insofar as it emphasizes no single biological or evolutionary criterion for species recognition (e.g., reproductive isolation and monophyly) but, rather, integrates traditional species criteria and all available evidence in determining species (i.e., evolutionary lineages) such as allopatry and adaptive differences in host affinities. Thus, even in situations of sympatry, morphologically similar populations of *Arceuthobium* (i.e., putative cryptic species) demonstrating quantitative and qualitative differences across multiple characters would be deserving of taxonomic recognition, if only at the subspecific rank [1,17,35,36,37,43,44,45,53,61,62,71,85,91,92,96]. Similarly, Mathiasen and Kenaley [39] supported a modified yet, aligned species concept to that proposed by Hawksworth and Wiens [1,17], whereby populations of *Arceuthobium* warrant species recognition if the taxon in question has documented differences in flowering period, geographic distributions, and/or host relationships, and, via statistical comparisons (particularly multivariate analyses), has consistent and significant differences across many morphological characters when compared to allied taxa. In stark contrast, several investigators have taken a more stringent approach to delineating species in *Arceuthobium* [3,65,66,67]—employing a typological [65] or phylogenetic species concept [3,66,67] and, hence, fueling a debate as to whether Hawksworth and Wiens’s classification system grossly overestimates inter- and intraspecific diversity within *Arceuthobium* [39,40,60].

As with species, Hawksworth and Wiens [1,17] utilized a multi-trait approach to define subspecies, yet the differences in morphological and/or physiological characters segregating subspecies are neither as numerous nor of the magnitude that delimit species. With that being said, several investigators have adopted a modified definition for subspecies, wherein they collectively recognize subspecies as dwarf mistletoe populations with fewer statistically different morphological characters and infect closely related coniferous hosts. Therefore, subspecies in *Arceuthobium* are typically applied when populations show consistent morphological variations and differential host preference(s); however, such populations maintain sufficient similarity to be considered conspecific. Several populations of N. American *Arceuthobium* (e.g., *A. abietinum*, *A. globosum*, *A. microcarpum*, *A. tsugense* and *A. vaginatum)* adhere to the latter subspecies concept [35,36,37,39,43,44,61,71,85,91,92]. Against over a century of convention and in leu of subspecies, Tiehm [97] recently recombined *A. abietinum* subsp. *mathiasenii* to *A. abietinum* var. *mathiasenii* (Kenaley) Tiehm in order to standardize the infraspecific classification system across a forthcoming taxonomic checklist for the flora of Nevada. Although Mathiasen’s dwarf mistletoe is the only known fir dwarf mistletoe in Nevada, Tiehm [97] also recombined three additional and previously described subspecies of *A. abietinum* with geographic distributions beyond Nevada to varieties to maintain consistency in the infraspecific treatment of *A. abietinum*. Per the international code of nomenclature [98], the subspecific epithets for these fir dwarf mistletoes were maintained; however, the designation and communication of these subspecies as varieties should be discouraged to avoid confusion, as neither Tiehm [97] nor any other investigator of N. American *Arceuthobium* has defined the variety concept for the genus.

A persistent reproval towards the species and subspecies concepts utilized by Hawksworth and Wiens [1,17] is the lack of experimental evidence for reproductive isolation of parapatric or sympatric *Arceuthobium*, particularly taxa in the *A. campylopodum* complex in the western USA that possess overlapping flowering times [3,64,66]. Polyploidization and hybridization are indeed two important mechanisms in the evolution and speciation of plants [99,100]. However, although the haploid chromosomal number across N. American *Arceuthobium* is equivalent (n = 14) [17], no field study or experiment has demonstrated the occurrence of hybridization, or the lack thereof, among and/or between *Arceuthobium* taxa [1,17,96]. Hawksworth and Wiens [1,17] discussed the absence of both polyploidy and natural hybridization in *Arceuthobium,* as well as the apparent limited incidence of hybridization and lack of polyploidy across allied mistletoes in Viscaceae and Loranthaceae. They pointed to several possible reasons for the apparent lack of hybridization in *Arceuthobium*, including seasonal isolation due to differences in flowering times among sympatric species, the lack of a suitable habitat for hybrid survival (i.e., hosts), or strong pre-zygotic isolating mechanisms. Likewise, they also speculated that the absence of hybridization in *Arceuthobium* explained the lack of polyploidy in the genus. However, given equivalent ploidy across *Arceuthobium*, the potential influence of homoploid hybridization on the evolutionary trajectories of dwarf mistletoes remains unknown.

## 3. Taxonomic History and Classification of N. American *Arceuthobium*

### 3.1. Early Taxonomy: Early 1800s to 1935

From a historical perspective, perusing early investigations into the systematics of dwarf mistletoes in N. America is complicated due to differences among botanists in their interpretation of relevant characters for species delineation and in their nomenclatural allegiances. Regarding the latter, novel taxa described prior to the early 1900s were most frequently ascribed to the genus *Arceuthobium*; however, the genus *Razoumofskya* Hoffm. [101] was described 11 years prior to Marschall von Bieberstein’s [102] description of *Arceuthobium*, and hence, *Razoumofskya* possessed technical priority over *Arceuthobium*. A movement towards strict nomenclatural conformity in the USA at the turn of the 20th century resulted in the widespread use of *Razoumofskya*; thereby, taxa originally described as *Arceuthobium* in the early to late 1800s were synonymized under *Razoumofskya*. However, in 1905, *Arceuthobium* was acknowledged by the International Botanical Congress (Vienna Code of 1905) as a long-established (popular) genus without priority and thereby sanctioned the conservation of *Arceuthobium* over *Razoumofskya* [103]. Yet, American botanists did not accept the conservation of *Arceuthobium* until 1930, when the Cambridge Botanical Congress—according to Chapter III, Section 3, Article 22 of the code [104]—provided protection to *Arceuthobium* (nomina generica conservanda), resulting in the formal displacement of *Razoumofskya*. Thereafter, Gill’s [14] monograph of dwarf mistletoes in the USA further codified the protection of *Arceuthobium*.

Although the nomenclatural validity of *Arceuthobium* was questioned throughout the 19th and early 20th centuries, the first formal description of a New World *Arceuthobium* was *A. vaginatum* (Willd.) Presl (Mexican dwarf mistletoe) in 1825 from the state of Veracruz, Mexico [93]. Thereafter, two periods of species discovery and subsequent taxonomic debate significantly influenced the present classification of N. American *Arceuthobium*. The first period of discovery began in 1850 when George Engelmann, in communication with Asa Gray, first described two of the most widespread and morphologically distinct species of dwarf mistletoes in N. America: *A. americanum* (lodgepole pine dwarf mistletoe) and *A. campylopodum* (western dwarf mistletoe) [73]. In the same year, Engelmann was also the first to informally delineate a subspecies, recognizing the principal *Arceuthobium* of ponderosa pine (*Pinus ponderosa* var. *scopulorum* Engelm.) in the Southwest USA as *A. cryptopodum* Engelm, which was later reclassified as *A. vaginatum* forma *cryptopodum* (Engelm.) Gill [14] and thereafter to *A. vaginatum* subsp. *cryptopodum* (southwestern dwarf mistletoe) [35]. Moreover, in quick succession, Engelmann also described six additional species prior to the turn of the 20th century: *A. abietinum* Engelm. (fir dwarf mistletoe) [70], which was invalid, and the species later gained formal recognition as *A. abietinum* Engelm. ex Munz [105]; *A. divaricatum* (pinyon dwarf mistletoe) [80], *A. douglasii* (Douglas-fir dwarf mistletoe) [80], *A. microcarpum* (=*A. douglasii* var. *microcarpum* Engelm.; western spruce dwarf mistletoe) [80], *A. occidentale* Engelm. (gray pine dwarf mistletoe) [80], and *A. verticilliflorum* (big-fruited dwarf mistletoe) [94]. Thus, Engelmann is often considered the taxonomic architect of the genus *Arceuthobium* in N. America, as all taxa described by Engelmann remain recognized today based on their host affinities, as well as their morphologic and/or phenologic discontinuities [1,40]. Furthermore, of the 10 *Arceuthobium* species described prior to the conclusion of the 19th century, Engelmann was responsible for delimiting eight species and one subspecies during that time—the only two species not contributed by Engelmann before the year 1900 were *A. pusillum* (eastern spruce dwarf mistletoe) [88] and, as noted above, *A. vaginatum* [93].

The aforementioned first period of discovery continued into the 1920s with the recognition of six additional species: *Arceuthobium abietis*-*religiosae* (Mexican fir dwarf mistletoe) in central Mexico [72], *A. bicarnatum* (Hispaniolan dwarf mistletoe) from Hispaniola [76], and, in the western USA, *A. blumeri* (Blumer’s dwarf mistletoe) [77]; *A. cyanocarpum* (limber pine dwarf mistletoe) [78,79]; *A. laricis* (larch dwarf mistletoe) [83,84], and *A. tsugense* (hemlock dwarf mistletoe) [89,90]. Therefore, by the early 1920s, 13 of 19 species presently classified in the genus *Arceuthobium* and endemic to Canada and/or the USA were described, whereas only six of 17 species presently known to occur in Mexico were known to science, including some with northern geographic distributions in the USA [1,39,40]. Likewise, it would be nearly another half-century until the first description of a dwarf mistletoe from Central America in 1970 [74]. Thus, the majority of N. American *Arceuthobium* recognized prior to the first significant taxonomic investigation into the genus by Gill [14] were endemic chiefly to the western USA [1,14].

### 3.2. The Host-Form Classification System: 1935–Early 1960s

Gill’s [14] monograph and treatment of 13 *Arceuthobium* taxa in the USA marked the conclusion to the first period of discovery of N. American dwarf mistletoes while simultaneously opening the first period of debate on the classification and taxonomic status of several *Arceuthobium*. The latter work focused entirely on dwarf mistletoes with geographic distributions in the USA, yet, by extension, included several taxa endemic to southern Canada and northern Mexico. The classification system for *Arceuthobium* proposed by Gill [14] (Table 2A) prioritized branching habit, staminate flowering period (spring versus summer-fall), fruit maturation (present or second autumn post-flowering), and host affinities as principal characters. Given such a simplified suite of categorical traits, Gill [14] pruned the number of *Arceuthobium* in the contiguous USA from 13 to five species (*A. americanum*, *A. campylopodum* sensu lato, *A. douglasii*, *A. pusillum*, *A. vaginatum* s.l.) and recombined eight previously described species to separate forms of *A. campylopodum* or *A. vaginatum*. For the latter species, Gill [14] recombined *A. cryptopodum* to *A. vaginatum* f. *cryptopodum* (Engelm.) Gill based primarily on flowering time and branching habit as well as plant basal diameter, color, and height. Likewise, Gill [14] recombined seven previously recognized species (*A. abietinum*, *A. blumeri*, *A cyanocarpum*, *A. divaricatum*, *A. laricis*, *A. microcarpum*, and *A. tsugense*)—each with discrete geographic distributions in the western USA—to separate host forms of *A. campylopodum* (e.g., *A. campylopodum* f. *abietinum* (Engelm.) Gill) whereas *A. occidentale* was placed in synonymy with *A. campylopodum* f. *typicum* Gill (Table 2A). Gill [14] justified the subordination of the latter eight species to individual forms of *A. campylopodum* by reporting that these dwarf mistletoes possessed overlapping flowering periods (summer to fall) and possessed no consistent trait(s) of taxonomic significance other than their host relationship(s). Thus, the formae of *A. campylopodum* were delimited by the host upon which they occurred regardless of geographic location. This host-form system for subdividing *A. campylopodum* provided an expeditious approach to vernacularly naming dwarf mistletoes in the western USA; however, from a taxonomic perspective, Gill’s [14] host-form classification system for *A. campylopodum* yielded artificial complexes which greatly complicated investigations into the taxonomic relationships among and taxonomic status of the taxa recombined under *A. campylopodum*. The inherent complication with the host-form system for *A. campylopodum* was that it did not account for the capacity of individual forms—i.e., previously recognized species—to cross-infect unlike hosts in different genera [1,14,40,106]. For example, four subspecies of hemlock dwarf mistletoe (*A. tsugense*) have been recognized to date [43] with each subspecies sharing a parasitic habit on mountain and/or western hemlock (*Tsuga mertensiana* (Bong.) Carr.) and/or *T*. *heterophylla* (Raf.) Sarg.), as well as varying host affinities towards one or more host species outside the genus *Tsuga*. Therefore, following the publication of Gill [14], dwarf mistletoes on mountain and western hemlock in the Pacific Northwest of the USA would be classified as *A. campylopodum* f. *tsugensis* (Rosendahl) Gill; whereas at the same locality, dwarf mistletoes affecting true fir (e.g., *Abies amabilis* Douglas ex J. Forbes or *A. procera* Rehder) with identical morphologies to f. *tsugensis* were classified as f. *abietinum* even though both forms in fact comprised a population of *A. tsugense*. Thus, the application of Gill’s [14] treatment toward the field identification and study of dwarf mistletoes in the USA was only straightforward for spring flowering taxa with discrete morphologies, geographic distributions, and host ranges (i.e., *A. americanum*, *A. douglasii*, *A. pusillum*, and *A. vaginatum*). In contrast, the relegation of summer–fall-flowering taxa to host forms of *A. campylopodum* obscured the geographic distributions and taxonomic boundaries for the great majority of *Arceuthobium* in the western USA as all individual taxa within the *A. campylopodum* complex are sympatric with at least one other taxon in the group [1,106].

Gill [14] acknowledged the use of host forms to subdivide *Arceuthobium campylopodum*—and *A. vaginatum*—was indeed an artificial stopgap system to consolidate taxonomically similar taxa until the appropriate rank of these taxa (species, subspecies, or variety) could be demonstrated by field or experimental evidence [14] (pp.146–147). Unfortunately, minus the description of *A. gillii* Hawksw. & Wiens (Gill’s dwarf mistletoe) affecting Chihuahuan pine (*Pinus leiophylla* Schlecht. & Cham. var. *chihuahuana* Shaw) in mountainous regions of southern Arizona and northern Mexico [35], no significant studies were devoted to the taxonomy of N. American *Arceuthobium* following the publication of Gill [14] and into the early 1960s. However, opposition to Gill’s treatment of *A. campylopodum* and, to a lesser extent, *A. vaginatum* was evident prior to 1965 by Kuijt [15,107], Hawksworth [108], and Hawksworth and Graham [106]. Most notably, Kuijt [15] suggested the species recognition of *A. campylopodum* and *A. vaginatum* was inappropriate, citing that morphologies and staminate flowering periods between these taxa were indistinct. Thereafter, Kuijt [107] in his review of the *Arceuthobium* in California, disagreed with Gill’s host-form concept for *A. campylopodum*, suggesting that the subdivision of *A. campylopodum* by a host resulted in artificial groupings. Kuijt [107] held that contemporary evidence (i.e., artificial inoculations, geographic sympatry, and natural host distributions) of the time indicated that *A. campylopodum* would be best treated as a single polymorphic species. Hawksworth and Graham [106] agreed with Kuijt [107] insofar as the artificial nature of the host-form system, yet they suggested that the subordinated taxa under *A. campylopodum* may indeed represent distinct taxa. Utilizing western spruce dwarf mistletoe (*A. campylopodum* f. *microcarpum*) in the USA as an example, Hawksworth and Graham [106] were the first to explain the practical difficulties of applying Gill’s host-from concept, concluding that *A. campylopodum* f. *microcarpum* sensu stricto was limited to blue spruce (*Picea pungens* Engelm.) and Engelmann spruce (*P. engelmannii* Parry ex Engelm.) in Arizona and New Mexico, whereas purported populations of f. *microcarpum* affecting spruce elsewhere in the western USA represented crossover infections by *A. americanum*, *A. douglasii*, or alternative forms of *A. campylopodum* (i.e., f. *laricis* or f. *tsugensis*). In addition, without supporting quantitative data, Hawksworth and Graham [106] argued that many forms of *A. campylopodum* possessed “distinctive features” that permitted their morphological discrimination and, in combination with host associations, could yield evidence to the taxonomic status of formae classified under *A. campylopodum*. Collectively, the work of Kuijt [15,107] and Hawksworth and Graham [106] was indicative of the debate at that time amongst botanists and forest pathologists alike on the relative significance of morphology, geographic distributions, host affinities, and minor differences in flowering times in the taxonomic evaluation of the *A. campylopodum* complex and, if consistent and taxonomically informative by form, whether such character differences warranted the resurrection of a form, or forms, to species or subspecies. Likewise, as with the uncertain status of taxa under *A. campylopodum*, Mexican dwarf mistletoes prior to the early 1960s were woefully understudied with taxonomists recognizing either one (*A. vaginatum*) [109] or five species (*A. abietis*-*religiosa*, *A. campylopodum* f. *typicum*; *A. gillii*, *A. vaginatum*, and *A. verticilliflorum*) to occur in Mexico [72,85,94,107]. Hawksworth [110] briefly described his early field studies in Mexico, where he explored the geographies, host specialization, and physiological differences among populations of *A. vaginatum* s.l. parasitizing pines (*Pinus* spp.), adding that the taxonomic identity of dwarf mistletoes parasitizing smooth-bark Mexican pine (*Pinus pseudostrobus* Lindl.), Chihuahuan pine, and Montezuma pine (*P. montezumae* Lamb.) in central Mexico were uncertain.

### 3.3. Expansion of Species Number and Competing Classification Systems: 1965–Early 2000s

The brief communications of Hawksworth [108] and Hawksworth and Graham [106] were an apparent prelude to the forthcoming second period of discovery from 1965 to 1992 which, via fundamental systematic and taxonomic studies, yielded a significant expansion in species number within and an improved classification system for N. American *Arceuthobium*. Based on historical collections and new field studies, Hawksworth and Wiens [35] published the first comprehensive treatment of *Arceuthobium* in Mexico, wherein they expanded the total number of recognized taxa to 13, including nine species and three new subspecies. Recognition criteria were based principally on host specialization (i.e., common, rare, or non-host when and where the dwarf mistletoe was abundant), morphologies (plant color and height, internodal lengths, pistillate and staminate flower morphology, and fruit and seed dimensions), and period of anthesis. Hawksworth and Wiens [35] recognized five species present in the western USA with southern geographic distributions extending into northern Mexico (*A. campylopodum* f. *blumeri* (A. Nelson) Gill and f. *typicum*; *A. douglasii*, *A. gillii*, *A. vaginatum*) while also recognizing *A. abietis*-*religiosae* and *A. verticilliflorum* as endemic to Mexico. They also separated *A. gillii* and *A. vaginatum* into two and three subspecies, respectively. *Arceuthobium gillii* Hawksw. & Wiens subsp. *nigrum* Hawksw. & Wiens (black dwarf mistletoe) was described as new, differing from the typical subspecies (Gill’s dwarf mistletoe, *A. gillii* subsp. *gillii*) by its larger, dark green-to-black plants. Similarly, *A. vaginatum* f. *cryptopodum* was recombined as *A. vaginatum* (Willd.) Presl subsp. *cryptopodum* (Engelm.) Hawksw. & Wiens while *A. vaginatum* subsp. *durangense* Hawksw. & Wiens was described as new given its bright orange plant color, differences in plant height and internodal length, fruit size, geographic isolation, and host affinities in comparison to *A. vaginatum* subsp. *cryptopodum* and subsp. *vaginatum*. In addition to the description of novel subspecies, Hawksworth and Wiens [35] also described three new species from Mexico: *A. globosum* Hawksw. & Wiens (rounded dwarf mistletoe), *A. rubrum* (ruby dwarf mistletoe), and *A. strictum* (unbranched dwarf mistletoe)—each with consistent and unique morphological, phenological, and/or host range differences when compared to other Mexican dwarf mistletoes. Shortly thereafter, Hawksworth et al. [111] reported a geographic and host range extension for pinyon pine dwarf mistletoe (*A. campylopodum* f. *divaricatum* (Engelm.) Gill) in Baja California, Mexico affecting Parry pinyon (*Pinus quadrifolia* Parl. ex Sudw.), bringing the number of *Arceuthobium* in Mexico to 14 taxa. Likewise, upon the conclusion of the 1960s, the total number of *Arceuthobium* spp. with taxonomic recognition in N. America was either 11 or 19 species, depending on taxonomic allegiance to Gill [14] and, particularly, his treatment of *A. campylopodum*. Moreover, an infrageneric classification system for *Arceuthobium* had yet to be proposed and the systematics of New and Old World dwarf mistletoes remained largely unclear.

In advance of their monographic treatment of *Arceuthobium* [17], Hawksworth and Wiens [74] reclassified nine of Gill’s host forms of *A. campylopodum* to species—including the elevation of gray pine dwarf mistletoe (*A. occidentale*) to the specific rank which was formerly subordinated under the typical form of *A. campylopodum* [14]. Therein, Hawksworth and Wiens also recognized two special forms (formae speciales) of fir dwarf mistletoe (*A. abietinum* f. sp. *concoloris* Hawksw. & Wiens and f. sp. *magnificae* Hawksw. & Wiens) and described four new N. American *Arceuthobium* (*A. apachecum* Hawksw. & Wiens (Apache dwarf mistletoe), *A. californicum* Hawksw. & Wiens (sugar pine dwarf mistletoe), *A. guatemalense* Hawksw. & Wiens (Guatemalan dwarf mistletoe), and *A. hondurense* Hawksw. & Wiens (Honduran dwarf mistletoe). The latter two species were the first two taxa to be recognized from C. America. Additionally, Hawksworth and Wiens [74] proposed the first subgeneric, sectional, and series classifications for the genus. They classified Old World taxa, as well as three N. American *Arceuthobium*, to the subgenus *Arceuthobium* Hawksw. & Wiens (*A. abietis-religiosae*, *A. americanum*, and *A. verticilliflorum*) without designating a section while assigning the remaining 25 taxa of N. American dwarf mistletoe to subgenus *Vaginata* Hawksw. & Wiens. Hawksworth and Wiens [74] also subdivided subgenus *Vaginata* into three sections: the section *Campylopoda* Hawksw. & Wiens, the section *Minuta* Hawksw. & Wiens, and the section *Vaginata* Hawksw. & Wiens. Sections *Minuta* and *Vaginata* were recognized without a series designation, whereas Hawksworth and Wiens further subdivided section *Campylopoda* into three series (the series *Campylopoda* Hawksw. & Wiens, the series *Rubra* Hawksw. & Wiens, and the series *Stricta* Hawksw. & Wiens).

The section *Minuta* and section *Vaginata* were populated with two (*A. douglasii* and *A. pusillum*) and three species (*A. gillii*, *A. globosum*, and *A. vaginatum*), respectively. The series *Stricta* was monospecific (*A. strictum*), whereas three species were assigned to series *Rubra* (*A. bicarinatum*, *A. hondurense*, and *A. rubrum*). Series *Campylopoda* was ascribed the largest number of taxa—12 species including nine species elevated from Gill’s host forms, as well as *A. apachecum*, *A. californicum*, and. *A. guatemalense*. However, Hawksworth and Wiens [74] provided no arguments or taxonomic evidence to support the elevation of Gill’s forms and the separation of *A. abietinum* into two special forms, nor did they provide the characters/traits to segregate subgenera, sections, and series. Rather, Hawksworth and Wiens [74] referred their readers to their forthcoming monograph [17]. Likewise, Hawksworth and Wiens [112] broadly presented their reasoning in assigning taxonomic ranks—particularly the importance of life cycles, host specificity, host reactions to infection, as well as the morphological, phenological, and physiological differences among taxa. Yet again, Hawksworth and Wiens [112] provided no detailed descriptions or keys to the segregation of *Arceuthobium* taxa (i.e., subgenera, section, series, species, and formae speciales) and referenced their readers to their “generic monograph in press”. Thus, Hawksworth and Wiens [74,112] only established the nomenclatural framework and the rationale for the formal description of their classification system in Hawksworth and Wiens [17].

The monograph of Hawksworth and Wiens [17] was arguably the most influential, and most controversial, published work on *Arceuthobium*, as the handbook consolidated existing knowledge on the biology, pathology, systematics, and taxonomy of the dwarf mistletoes while also providing a robust body of work to continuously update and re-evaluate taxon diversity as well as further investigate the geographic, ecological, and evolutionary relationships among *Arceuthobium* [1,45,46,47,64,82,85,96,113,114,115,116,117,118,119]. The backbone of the monograph was their formal subgeneric classification of and treatment for *Arceuthobium* based on extensive field research (>1500 collections), herbarium specimen examination, and computer-assisted analysis of morphological and chemical data. With detailed descriptions by taxon (i.e., morphology, host associations, geographic distributions, and life cycle characteristics), they recognized 25 species and 32 total taxa of N. American *Arceuthobium* (Table 2B), including five species in Canada, 17 taxa in the USA (16 species, three subspecies, and two formae speciales), and 15 taxa in Mexico (12 species and five subspecies). *Arceuthobium guatemalense* from central Guatemala and *A. hondurense* from south central Honduras were the lone taxa recognized from C. America, and, similarly, *A. bicarinatum* remained the only recognized taxon from the island of Hispaniola. Hawksworth and Wiens [17] also highlighted existing taxonomic uncertainties among N. American *Arceuthobium*, particularly within the *A. campylopodum* and *A. vaginatum* complexes, as well as the apparent intraspecific variability in host distributions and morphologies of *A. abietinum* and *A. tsugense*—wherein they recognized two forma speciales of *A. abietinum* (*A. abietinum* f. sp. *concoloris* and f. sp. *magnificae*) and suggested a similar approach for the treatment of unique populations of *A. tsugense* may be appropriate. Likewise, citing the morphological reduction, host specialization, and patterns of sympatry exhibited by *Arceuthobium*, Hawksworth and Wiens [17] emphasized that all consistent discontinuities demonstrated by taxa, particularly when on principal and secondary hosts, should be utilized in taxonomic decisions. To that end, they presented the first comprehensive classification and treatment of New World dwarf mistletoes, wherein subgenera (*Arceuthobium* and *Vaginata)* were primarily delimited according to their branching pattern (verticillate and flabellate, respectively) whereas the three sections of subgenus *Vaginata* (the section *Campylopoda*, the section *Minuta*, and the section *Vaginata*) were recognized according to differences in infection/shoot habit (localized versus systemic), plant height and internodal length, and staminate flowering period (spring vs. summer–fall). The section *Vaginata* possessed taxa endemic to only the southwestern United States, Mexico, and C. America, which commonly incite non-systemic brooms and flower in the fall–winter or spring, whereas the section *Minuta* was designated as including two taxa that often incite systematic infections and produce the most diminutive aerial shoots of all taxa in subgenus *Vaginata*. The section with the greatest representation—the section *Campylopoda*—included 19 species that characteristically form non-systemic infections on their host trees, have large aerial shoots, and flower in the summer–fall. As noted previously, the section *Campylopoda* was further divided into three series (the series *Campylopoda*, *Rubra*, and *Stricta*) and segregated according to staminate plant morphology (primary and secondary branching habit, flower merosity, plant color, and plant height). A critical review of Hawksworth and Wiens’s monograph [17] shortly followed thereafter, wherein Kuijt [64] questioned the “taxonomic judgment” of Hawksworth and Wiens [17], particularly in their treatment of the series *Campylopoda*, and suggested the generic treatment possessed artificial taxa and would be unusable by those unacquainted with *Arceuthobium*. However, despite the views of Kuijt [64], the classification system by Hawksworth and Wiens [17] was widely adopted by professional foresters, forest pathologists, and botanists throughout N. America for its practicality and ease of application [1,39,40].

Following the publication of Hawksworth and Wiens [17], considerable attention was focused on further clarifying the taxonomic diversity within the controversial *Arceuthobium campylopodum* complex, as well as *Arceuthobium* of Mexico and C. America. Hawksworth and Wiens [45] examined purported populations of *A. globosum* in Mexico, Guatemala, and Belize and therein described *A. globosum* subsp. *grandicaule* Hawksw. & Wiens (large-stemmed dwarf mistletoe) from Mexico and Guatemala as well as a new species in *Arceuthobium* section *Vaginata*—*A. aureum* Hawksw. & Wiens. They further subdivided *A. aureum* into two subspecies: *A. aureum* subsp. *aureum* Hawksw. & Wiens (golden dwarf mistletoe) and *A. aureum* subsp. *petersonii* Hawksw. & Wiens (Peterson’s dwarf mistletoe) according to plant height, geographic distribution (non-sympatry), and the capacity to incite witches’ brooms. Golden dwarf mistletoe was reported to occur in southern Guatemala and western Belize, whereas Peterson’s dwarf mistletoe was geographically constrained to southern Mexico. Continuing their field studies in central Mexico, Hawksworth and Wiens [46] also described *A. pendens* Hawksw. & Wiens (pendent dwarf mistletoe) parasitizing pinyon pines (*Pinus orizabensis* (D.K. Bailey) D.K. Bailey & Hawksw. and *P. discolor* D.K. Bailey & Hawksw.) in the states of San Luis Potosí and Veracruz, placing it in the series *Campylopoda*. The addition of these new taxa from Belize, Guatemala, and Mexico were the lone modifications to the taxonomy of the genus before the infrageneric classification for *Arceuthobium* was updated by Hawksworth and Wiens [47]. Thereafter, Frank Hawksworth—either alone or in collaboration with Del Wiens—evaluated the taxonomy of hemlock dwarf mistletoe (*A. tsugense*) endemic to the Pacific Northwest of N. America [114] and continued investigations into the species diversity of Mexican dwarf mistletoes [48,116]. Upon evaluating the hemlock dwarf mistletoes, Hawksworth [114] divided *A. tsugense* into three different races according to principal host—the mountain hemlock race, the shore pine (*P. contorta* Douglas ex Loudon var. *contorta*) race, and the western hemlock race—whereas Hawksworth and Wiens [48] added two new species (*A. oaxacanum* Hawksw. & Wiens and *A. yecorense*) to the expansive assemblage of *Arceuthobium* taxa in northern Mexico. *Arceuthobium oaxacanum* (Oaxacan dwarf mistletoe) was known prior to its description as a disjunct population of the ruby dwarf mistletoe (*A. rubrum*) on smooth-bark Mexican pine in western Oaxaca [45], whereas *A. yecorense* (Yecoran dwarf mistletoe) was previously unknown. Hawksworth and Wiens [48] also elevated two Mexican subspecies to species—*A. gillii* subsp. *nigrum* to *A. nigrum* Hawks. & Wiens, and *A. vaginatum* subsp. *durangense* to *A. durangense* Hawksw. & Wiens. (Durangan dwarf mistletoe).

At the dawn of modern molecular phylogenetics, Hawksworth et al. [85] described two new species from southern Oregon and northern California (*A. monticola* Hawksw., Wiens & Nickrent and *A. siskiyouense* Hawksw., Wiens & Nickrent.), and they described a novel species from coastal California (*A. littorum* Hawksw., Wiens & Nickrent). *Arceuthobium siskiyouense* (knobcone pine dwarf mistletoe) was previously known by Gill [14] as *A. campylopodum* f. *typicum* affecting knobcone pine (*Pinus attenuata* Lemmon). Likewise, *A. littorum* (coastal dwarf mistletoe) was first classified under the typical species of *A. campylopodum* [14] and *A. occidentale* [17,47]; whereas *A. monticola* (Western white pine dwarf mistletoe) was classified as *A. campylopodum* f. *blumeri* [14] before being separated from *A. californicum* [17,72,74]. These new species were delineated by host range, geographic distributions, and morphology as well as electrophoretic comparison to allied taxa in the *A. campylopodum* complex [119]. Likewise, based on isozyme evidence [117] and host and plant height differences, Hawksworth and colleagues [85] also segregated the three former races of hemlock dwarf mistletoes *(A*. *tsugense*) into two subspecies: *A. tsugense* subsp. *tsugense* to include the western hemlock and shore pine races, and the mountain hemlock race was formally classified as *A. tsugense* subsp. *mertensianae* Hawksw. & Nickrent. The addition of these novel Californian taxa appeared in Hawksworth and Wiens’s treatment of *Arceuthobium* in *The Jepson Manual* (TJM1) [120], wherein they recognized 14 taxa in California. Thereafter, in 1994, a rare dwarf mistletoe in Belize previously classified as *A. globosum* [17] and later as *A. aureum* subsp. *aureum* [45], was described as new—*A. hawksworthii* Wiens & Shaw (Hawksworth’s dwarf mistletoe) [82]. Thus, upon the publication of their revised and last classification of *Arceuthobium* (Table 3A), Hawksworth and Wiens [1] recognized 34 species and nearly all N. American *Arceuthobium* described to date. The revised monograph of Hawksworth and Wiens [1] provided systematic and descriptive information for each taxon, as well as detailed information on the biology, anatomy, physiology, ecological relationships, pathology, and management of dwarf mistletoes. The classification system for N. American *Arceuthobium* by Hawksworth and Wiens [1,17] provided a foundation upon which to examine species and intraspecific boundaries in the genus via molecular phylogenetic approaches, as well as large-scale analyses of morphological and physiological characters [2,3,36,37,38,42,43,44,52,53,61,62,66,69,71,75,81,86,91,92,121,122,123,124,125,126,127]. Moreover, the publication of Hawksworth and Wiens [1] in effect started the second and now persistent debate on the classification and continued species recognition of many N. American *Arceuthobium*, particularly those in the controversial section *Campylopoda* [3,39,40,60,65,66,128].

Following Hawksworth and Wiens [1], several new species and subspecies of N. American *Arceuthobium* were described, and existing classifications were revised based on morphological, phenological, and genetic evidence [3,92]. The first subspecies recognized post-Hawksworth and Wiens [1] was described by Wass and Mathiasen [92], wherein they elevated the shore pine race of hemlock dwarf mistletoe in northwestern Washington and southern British Columbia to a new subspecies (*A. tsugense* subsp. *contortae* Wass & Mathiasen). Shore pine dwarf mistletoe was segregated from western hemlock dwarf mistletoes (subsp. *tsugense*) based on its principal parasitism of shore pine and only occasionally parasitism of western hemlock, as well as differences in morphology (e.g., plant height, staminate flower diameter, third internode length) and discontinuity in peak seed dispersal [92]. Thereafter, with improved sampling, Nickrent et al. [3] expanded upon the molecular phylogeny of *Arceuthobium* in Nickrent et al. [2] and provided a phylogenetic classification based on nuclear ribosomal and plastid DNA sequence data (Table 3B). Nickrent et al. [3] demonstrated that Old and New World species were phylogenetically distinct and, hence, recognized and circumscribed all N. American *Arceuthobium* to subgenus *Vaginata*. Nickrent and colleagues also designated four new sections in subgenus *Vaginata* (section *Americana* Nickrent, section *Globosa* Nickrent, section *Penda* Nickrent, and section *Pusilla* Nickrent) without series designations while also elevating series *Rubra* and series *Stricta* recognized previously by Hawksworth and Wiens [1,17] to individual sections [3]. Likewise, Nickrent et al. [3] reclassified several taxa from section *Campylopoda* (*A. bicarinatum A*. *divaricatum*, *A. guatemalense*, and *A. hondurense*, and *A. pendens*) and removed the series designations from the section *Campylopoda*. The infrageneric reorganization proposed by Nickrent and colleagues placed *A. guatemalense* and *A. pendens* in section *Penda* and reclassified *A. bicarinatum* to section *Pusilla*, *A. divaricatum* to section *Minuta*, and *A. hondurense* to section *Vaginata* (Table 3B). The proposed phylogenetic classification recognized only monophyletic species and, hence, pruned the species number of N. American *Arceuthobium* from 34 [1] to 18 species [3], as the analysis did not support the recognition of *A. aureum*, *A. durangense*, *A. hawksworthii*, *A. oaxacanum*, *A. nigrum*, and all but two species in section *Campylopoda*—*A. blumeri* and *A. campylopodum* (Table 3B). The golden and Peterson’s dwarf mistletoes—*A. aureum* subsp. *aureum* and subsp. *petersonii*, respectively—were classified in section *Globosa* and placed into synonymy with *A. globosum* s.l., whereas *A. durangense*, *A. hawksworthii*, and *A. oaxacanum* were determined to be conspecific with the Mexican (*A. vaginatum*), the Honduran (*A. hondurense*), and the ruby dwarf mistletoe (*A. rubrum*), respectively. Mathiasen et al. [52] field studies of *A. rubrum* and *A. oaxacanum* supported the classification of *A. oaxacanum* in synonymy with *A. rubrum*. Nickrent et al. [3] also placed the black dwarf mistletoe (*A. nigrum*) under Gill’s dwarf mistletoe (*A. gillii*). Moreover, the classification of Nickrent et al. [3] did not classify subspecific taxa and, hence, the forma speciales of *A. abietinum* [17] as well as subspecies of *A. tsugense* [85,92], *A. globosum* [45], and *A. vaginatum* [35] were not recognized and were placed in synonymy with *A. campylopodum*, *A. globosum*, or *A. vaginatum* (Table 3B) [3]. The work of Nickrent and colleagues [3] provided new insights into the genetic relationships among Mexican and C. American dwarf mistletoes (e.g., *A. aureum*, *A. globosum*, and *A. hawksworthii*). However, the Nickrent et al. [3] reclassification of 11 previously recognized species under *A. campylopodum* [1] reignited the long-standing debate on the treatment of taxa in the *A. campylopodum* complex. Nickrent et al. [3] supported their reclassification of taxa in section *Campylopoda* on the basis that reproductive isolation and morphological discontinuities among taxa subordinated under *A. campylopodum* had not yet been demonstrated. Thus, across the next two decades, multiple field studies were executed to compare the host specialization, geographic distributions, and, via statistical analyses, the morphological differences among *A. campylopodum* s.s. and the 11 species Nickrent et al. [3] placed in synonymy with *A. campylopodum* s.l. [39].

**Table 3:** Classification systems of North American *Arceuthobium*: Hawksworth and Wiens [1] and Nickrent et al. [3]. A. Hawksworth and Wiens’s [1] revised classification recognized 34 species and maintained two subgenera, three sections, and three series [17,47]. B. Nickrent et al. [3] recognized eight sections and 18 species of *Arceuthobium* in N. America north of Mexico.

**A.** Hawksworth and Wiens [1], excluding Old World taxa.	**B.** Nickrent et al. [3], excluding Old World taxa
**Subgenus *Vaginata* Hawksw. & Wiens** New World species 1. *A. abietis*-*religiosae* Hiel 2. *A. americanum* Nutt. Ex Engelm. 3. *A. verticilliflorum* Engelm. **Subgenus *Vaginata* Hawksw. & Wiens** Section *Vaginata* Hawksw. & Wiens 4. *A. aureum* Hawksw. & Wiens 4a. *A. aureum* subsp. *aureum* 4b. *A. aureum* subsp. *petersonii* Hawksw. & Wiens 5. *A. durangense* (Hawksw. & Wiens) Hawksw. & Wiens [=*A. vaginatum* subsp. *durangense* Hawksw & Wiens] 6. *A. gillii* Hawksw. & Wiens [=*A. gillii* subsp. *gillii*] 7. *A. globosum* Hawksw. & Wiens 7a. *A. globosum* subsp. *globosum* 7b. *A. globosum* Hawksw. & Wiens subsp. *grandicaule* Hawksw. & Wiens 8. *A. hawksworthii* Wiens & C. G. Shaw bis 9. *A. nigrum* (Hawksw. & Wiens) Hawksw. & Wiens [=*A. gillii* subsp. *nigrum*] 10. *A. vaginatum* (Willd.) Presl 10a. *A. vaginatum* subsp. *vaginatum* 10b. *A. vaginatum* (Willd.) Presl subsp. *cryptopodum* (Engelm.) Hawksw. & Wiens 11. *A. yecorense* Hawksw. & Wiens Section *Campylopoda* Hawksw. & Wiens Series *Campylopoda* Hawksw. & Wiens 12. *A. abietinum* Engelm. ex Munz 12a. *A. abietinum* f. sp. *concoloris* Hawksw. & Wiens 12b. *A. abietinum* f. sp. *magnificae* Hawksw. & Wiens 13. *A. apachecum* Hawksw. & Wiens 14. *A. blumeri* A. Nelson 15. *A. californicum* Hawksw. & Wiens 16. *A. campylopodum* Engelm. 17. *A. cyanocarpum* (A. Nelson ex Rydberg) Coulter & Nelson 18. *A. divaricatum* Engelm. 19. *A. guatemalense* Hawksw. & Wiens 20. *A. laricis* (Piper) St. John 21. *A. littorum* Hawksw., Wiens & Nickrent 22. *A. microcarpum* (Engelm.) Hawksw. & Wiens 23. *A. monticola* Hawksw., Wiens & Nickrent 24. *A. occidentale* Engelm. 25. *A. pendens* Hawksw. & Wiens 26. *A. siskiyouense* Hawksw., Wiens & Nickrent 27. *A. tsugense* (Rosendahl) G.N. Jones 27a. *A. tsugense* subsp. *tsugense* 27b. *A. tsugense* subsp. *mertensianae* Hawksw. & Nickrent Series *Rubra* Hawksw. & Wiens 28. *A. bicarinatum* Urban 29. *A. hondurense* Hawksw. & Wiens 30. *A. oaxacanum* Hawksw. & Wiens 31. *A. rubrum* Hawksw. & Wiens Series *Stricta* Hawksw. & Wiens 32. *A. strictum* Hawksw. & Wiens Section *Minuta* Hawksw. & Wiens 33. *A. douglasii* Engelm. 34. *A. pusillum* Peck	**Subgenus *Vaginata* Hawksw. & Wiens** Section *Americana* Nickrent 1. *A. abietis*-*religiosae* Hiel 2. *A. americanum* Nutt. Ex Engelm. 3. *A. verticilliflorum* Engelm. Section *Penda* Nickrent 4. *A. guatemalense* Hawksw. & Wiens 5. *A. pendens* Hawksw. & Wiens Section *Globosa* Nickrent 6. *A. globosum* Hawksw. & Wiens [*A. globosum* subsp. *globosum*] *A. globosum* subsp. *grandicaule* Hawksw. & Wiens, *A. aureum* Hawksw. & Wiens subsp. *aureum*, *A. aureum* subsp. *petersonii* Hawksw. &Wiens] Section *Pusilla* Nickrent 7. *A. bicarinatum* Urban. 8. *A. pusillum* Peck Section *Rubra* Hawksw. & Wiens 9. *A. gillii* Hawksw. & Wiens *A. nigrum* Hawksw. &Wiens 10. *A. rubrum* Hawksw. & Wiens *A. oaxacanum* Hawksw. & Wiens 11. *A. yecorense* Hawksw. & Wiens Section *Vaginata* Hawksw. & Wiens 12. *A. hondurense* Hawksw. & Wiens 13. *A. strictum* Hawksw. & Wiens 14. *A. vaginatum* (Willd.) Presl. [*A. vaginatum* subsp. *vaginatum*] *A. durangense* Hawksw. & Wiens, *A. vaginatum* subsp. *cryptopodum* (Engelm.) Hawksw. & Wiens, Section *Minuta* Hawksw. & Wiens 15. *A. divaricatum* Engelm. 16. *A. douglasii* Engelm. Section *Campylopoda* Hawksw. & Wiens 17. *A. blumeri* A. Nelson 18. *A. campylopodum* Engelm. *A. abietinum* Hawksw. & Wiens, *A. apachecum* Hawksw. & Wiens, *A. californicum* Hawksw. & Wiens, *A. cyanocarpum* (A. Nelson ex Rydberg) Coulter & Nelson, *A. laricis* (Piper) St. John, *A. littorum* Hawksw., Wiens & Nickrent, *A. microcarpum* (Engelm.) Hawksw. & Wiens, *A. monticola* Hawksw., Wiens & Nickrent, *A. occidentale* Engelm., *A. siskiyouense* Hawksw., Wiens & Nickrent, *A. tsugense* (Rosendahl) G.N. Jones].

### 3.4. Resolving Species and Subspecies: Early 2000s–Present

Several subspecies within *Arceuthobium*, particularly in section *Campylopoda*, were described or reclassified since the early 2000s. Mathiasen and Daugherty [91] described Pacific silver fir dwarf mistletoe (*A. tsugense* subsp. *amabilae* Mathiasen & C. Daugherty) as a new subspecies of hemlock dwarf mistletoe in Oregon severely parasitizing Pacific silver fir (*Abies amabilis*), noble fir (*Abies procera*), and mountain hemlock (*Tsuga mertensiana*) while only occasionally parasitizing western hemlock (*T*. *heterophylla*). In addition to host specialization [124], the Pacific silver fir dwarf mistletoe was separated from the mountain and western hemlock dwarf mistletoes based principally on plant height, third internodal length, basal diameter, shoot color, and seed dispersal period [91]. Shortly thereafter, Mathiasen [36,53] reassessed the geographies, host distributions, morphologies, and phenologies of Hawksworth’s dwarf mistletoe (*A. hawksworthii*), as well as the golden and Peterson’s dwarf mistletoes (*A. aureum* subsp. *aureum* and subsp. *petersonii*). Combined with molecular evidence [2,3], Mathiasen [36] reclassified Hawksworth’s dwarf mistletoe to a subspecies of Honduran dwarf mistletoe (*A. hondurense* subsp. *hawksworthii* (Wiens & C.G. Shaw bis) Mathiasen), demonstrating differences in the period of anthesis and seed dispersal as well as several morphological and physiological characteristics that clearly separated *A. hondurense* subsp. *hawksworthii* from the typical species. Likewise, Mathiasen [53] combined the golden and Peterson’s dwarf mistletoe as separate subspecies of *A. globosum*—*A. globosum* subsp. *aureum* (Hawksw. & Wiens) Mathiasen and *A. globosum* subsp. *petersonii* (Hawksw. & Wiens) Mathiasen. The reclassification of the golden and Peterson’s dwarf mistletoes to individual subspecies of *A. globosum* was strongly supported with nuclear and plastid DNA evidence [3] and, as reported in Mathiasen [53], the principal characteristics separating the four subspecies of *A. globosum*—subsp. *aureum*, subsp. *globosum*, subsp. *grandicaule*, and subsp. *petersonii*—were geographic distribution, plant color, fruit and shoot dimensions, and staminate flower morphology and flowering period. The new combinations of Mexican and C. American dwarf mistletoes provided by Mathiasen [36,53] were the last amendments to the classification of *Arceuthobium* in Mexico and C. America (Table 1), and hence, since 2008, a total of 17 species and six subspecies had been recognized to occur in Mexico, whereas three species and an equivalent number of subspecies had been described from C. America.

Continuing to utilize the classification system of Hawksworth and Wiens [1] as a guide, Mathiasen and Daugherty [91] described Wien’s dwarf mistletoes (*Arceuthobium abietinum* subsp. *wiensii* Mathiasen & C. Daugherty) from the Klamath-Siskiyou Mountains in southern Oregon and northern California, USA. This rare subspecies was separated from white fir dwarf mistletoe (*A. abietinum* f. sp. *concoloris*) and red dwarf mistletoe (*A. abietinum* f. sp. *magnificae*) based on its principal parasitism of red fir (*Abies magnifica* A. Murray) and Brewer spruce (*Picea breweriana* Watson), and via a statistical comparison, smaller plant dimensions, staminate flower diameter, fruit length, and plant color [91]. In the same year, and building upon Mathiasen and Hawksworth [113], Scott and Mathiasen [37] described as new *A. microcarpum* subsp. *aristatae* J.M. Scott & Mathiasen that severely parasitizes isolated, high-elevation populations of Rockey Mountain bristlecone pine (*Pinus aristata* Engelm.) in northern Arizona, USA, segregating subsp. *aristatae* from the typical western spruce dwarf mistletoe (*A. microcarpum* subsp. *microcarpum*) that principally parasitizes Engelmann and blue spruce of the southwestern USA [1,106,129]. The bristlecone pine dwarf mistletoe was also assigned its subspecific rank and distinction from western spruce dwarf mistletoe according to its early male flowering, difference in plant color, and statistically significant differences in plant morphology (e.g., pistillate and staminate plant height).

Kuijt [65] and Nickrent [66,67] proposed divergent classifications for and treatments of many *Arceuthobium* in the western USA and north of Mexico [39,40,60]. Kuijt [65] in his treatment of *Arceuthobium* of California pruned the species number from 12 [120] to three (*A. americanum*, *A. campylopodum*, and *A. douglasii*), circumscribing pinyon dwarf mistletoe (*A. divaricatum*) and eight previously recognized species as conspecific with *A. campylopodum*. Alternatively, Nickrent [66] reclassified 12 species in section *Campylopoda* as a subspecies of *A. campylopodum*, including Blumer’s dwarf mistletoe (*A. blumeri*) which, in addition to separate host and geographic distributions, was previously determined to be morphologically [1] and phylogenetically discrete from *A. campylopodum* [3]. Nickrent [66] justified his reclassification of section *Campylopoda* taxa according to molecular evidence in Nickrent et al. [3] and taxonomic information from Hawksworth and Wiens [1], contending that taxa in said section represented ecotypes, as they demonstrate varying levels of sympatry, they possess overlapping morphologies and host distributions, and differences in flowering periods and plant dimensions were associated with elevational gradients. The subordination of *A. abietinum*, *A. microcarpum*, and *A. tsugense* to separate subspecies of *A. campylopodum* precluded the recognition of previously described and future subspecies under these taxa. Nickrent [67] would later utilize his recombinations of *A. campylopodum* in his treatment of *Arceuthobium* in the Flora of North America (FNA), wherein he recognized seven species of *Arceuthobium* north of Mexico (*A. americanum*, *A. campylopodum*, *A. divaricatum*, *A. douglasii*, *A. gillii*, *A. pusillum*, *A. vaginatum*). The work of Kuijt [65] and Nickrent [66,67] resulted in a reciprocal reprioritization of taxonomic studies of N. American *Arceuthobium*, whereby investigations post-2012—beginning with Mathiasen and Daugherty [127], as well as Kenaley and Mathiasen [54]—prioritized exploring and resolving species conflicts between Hawksworth and Wiens’s and the alternative classification systems of Kuijt [65], Nickrent et al. [3], and Nickrent [66,67] [38,42,43,44,60,61,62,69,81,86]. With expanded datasets, these later studies sought to identify and fill knowledge gaps in the taxonomic assessments, host ranges, and geographic distributions of dwarf mistletoes recognized by Hawksworth and Wiens [1], particularly *A. nigrum* and cryptic taxa in the challenging section *Campylopoda.*

The taxonomic reassessment of the *Arceuthobium campylopodum* complex began in earnest with Mathiasen and Daugherty [127] and their in-depth examination of *A. littorum* and *A. occidentale*. Hawksworth et al. [85] separated the latter two species based on electrophoretic evidence [118], plant and fruit size, host affinities, and the capacity to incite witches’ brooms on infected principal hosts; yet, as mentioned previously, molecular analysis using nuclear and chloroplast DNA failed to resolve them to species [3]. Mathiasen and Daughtery [127], however, demonstrated that several new morphological (e.g., staminate spike dimensions, petal number and size, flower diameter) and phenological characteristics (e.g., anthesis and seed dispersal periods) also delineated *A. littorum* from *A. occidentale* in addition to principal host and plant characteristics described in Hawksworth et al. [85]. Similarly, staminate flowering time (i.e., fall–early winter) and geographic distribution, as well as morphological and genetic differences, were used to support the continued species recognition of *A. nigrum* [54], separating it from *A. gillii* and countering the proposed classification of *A. nigrum* under *A. gillii* by Nickrent et al. [3]. Kenaley and Mathiasen [54] also produced the first taxonomic study of N. American *Arceuthobium* that employed multivariate statistical analyses (i.e., multivariate analysis of variance [MANOVA] and discriminant function analysis [DFA]) of male and female plant characteristics to delimit taxa while simultaneously assessing the taxonomic value of plant morphologies traditionally utilized by Hawksworth and Wiens [1] in species determination. A long-standing criticism of Hawksworth and Wiens [1,17,47] and their morphological evidence for taxonomic recognition of many N. American *Arceuthobium* was their inconsistent reporting of plant characteristics by taxon and their inconsistent use of simple statistics (e.g., means, ranges, standard errors/deviations, and/or sample sizes) in taxon descriptions while demonstrating interspecific differences among taxa. Likewise, when many of their taxonomic studies reported statistical comparisons, univariate tests and/or associated multiple comparison procedures were employed to determine statistically significant differences by plant character between or among taxa [1]. The latter under-sampling and simplified statistical approach to differentiating species was also noted as ineffective by Nickrent [66], who suggested that the adoption of multivariate statistical approaches was necessary to determine statistically valid plant characters for the differentiation of taxa. Thus, all taxonomic studies from 2013 to present possessed consistent and improved sampling of morphological characteristics throughout the geographic range of the taxa understudy and said morphological characteristics across taxa were compared individually and simultaneously via univariate and multivariate analyses, respectively [42,43,44,60,61,62,69,75,81,86]. To that end, Mathiasen and Kenaley [42,86] across two separate studies compared the morphologies and host distributions among *A. campylopodum*, *A. laricis*, and *A. tsugense* subsp. *tsugense* [42] and taxa within the *A. campylopodum*-*occidentale* complex (*A. campylopodum*, *A. occidentale*, *A. littorum*, and *A. siskiyouense*) using univariate and multivariate statistical techniques [86]. The objective of both studies was to determine whether *A. campylopodum* and associated taxa could be differentiated and classified to species by morphology alone and, hence, without consideration of principal host, geographic distribution, and, when evident, differences in staminate flowering times. The results of Mathiasen and Kenaley [42,86] clearly demonstrated that western dwarf mistletoe—*A. campylopodum*—could be reliably segregated from *A. laricis*, *A. littorum*, *A. occidentale*, *A. siskiyouense*, and *A. tsugense* via the examination of male and female plant morphologies. Likewise, the morphometric analyses of Mathiasen and Kenaley readily differentiated larch dwarf mistletoe (*A. laricis*) from western hemlock dwarf mistletoe (*A. tsugense* subsp. *tsugense*) [42] and effectively delimited coastal dwarf mistletoe (*A. littorum*), gray dwarf mistletoe (*A. occidentale*), and knobcone pine dwarf mistletoe (*A. siskiyouense*) from each other [86] while also reporting the female and male plant morphologies that contributed most to the classification of taxa to species membership.

Continuing the taxonomic reassessment of *Arceuthobium* section *Campylopoda*, Reif et al. [69] and Kenaley et al. [75] refuted the classification of the white pine dwarf mistletoes (WPDMs) as conspecific with or subspecies of *A. campylopodum* [65,66]. Reif et al. [69] applied amplified fragment length polymorphism (AFLP) and morphometric analyses to delineate three WPDMs: Apache dwarf mistletoe (*A. apachecum*), Blumer’s dwarf mistletoe (*A. blumeri*), and limber pine dwarf mistletoe (*A. cyanocarpum*). Reif and colleagues demonstrated that these WPDMs, particularly *A. blumeri*, were genetically and morphologically distinct, and hence, their continued species recognition was warranted. The species recognition of *A. cyanocarpum* and two other WPDMs (*A. californicum* and *A. monticola*), as well as several other *Arceuthobium* with geographic distributions in Californica, was also advocated for by Mathiasen and Kenaley [60] in their critique of Kuijt’s treatment of *Arceuthobium* in the second edition of the *Jepson Manual* (TJM2) [65]. Mathiasen and Kenaley [60] provided detailed keys and descriptions for the field identification of Californian *Arceuthobium*, using both morphological characters and host–mistletoe relationships. Morphological evidence against synonymizing pinyon dwarf mistletoe—*A. divaricatum*—under *A. campylopodum* by Kuijt [65] was provided shortly thereafter by Mathiasen et al. [81], which supported past molecular and morphologic studies that clearly separated *A. divaricatum* from *A. campylopodum* [1,3,68]. Afterwards, building upon the work of Reif et al. [69], Kenaley et al. [75] analyzed the plant morphologies, host and geographic distributions, and phenologies among *A. campylopodum* and all five WPDMs (*A. apachecum*, *A. blumeri*, *A. californicum*, *A. cyanocarpum*, and *A. monticola*). Kenaley et al. [75] determined that species membership to *A. campylopodum* and individual WPDM species could be assigned with high precision through a morphological comparison of field-collected female and male plants alone while also reporting that all WPDMs possessed a minimum of 18 of 20 quantitative morphological traits that were statistically different when compared directly to *A. campylopodum*. Thus, the work of Kenaley et al. [75] highlighted the importance—and efficacy—of using multiple characters and statistical methods to overcome taxonomic confusion caused by the morphological reduction and similarity among closely related *Arceuthobium*. With the publication of Kenaley et al. [75], 11 of 13 species reclassified to subspecies under *A. campylopodum* by Nickrent [66] had been demonstrated to be morphologically distinct from *A. campylopodum*; leaving only *A. abietinum*, *A. microcarpum*, *A. tsugense*, and each of their associated subspecies and/or special forms as the remaining taxa in section *Campylopoda* without direct comparisons to *A. campylopodum* and, hence, without additional justification for species and/or subspecies recognition.

Although Mathiasen and Kenaley [42] determined that western hemlock dwarf mistletoe (*Arceuthobium tsugense* subsp. *tsugense*) could be segregated from western dwarf mistletoe (*A. campylopodum)*, and larch dwarf mistletoe (*A. laricis*) via morphological analysis, the latter study lacked pairwise and multivariate comparisons among the three additional subspecies of *A. tsugense* (subsp. *amabilae*, subsp. *contortae*, and subsp. *mertensianae*) and *A. campylopodum*. Therefore, in addition to reviewing the genetic and physiological evidence for continued subspecies recognition, Mathiasen and Kenaley [43] assessed the morphologies of all four subspecies of hemlock dwarf mistletoe and compared them to western dwarf mistletoe (*A. campylopodum*). They demonstrated that all four subspecies of *A. tsugense* were morphologically distinct from one another, and each subspecies was morphologically differentiated from *A. campylopodum*. Using a similar approach, Mathiasen et al. [62], as well as Mathiasen and Kenaley [61], investigated the morphological distinctions between *A. microcarpum* and *A. campylopodum,* as well as *A. abietinum* and *A. campylopodum*, respectively. Mathiasen et al. [62] provided morphological evidence for the continued species recognition of *A. microcarpum* as this species was consistently distinguished from *A. campylopodum* based on differences in plant height, basal diameter, flower diameters, fruit and seed dimensions, and host affinities. Similarly, Mathiasen and Kenaley [61] conducted a morphological comparison of *A. abietinum* and *A. campylopodum*, demonstrating through extensive sampling and statistical analysis that the special forms and subspecies of *A. abietinum* were morphologically separable from *A. campylopodum* and thus supporting the continued recognition *A. abietinum* as a separate species. Significant morphological differences were also determined between the special forms of *A. abietinum*, f. sp. *concoloris*, and f. sp. *magnificae* and, therefore, they were recombined as separate subspecies—*A. abietinum* subsp. *abietinum* (formerly f. sp. *concoloris*) and *A. abietinum* subsp. *magnificae* Mathiasen & Kenaley (formerly f. sp. *magnificae*) [61]. The taxonomic revision of *A. abietinum* by Mathiasen and Kenaley [61] thereby yielded two additional subspecies under *A. abietinum* (subsp. *abietinum* and subsp. *magnificae*) in addition to subsp. *wiensii* [71]. Shortly thereafter, Kenaley [44] expanded upon the taxonomic framework for the fir dwarf mistletoes proposed by Mathiasen and Kenaley [61] by describing two new subspecies: *A. abietinum* subsp. *grandae* Kenaley (grand fir dwarf mistletoe) and *A. abietinum* subsp. *mathiasenii* Kenaley (Mathiasen’s dwarf mistletoe). The grand fir and Mathiasen’s dwarf mistletoe were separated from *A. abietinum* subsp. *abietinum* according to discontinuities in host associations, morphological and physiological characteristics, and geographic distributions [44]. *Arceuthobium abietinum* subsp. *grandae* was demonstrated to be a principal parasite of grand fir (*Abies grandis* (Douglas ex D. Don) Lindl.) and its hybrids (*A. grandis* × *A. concolor*) in the Pacific Northwest, exhibiting yellow–green or yellow staminate and green–brown pistillate shoots and differing from the other subspecies of *A. abietinum* in female plant height and third internode dimensions. The subspecies *mathiasenii* was found to principally parasitize Rocky Mountain white fir and Durango fir (*Abies durangensis* Mart.) in isolated populations across the southwestern USA and northern Mexico while possessing larger staminate flowers, smaller pistillate plants, and a highly variable shoot color when compared directly to the other three subspecies of *A. abietinum*. The refined classification of fir dwarf mistletoes advocated by Kenaley [44] also marked the last taxonomic study of N. American *Arceuthobium* wherein a novel taxon was described. Therefore, since 2022, 30 species and 20 subspecies have been recognized as occurring in N. America (Table 4).

## 4. Phylogenetics of N. American *Arceuthobium*

As noted in the previous sections, the best-recognized classification system for N. American *Arceuthobium* by Hawksworth and Wiens [1,17] (Table 2B and Table 3A) was largely reliant on the integrative analysis of morphological traits, host specificities, and geographic distributions demonstrated by taxa. However, since the late 1970s, the advent and advancement of molecular methods to explore taxonomic boundaries has provided novel insights into subgeneric relationships and the long-standing “species question” across cryptic taxa [2,3,38,63,68,69,117,118,119,130]. Crawford and Hawksworth [130] were the first to attempt a molecular phylogeny of New and Old World *Arceuthobium* using flavonoid chemistry, revealing that taxa in *Arceuthobium* have remarkably uniform flavonoid profiles even among taxa with divergent morphologies, geographic ranges, and host affinities. North American species classified in the subgenus *Vaginata* by Hawksworth and Wiens [17] possessed predominantly galactosides, whereas all Old World and some N. American *Arceuthobium* possessed predominately glycosides; however, a flavonoid analysis yielded no resolving power and was unable to segregate sections or series proposed by Hawksworth and Wiens [17,47] and provided limited resolution at the specific level. In contrast, a series of studies utilizing isozyme analysis revealed populations of N. American *Arceuthobium* with remarkably high (e.g., *A. vaginatum*) and low (e.g., *A. abietis*-*religiosae* and *A. rubrum*) genetic variability [63,68,117,118,119]. Thus, isozyme electrophoresis was the first bona fide molecular technique upon which to refine interspecific and intraspecific boundaries within the genus, as well as investigate the sectional organization of the Hawksworth and Wiens [17,47] classification system. Isozyme evidence supported Hawksworth and Wiens’s [17,47] classification of sections *Vaginata* and *Campylopoda* [63,68] while also providing the first evidence for the future designation of sections *Rubra* and *Globosa*, as well as the reclassification of *A. divaricatum, A. pendens,* and *A. strictum* outside of section *Campylopoda* [3,17,47,63,68]. Moreover, to the species question, isozyme analysis was an effective approach to delimiting many species presently recognized today, and, hence, electrophoretic evidence was utilized as an independent method for the recognition and/or description of several novel taxa in the 1990s, particularly in the enigmatic *A. campylopodum* complex [63,117,118,119]. In section *Campylopoda*, isozyme evidence supported the segregation of *A. littorum* as a distinct species from *A. campylopodum* and *A. occidentale*, as well as the species recognition of *A. californicum, A*. *monticola*, and *A. siskiyouense* [118,119]. Similarly, electrophoretic data were used for the formal description of mountain hemlock dwarf mistletoe (*A. tsugense* subsp. *mertensianae*) [117] and the continued support for the separation of *A. gillii* and *A. nigrum* [63], which was later reaffirmed by Kenaley and Mathiasen [54] utilizing nuclear ribosomal internal transcribed spacer (ITS) data.

Improving upon Nickrent et al. [2], Nickrent et al. [3] provided the first comprehensive molecular phylogeny of 42 recognized species of *Arceuthobium* [1], utilizing ITS sequences and, for 34 species of N. American *Arceuthobium*, ITS and chloroplast *trnT-L-F* (cpDNA) sequence data. This study demonstrated that the Old and New World species form phylogenetically distinct lineages, indicating that the traditional subgenus *Arceuthobium*, defined by verticillate branching and including species from N. America, was paraphyletic. Thus, Nickrent et al. [3] reclassified *A. abietis*-*religiosae*, *A. americanum*, and *A. verticilliflorum*—taxa previously placed in subgenus *Arceuthobium* by Hawksworth and Wiens [1]—into subgenus *Vaginata*, section *Americana*. The work of Nickrent and colleagues also demonstrated that *A. oaxacanum* was conspecific with *A. rubrum* and that the eastern North American *A. pusillum* and *A. bicarinatum* from Hispaniola were closely related. Furthermore, an ITS-cpDNA analysis of section *Campylopoda* revealed that *A. blumeri* was phylogenetically distinct and sister to a large monophyletic polytomy consisting of *A. campylopodum* and 11 allied species previously placed in section *Campylopoda*, series *Campylopoda* [1]. Excluding *A. blumeri*, taxa placed in section *Campylopoda* by Nickrent et al. [3] possessed minimal interspecific genetic differences when comparing ITS sequences alone. Similarly, Nickrent and colleagues also reported that the *trnT-L-F* region contained approximately three times fewer phylogenetically informative characters than the ITS across taxa (349 ITS:136 *trnT-L-F* informative characters). However, emphasizing a monophyletic species concept [131], Nickrent et al. [3] reduced the number of N. American *Arceuthobium* from 34 to 18 species and revised the classification of several species to new or previously designated sections (Table 3) [1]. Nickrent and colleagues circumscribed *A. guatemalense* and *A. pendens* in section *Penda* while also reclassifying *A. bicarinatum* and *A. pusillum* to section *Pusilla*, *A. divaricatum* to section *Minuta*, and *A. hondurense* to section *Vaginata* (Table 3). Likewise, *A. aureum* was placed under *A. globosum*, and *A. rubrum* and *A. strictum*—previously assigned to section *Campylopoda* series *Rubra* and series *Stricta* by Hawksworth and Wiens [1]—were reclassified to subgenus *Vaginata*, section *Rubra* and section *Vaginata*, respectively (Table 3) [3]. Thus, Nickrent et al. [3] provided a foundational phylogeny; yet, as has been demonstrated outside the genus *Arceuthobium*, universal markers such as the ITS and *trnT-L-F* gene regions can have a poor phylogenetic signal and a limited capacity to delineate closely related plant species [132,133,134]. Likewise, past studies have also suggested that morphological, ecological, and physiological differences between and/or among closely related plants may evolve more rapidly than the ITS and other universal markers [135,136,137].

The most recent phylogenetic studies have employed methods with higher phylogenetic resolution, such as AFLP analysis [69] and large-scale genomic-skimming [38], to delineate species boundaries among closely related *Arceuthobium,* particularly those with more recent speciation events (e.g., the *A. campylopodum* complex) [1,3]. Albeit narrow in scope, Reif et al. [69] used AFLP analysis to assess the genetic differentiation among populations of three closely related and morphologically similar white pine dwarf mistletoes: *A. apachecum*, *A. blumeri*, and *A. cyanocarpum*. Their analyses revealed that only 13% of the total genetic variation was attributable to among-species differences. However, despite the low percentage of interspecific variation, neighbor-joining tree reconstruction clearly separated, with strong bootstrap support, *A. blumeri* from *A. apachecum;* whereas *A. apachecum* and *A. cyanocarpum* formed a strongly supported clade. Although closely related, principal coordinate analysis (PCoA) of genetic distances demonstrated that populations of *A. cyanocarpum* and *A. apachecum* were well differentiated. The genetic differences among these white pine dwarf mistletoes were also supported through morphological analyses reported therein and, hence, Reif and colleagues advocated that *A. apachecum*, *A. blumeri*, and *A. cyanocarpum* warranted species recognition and that future analyses using AFLP, or other rapidly evolving markers, could be utilized to effectively inspect and refine species boundaries in section *Campylopoda*.

Schneider et al. [38] executed the most robust phylogenetic study of *Arceuthobium* to date, leveraging high-throughput sequencing to assemble and analyze up to 45 kb of plastome data and 3 kb of the nuclear ribosomal cistron for all species recognized by Hawksworth and Wiens [1], as well as several subspecies described post-1996 and not included by Nickrent et al. [3]. The results of Schneider et al. [38] were complimentary to Nickrent et al. [3]; Schneider and colleagues demonstrated reciprocal monophyly of N. American and Old World lineages within the genus *Arceuthobium*—a pattern consistently observed across both nuclear and plastid genomic compartments. Likewise, Schneider et al. [38] also confirmed the monophyly of all eight sections of N. American *Arceuthobium* and, therein, all 18 species of *Arceuthobium* reported by Nickrent et al. [3] using nuclear and/or plastome data. The section *Americana*, the section *Campylopoda*, the section *Globosa*, and the section *Penda* were all well supported using both nuclear and plastome data; however, the topologies of the section *Rubra* and the section *Vaginata* were incongruent between datasets with the plastome data placing sections *Pusilla*, *Rubra*, and *Vaginata* in a large polytomy [38]. With that being said, the enhanced genomic coverage of Schneider et al. [38] in comparison to Nickrent et al. [3] yielded greater resolution for species and infraspecific taxa, including within section *Campylopoda*. Notably, both nuclear and plastome datasets strongly supported *A. blumeri* as a distinct species, placing this taxon in a monophyletic clade basal to all other *Campylopoda* taxa and thereby indicating that *A. blumeri* diverged well in advance of other members in the section [38]. Within the greater section *Campylopoda*, analyses of Schneider et al. [38] demonstrated monophyly for several taxa, including *A. apachecum*, *A. cyanocarpum*, and *A. microcarpum*. Schneider et al. [38] also identified a strong phylogenetic signal for two subspecies of the hemlock dwarf mistletoe (*A. tsugense* subsp. *tsugense* and subsp. *contortae*) when separately analyzing plastome and nuclear data; these subspecies were consistently placed in a well-supported clade that also included an accession of the larch dwarf mistletoe (*A. laricis*). Moreover, an analysis of the plastome supported the grouping of western spruce dwarf mistletoe (*A. microcarpum* subsp. *microcarpum*)—a species endemic to the southwestern USA—with the lone accession of Mathisen’s dwarf mistletoe (*A. abietinum* subsp. *mathiasenii*), which principally parasitizes true firs in the southwestern USA and in northern Mexico. Furthermore, Schneider and colleagues’ plastome analysis also demonstrated the close phylogenetic relationship between two subspecies of fir dwarf mistletoe—red fir (*A. abietinum* subsp. *magnificae*) and Wien’s dwarf mistletoe (*A. abietinum* subsp. *wiensii*)—as these subspecies consistently grouped together within the broader section of *Campylopoda*. Thus, Schneider et al. [38] suggested the phylogenetic patterns observed in *A. abietinum*, *A. microcarpum*, and *A. tsugense* indicated ongoing diversification, supporting the recognition of these taxa as incipient species [3,138]. Therefore, integrating all molecular evidence discussed previously with the modified classification system of Hawksworth and Wiens [1] (Table 4), N. American *Arceuthobium* consists of eight sections and 28 species with each species possessing genetic and/or phylogenetic differences that merit their rank as species. The two taxa without molecular evidence for their continued species recognition are *A. occidentale* (gray pine dwarf mistletoe) and *A. laricis* (larch dwarf mistletoe). However, these taxa can be readily segregated from each other and *A. campylopodum* s.s. by host specialization and morphological traits—traits that may be equally [95] or more precise indicators of evolutionary divergence than DNA sequences alone [135,136,137]. Thus, given our present understanding of the phylogenetics of N. American *Arceuthobium*, future investigations utilizing whole-genome sequencing and population-level genotyping approaches should be executed to detect finer-scale genetic variation, clarify species boundaries, and assess hybridization and introgression events [138,139,140,141,142,143,144]. Furthermore, integrating genomic data with ecological and morphological datasets can provide a more comprehensive understanding of host specialization and species divergence among N. American *Arceuthobium* [95,145,146].

## 5. Taxonomic Characteristics of N. American *Arceuthobium*

Across the following subsections, the comparisons of morphological characteristics, phenological data, and host range distributions among taxa were organized according to their ascribed taxonomic section (Table 4) [1,3,38]. These subsections are purposely narrative in tone with the principal objective to provide a succinct summary of the principal characteristics/traits that differentiate taxa per section while also highlighting pertinent updates for and missing data/information on taxonomic characteristics published in and after Hawksworth and Wiens [1]. Thus, the attribution of character states, host distributions, and measurements are primarily consolidated into sectional tables unless otherwise specified. This organizational strategy across the following subsections was chosen to create a more functional reference that better serves scientists engaged in the systematics of N. American *Arceuthobium*, as well as forest health specialists and forest managers charged with managing these ecologically and economically important parasitic plants.

### 5.1. Section Americana

*Arceuthobium* section *Americana* is composed of all New World species that demonstrate at least some level of verticillate branching—*A. abietis*-*religiosae* (Mexican fir dwarf mistletoe), *A. americanum* (lodgepole pine dwarf mistletoes), and *A. verticilliflorum* (big-fruited dwarf mistletoe; Table 5). Isozyme and DNA-based phylogenetic analyses have consistently and strongly supported this section and the recognition of these taxa as distinct species [3,38,63]. Moreover, given their geographic distributions (Table 1) and host affinities (Table 4), these species are some of the most easily determined taxa of *Arceuthobium* in N. America. *Arceuthobium abietis*-*religiosae* parasitizes true firs (*Abies* spp.) in central Mexico (*Abies religiosa* (Kunth) Schltdl. & Cham and *A. vejari* Mart.) with the other being Mathiasen’s dwarf mistletoe (*A. abietinum* subsp. *mathiasenii*), which has a southernmost geographic distribution terminating in northern Mexico (see section *Campylopoda*, below) [1,44]. *Arceuthobium verticilliflorum* is also a Mexican endemic (Table 1); however, it only severely parasitizes Arizona pine (*Pinus arizonica* Engelm.), Cooper’s pine (*P. cooperi* C.E. Blanco), Durango pine (*P. durangensis* Mart.), and Apache pine (*P. engelmannii* Carr.; Table 5) and has only been reported to occur in the state of Durango. Unlike Mexican fir and the big-fruited dwarf mistletoes, *A. americanum* can be found nearly wherever its principal host populations occur from western Ontario to British Columbia and south throughout the western USA to southern California and Colorado (Table 1). Lodgepole pine dwarf mistletoe also has an extensive host distribution with principal hosts including jack pine (*P. banksiana* Lamb.) and varieties of lodgepole pine (*P. contorta* Douglas ex Loudon var. *contorta*, var. *latifolia* Engelm. ex S.Watson, and var. *murrayana* (Grev. & Balf.) Critchf.; Table 5). It has a secondary host of Rocky Mountain ponderosa pine (*P. ponderosa* var. *scopulorum*), whereas whitebark pine (*P. albicaulis* Engelm.), limber pine (*P. flexilis* E. James), Jeffrey pine (*P. jeffreyi* Grev. & Balf.), and ponderosa pine (*P. ponderosa* Douglas ex C. Lawson var. *ponderosa*) are occasional hosts. Lodgepole pine dwarf mistletoe can also infect several conifers outside the genus *Pinus*; however, these are rare hosts (Table 5).

Morphologically and physiologically, *Arceuthobium americanum* is distinguished from the other members of section *Americana* by its verticillate secondary branching and shoots that are typically olive-green, yellow–green, or yellow (Table 5). Key characteristics of this species are also its fruit arrangement (verticillate) on short, reflexed pedicels and its frequent induction of systemic infections and witches’ brooms on its pine hosts. Staminate flowers of lodgepole pine dwarf mistletoe are usually 3-merous, occasionally 4-merous, and its lateral staminate buds are spherical. Anthesis for *A. americanum* occurs from early April to early June with seed dispersal occurring from late August to September. In contrast, *Arceuthobium abietis*-*religiosae* lacks secondary branching and has shoots that are olive-green when young but develop blackened variegations with age. Its staminate flowers are also primarily 3-merous, sometimes 4-merous; however, *A. abietis*-*religiosae* reportedly may have two periods of anthesis—fall and spring staminate flowering periods (Table 5). Similar to Mexican fir dwarf mistletoe, *A. verticilliflorum* also lacks secondary branching, but its shoots are yellow, yellow–green, or purplish. The big-fruited dwarf mistletoe is also unique in several morphological aspects in that it possesses 4-merous staminate flowers (occasionally 3-merous) and has the largest anthers and fruits in the genus [1]. Furthermore, unlike *A. americanum*, the fruits of *A. verticilliflorum* are borne on straightened pedicels that do not reflex upon maturity and seed dispersal is not achieved via the explosive hydrostatic ballistic mechanism demonstrated by all other *Arceuthobium,* N. American and Old World. Although taxa in section *Americana* are morphologically and geographically discrete, many plant characteristics are poorly resolved or unknown, particularly statistics for staminate flower, fruit, and seed dimensions across taxa need attention (Table 5). The period (or periods) of anthesis for Mexican fir dwarf mistletoes also remains uncertain (i.e., one vs. two staminate flowering periods annually) [1].

**Table 5:** Section *Americana*. Principal morphological and physiological characteristics of Mexican fir dwarf mistletoe (*Arceuthobium abietis*-*religiosae*), lodgepole pine dwarf mistletoe (*A. americanum*), and the big-fruited dwarf mistletoe (*A. verticilliflorum*). Morphological measurements unless indicated otherwise are means followed by ranges [min., max.]; plant heights are in cm, and all other measurements are in mm. Consecutive en dashes indicate that the statistic has not been reported, while a solitary question mark indicates the character information remains unresolved. **a**—data published by Hawksworth and Wiens [1,35]. **b**—data published by Hawksworth and Wiens [1]. **c**—host susceptibility classification is based on information by Hawksworth and Wiens [1].

Character	*Arceuthobium abietis-religiosae * ^a^	*Arceuthobium americanum * ^b^	*Arceuthobium verticilliflorum * ^a^
**Plant color**	Olive-green, black variegations with age	Olive-green, yellow–green, yellow	Yellow, yellow–green, purplish
**Branching habit**	Limited verticillate, minus secondary branching	Verticillate, plus secondary branching	Verticillate, minus secondary branching, shoots basal
**Plant height**	ca. 10.0 [---, 16.0]	ca. 5.0-9.0 [---, 30.0]	ca. 7.0 [---, 11.0]
**Basal diameter**	4.0 [2.0–10.0]	1,5 [1.0, 3.0]	3.6 [2.5, 5.0]
**Third internode length**	15.4 [8.0–24.0]	12.1 [6.0, 23.0]	3.0 [2.0, 7.0]
**Third internode width**	2.8 [1.0–4.0]	1.2 [1.0, 2.0]	3.2 [2.5, 4.5]
**Staminate flowers**			
Anthesis	March–April?; Sept.–Oct.?	Early April–early June	March–April
Perianth lobes	3-merous, occasionally 4-merous	3-merous, occasionally 4-merous	4-merous, occasionally 3-merous
Diameter	--- [2.0, 4.0]	ca. 2.0 [---, ---]	4.0 [3.5, 4.5]
Petal length	ca. 1.5 [---, ---]	ca. 1.1 [---, ---]	ca. 1.0 [---, ---]
Petal width	ca. 1.5 [---, ---]	ca. 1.0 [---, ---]	ca. 0.8 [---, ---]
Anther diameter	0.4 [---, ---]	0.6 [---, ---]	1.0 [---, ---]
**Fruit arrangement**	?	Verticillate on short, reflexed pedicels	Verticillate on short, straight pedicels
**Fruit length**	ca. 3.5 [---, ---]	4.0 [3.5, 4.5]	ca. 15.0 [---, ---]
**Fruit width**	ca. 2.0 [---, ---]	2.0 [1.5, 2.5]	ca. 10.0 [---, ---]
**Seed length**	ca. 2.2 [---, ---]	ca. 2.4 [---, ---]	ca. 11.0 [---. ---]
**Seed width**	ca. 1.0 [---, ---]	ca. 1.1 [---, ---]	ca. 6.0 [---, ---]
**Seed dispersal**	Sept.?, Oct. or November?	Late Aug.–Sept.	Sept.–Oct.
**Host susceptibility *^c^***			
Principal	*Abies religiosa* var. *religiosa*, var. *emarginata*; *A. vejari*	*Pinus banksiana*; *P. contorta* var. *contorta*, var. *latifolia*, var. *murrayana*	*Pinus arizonica*; *P. cooperi*; *P. durangensis*; *P. engelmannii*
Secondary		*Pinus ponderosa* var. *scopulorum*	
Occasional		*Pinus albicaulis*; *P. flexilis*; *P. jeffreyi*; *P. ponderosa* var. *ponderosa*	
Rare		*Picea engelmannii*; *P. glauca*; *P. mariana*; *P pungens*; *Pinus aristata*; *Pseudotsuga menziesii*	

### 5.2. Section Minuta and Section Penda

The sections *Minuta* and *Penda* collectively consist of four distinct species—two species per section: *Arceuthobium divaricatum* (pinyon dwarf mistletoe) and *A. douglasii* (Douglas-fir dwarf mistletoe) compose section *Minuta* whereas *A. guatemalense* (Guatemalan dwarf mistletoe), and *A. pendens* (pendent dwarf mistletoe) constitute section *Penda*. Since their descriptions by Engelmann [80], there has been little disagreement relative to the classification of pinyon dwarf mistletoe and Douglas-fir dwarf mistletoe as both species are genetically and phylogenetically distinct [3,38,63] with discrete geographic ranges, host distributions, and morphological as well as phenological characteristics that clearly delimit them from sympatric taxa in *Arceuthobium* subgenus *Vaginata*, including section *Campylopoda* wherein *A. divaricatum* was formerly classified (Table 1, Table 6 and Table 7) [1,3,81]. *Arceuthobium divaricatum* typically produces aerial shoots that are olive-green, green, to dark brown, whereas the plants of *A. douglasii* are consistently associated with systemic infections and are small, green–brown, and often inconspicuous at first glance among host foliage (Table 6). In terms of phenology, *A. douglasii* produces staminate flowers and mature seeds in the spring and late summer to early fall, respectively; whereas *A. divaricatum* is most often in anthesis between mid-summer and early fall and sets mature seed in late summer to mid-autumn. When flowering, Douglas-fir dwarf mistletoe has small, 3-merous (occasionally 2- or 4-merous) staminate flowers with spherical lateral buds and inner surfaces that are usually dark red. Similarly, pinyon dwarf mistletoe typically has mostly 3-merous and sometimes 4-merous staminate flowers with reddish to purple segments; however, as with shoot dimensions, *A. divaricatum* has considerably larger fruits than *A. douglasii* (Table 6).

Geographically, *Arceuthobium douglasii* has the greatest latitudinal distribution of all *Arceuthobium* extending throughout much of the native range of its principal host (*Pseudotsuga menziesii*) from southern British Columbia, Canada to northern California, USA, and throughout the Rocky Mountains into northern Mexico (Table 1 and Table 7). Douglas-fir dwarf mistletoe has no secondary hosts; yet it will occasionally infect true firs such as Pacific silver fir (*Abies amabilis*) and subalpine fir (*A. lasiocarpa* (Hook.) Nutt.) in Oregon [147], as well as corkbark fir (*A. lasiocarpa* var. *arizonica* (Merriam) Lemmon) in Arizona and New Mexico [148]. Rare hosts of *A. douglasii* include grand fir (*Abies grandis*), white fir (*A. concolor*), blue spruce (*Picea pungens*), and Engelmann spruce (*P. engelmannii*) (Table 7) [1]. The host distribution of *A. divaricatum* (Table 7), however, is remarkably different, as this dwarf mistletoe is highly specialized and exclusively parasitizes pinyon pines (*Pinus* subsection *Cembroides*) across the pinyon–juniper woodlands of the western USA (including western Texas) and northern Baja California in Mexico [1,81]. *Arceuthobium divaricatum* principally parasitizes Colorado pinyon (*Pinus edulis* Engelm.), single-leaf pinyon (*P. monophylla* Torrey & Frém.), Parry pinyon, California pinyon (*P. californiarum* D.K. Bailey), Mexican pinyon (*P. cembroides* Zucc.), and border pinyon (*P. discolor* D.K. Bailey & Hawksw.) (Table 7). Morphological characteristics for *A. divaricatum* across multiple hosts and much of its extant geographic distribution were recently assessed by Mathiasen et al. [81] and are provided in Table 6. However, as presented in Table 6, scant morphological data exists for *A. douglasii* comparing female and male plant characteristics. Given the latitudinal gradient across which this species occurs, *A. douglasii* and its principal host are an excellent system upon which to explore the biotic (i.e., reproductive isolation and host range contraction) and environmental factors (e.g., global climate change) that contribute to host–parasite evolution and parasite speciation events.

**Table 6:** Section *Minuta* and Section *Penda*. Principal morphological and physiological characters of taxa in sect. *Minuta* (*Arceuthobium divaricatum* and *A. douglasii*) and Section *Penda* (*A. guatemalense* and *A. pendens*). Morphological measurements unless indicated otherwise are means followed by ranges [min., max.]; plant heights are in cm and all other measurements are in mm.). Consecutive en dashes indicate that the statistic has not been reported. **a**—data published by Mathiasen et al. [81]. **b**—data published by Hawksworth and Wiens [1]. **c**—data published by Hawksworth and Wiens [1] with a period of anthesis modified to reflect reported staminate flowering in May by Mathiasen et al. [50]. **d**—plant color and height, perianth lobes per staminate flower, and fruit and seed dimensions published by Mathiasen and Daugherty [87]; reported seed dispersal provided by Chazaro and Olivia [149] and Mathiasen and Daugherty [87]; remaining data appear in the work of Hawksworth and Wiens [1].

Character	Section *Minuta*		Section *Penda*	
	*Arceuthobium* *divaricatum * ^a^	*Arceuthobium* *douglasii * ^b^	*Arceuthobium* *guatamalense * ^c^	*Arceuthobium* *pendens * ^d^
**Plant color**	Olive-green, green, dark brown	Green-brown (olive)	Green to purple	Light green; base dark green to dark brown with age
**Plant height**		2.0 [---, 8.0]	--- [1.0, 3.0 systemic; ---, 7.0 non-systemic]	
Female	10.9 [5.2, 22.7]			16.0 [7.0, 26.0]
Male	11.7 [5.7, 30.4]			23.0 [16.0, 32]
**Basal diameter**		1.0 [1.0, 1.5]	--- [2.0, 2.5]	2.0 [1.5, 3.5]
Female	2.6 [1.3, 5.0]			
Male	2.5 [1.6, 4.9]			
**Third internode length**		3.6 [2.0, 6.0]	11.4 [8.0, 15.0]	16.0 [12.0, 20.0]
Female	11.6 [5.8, 21.9]			
Male	11.7 [5.5, 21.8]			
**Third internode width**		ca. 1.0 [---, ---]	1.7 [1.5, 2.0]	1.5 [1.0, 2.0]
Female	1.9 [1.2, 3.2]			
Male	1.9 [1.2, 3.1]			
**Staminate flower—perianth lobes**	3-merous	3-merous; occasionally 2-, 4-merous	2- or 3-merous	3-merous; rarely 4-merous
**Anthesis**	Mid-July–late Sept.	Early March–late June	(May? -) Aug.–early Sept.	July–Sept.
**Fruit length**	4.4 [3.2, 5.3]	4.0 [3.5, 4.5]	--- [3.5, 4.0]	3.4 [2.6, 4.1]
**Fruit width**	2.6 [1.9, 3.5]	--- [1.5, 2.0]	--- [1.5, 2.0]	1.8 [1.4, 2.2]
**Seed length**	2.2 [1.6, 3.1]	2.4 [---, ---]	ca. 2.0 [---, ----]	2.3 [1.9, 2.7]
**Seed width**	1.1 [0.8, 1.3]	1.1 [---, ---]	ca. 0.8 [---, ---]	0.9 [0.7, 1.1]
**Seed dispersal**	Late Aug.–late Oct.	Late Aug.–late Sept.	Sept.	June–Sept., early Oct.

As was introduced above, the section *Penda* consists of *Arceuthobium pendens* and *A. guatemalense*. Both species are morphologically and genetically different (Table 6) [3,38] and are considered two of the rarest dwarf mistletoes in N. America due to their restricted geographic distributions and narrow host ranges (Table 1 and Table 7). The pendent dwarf mistletoe (*A. pendens*) has only been reported to parasitize pinyon pines (*Pinus discolor* and *P. orizabensis*) across a limited area encompassing Puebla, San Luis Potosi, and Veracruz in central Mexico. However, the exact host distribution and associated susceptibility classifications for *A. pendens* remain unclear (Table 7). Hawksworth and Wiens [46] first reported the host in Veracruz and Puebla as *Pinus cembroides* s.s. while later revising the host tree at these localities to Orizaba pinyon (*P. orizabensis*) [1]. Hawksworth and Wiens [46] observed *A. pendens* infecting border pinyon (*P. discolor*) in San Luis Potosi, not *P. cembroides* s.l. even though the latter purported host was present and adjacent to infected border pinyons. In contrast, there is no confusion on the host specialization of *A. guatemalense* as this dwarf mistletoe infects only Mexican white pine (*P. ayacahuite* C.Ehrenb. ex Schltdl.) in isolated populations across high-elevation pine forests of Guatemala and southern Mexico (Table 7). Upon reporting new populations of *A. guatemalense* and revisiting its type locality in the Sierra de los Cuchumatanes as well as other reported populations in Guatemala, Mathiasen and colleagues [50,51] noted that several populations of this rare dwarf mistletoe appear to have been eliminated by commercial timber harvesting. Thus, future field surveys are necessary to determine the status and present distribution of *A. guatemalense* populations in Guatemala and the Mexican states of Chiapas and Oaxaca.

In addition to specializing on pinyons in central Mexico, principal characteristics for *Arceuthobium pendens* are its long and slender shoots, pendulous habit, and its often strong sexual dimorphism (Table 6). Mature seeds are also light green and lack the yellow apical cap that is common to many *Arceuthobium*. Male and female plants of the pendent dwarf mistletoe are also light green with the basal portion of the shoots becoming dark green to dark brown with age. However, staminate plants are often considerably taller and most often associated with systemic infections (Table 6). Hawksworth and Wiens [1,46] noted the great capacity of *A. pendens* to incite witches’ brooms on its pinyon hosts. Mathiasen and Daugherty [87] noted that *A. pendens* typically incited non-systemic infections, yet, when witches’ brooms were present, brooms were most often associated with systemic infections caused by staminate plants. Likewise, Mathiasen and Daugherty [87] reported considerably different female and male plant heights and different petal numbers for staminate flowers when compared to those published by Hawksworth and Wiens [1,46]. Thus, morphological measurements for *A. pendens* presented herein (Table 6) are those reported by Mathiasen and Daugherty [87], as they are more likely reflective of this species’ morphology.

Given its single host and limited geographic distribution, no taxonomic studies have improved our understanding of the morphological characteristics and phenology of *A. guatemalense* since its description [1,17]. Thus, plant height (female and male plants), period of anthesis, and staminate flower morphology as well as the fruit and seed dimensions of *A. guatemalense* are poorly characterized and require future study (Table 7). However, in addition to its obligate parasitism of Mexican white pine, Guatemalan dwarf mistletoe can be readily differentiated from *A. pendens* by its shoot color and dimensions as well as perianth lobes per male flower and staminate flowering and seed dispersal periods (Table 7). *Arceuthobium guatemalense* displays non-pendulous shoots that are green to purple with smaller third internode dimensions when compared to *A. pendens*. *Arceuthobium guatemalense* also produces 2- and 3-merous staminate flowers, disperses seed in September and may have a continuous staminate flowering period from May–early September or possibly two distinct periods of anthesis, spring [50], and late summer (Table 6) [1,17]. Alternatively, *A. pendens* typically produces 3-merous (rarely 4-merous) male flowers, is in anthesis from mid- to late summer, and disperses seed from summer to early fall [149].

**Table 7:** Additional characteristics of *Arceuthobium* sect. *Minuta* (*A. divaricatum* and *A. douglasii*) and sect. *Penda* (*A. guatemalense* and *A. pendens*): frequency of systemic infections and witches’ brooms, host susceptibility, and sympatry between species per section. A solitary question mark indicates the character information remains unresolved. These data were published by Hawksworth and Wiens [1]. **a**—host susceptibility classification based on information by Hawksworth and Wiens [1] and Mathiasen and Daugherty [87].

Character	Section *Minuta*		Section *Penda*	
	*Arceuthobium* *divaricatum*	*Arceuthobium* *douglasii*	*Arceuthobium* *guatemalense*	*Arceuthobium* *pendens*
**Systemic infection**	No report	Common	Common	Occasional
**Witches’ broom**	Rare	Common	Common	Common
**Host susceptibility ^a^**				
Principal	*Pinus californiarum* subsp. *californiarum*, subsp. *fallax*; *P. cembroides*; *P. discolor*; *P. edulis*; *P. monophyla*; *P. quadrifolia*	*Pseudotsuga menziesii*	*Pinus ayacahuite*	*Pinus discolor*; *P. orizabensis* (*P. cembroides* subsp. *orizabensis*)
Secondary		*Abies lasiocarpa* var. *lasiocarpa*		
Occasional		*Abies amabilis*; *A. lasiocarpa* var. *arizonica*		
Rare		*Abies concolor*; *A. grandis*; *A. bifolia*; *Picea engelmannii*; *P. pungens*.		
Sympatric	Yes		No	

### 5.3. Section Globosa

The *Arceuthobium* section *Globosa* is monospecific (*A. globosum*), consisting of four subspecies (*A. globosum* subsp. *globosum*, subsp. *aureum*, subsp. *grandicaule*, and subsp. *petersonii*) that all principally parasitize three or more hosts in the genus *Pinus* section *Trifoliae* [3,53,58]. Although *A. globosum* is widespread in Mexico and C. America (Table 1), its four subspecies can be differentiated by geographic distribution, plant color, fruit and shoot dimensions, staminate flower morphology and flowering period, and host affinities [53] (Table 1 and Table 8). At present, populations of the golden dwarf mistletoe (subsp. *aureum*) and Peterson’s dwarf mistletoe (subsp. *petersonii*) are non-sympatric, and the rounded dwarf mistletoe (subsp. *globosum*) is geographically constrained to northwestern Mexico, where it affects Apache pine, Cooper’s pine, and Durango pine as principal hosts (Table 8). However, the geographic distribution of the large-stemmed dwarf mistletoe (subsp. *grandicaule*) extends from central and southern Mexico into Guatemala and Honduras, overlapping with subsp. *aureum* and subsp. *petersonii* in southeastern Mexico, where they infect some of the same hosts (Table 8). *Arceuthobium globosum* subsp. *petersonii* only principally parasitizes smooth-bark Mexican pine and Montezuma pine in Chiapas, and its parasitism of thinleaf pine (*Pinus maximinoi* H.E. Moore) within Chiapas is uncertain (Table 8) [53]. Likewise, Hawksworth and Wien [1] purported that Mexican weeping pine (*P. patula* Schiede ex Schltdl. & Cham.), Michoacán pine (*P. devoniana* Lindley), Oaxacan pine (*P. pseudostrobus* subsp. *oaxacana* (Mirov) Silba), and ocote pine (*P. oocarpa* Schiede ex Schltdl.) were principal hosts of subsp. *petersonii*. However, Michoacán pine appears to be a secondary rather than a principal host and the classification of the other purported principal hosts of subsp. *petersonii* has not been substantiated [51,53]. In Guatemala, subsp. *aureum* is principally parasitic on thinleaf pine, and it is also found on other closely related pines therein and in southern Chiapas, Mexico (Table 8).

With the largest geographic distribution, *Arceuthobium globosum* subsp. *grandicaule* is also the most morphologically distinct subspecies of *A. globosum* due to its very large, yellow–green plants (Table 8). The subspecies *grandicaule* possesses the greatest plant heights, basal diameters, third internode measurements, and staminate flower diameters of all subspecies under *A. globosum*. Moreover, the large-stemmed dwarf mistletoe has the largest fruits and initiates staminate flowering in January, which is several weeks in advance of subsp. *aureum* (mid-February) and *globosum* (March). In addition to its late summer and fall flowering period, plants of subsp. *petersonii* are typically yellow–brown and taller with larger basal diameters and third internode dimensions when compared to the vibrantly yellow (golden) plants of subsp. *aureum* (Table 8). Peterson’s dwarf mistletoe and the golden dwarf mistletoe also differ by staminate flower diameter and fruit dimensions. On the other hand, the rounded dwarf mistletoe, subsp. *globosum*, has yellow shoots that are intermediate in size with larger basal diameters when compared to subsp. *aureum* and subsp. *petersonii* while producing staminate spike widths and flower diameters nearly equivalent to subsp. *grandicaule*. Lastly, all subspecies but subsp. *globosum* readily incite large witches’ brooms on their pine host(s).

### 5.4. Section Pusilla

Hispaniolan dwarf mistletoe (*Arceuthobium bicarinatum*) and eastern spruce dwarf mistletoe (*A. pusillum*) are the only taxa in section *Pusilla* (Table 9). Hawksworth and Wiens [17] first suggested that the Hispaniolan dwarf mistletoe was most closely related to *A. hondurense* and placed these dwarf mistletoes plus *A. rubrum* in *Arceuthobium* section *Campylopoda*, series *Rubra*. Similarly, given its extreme morphological reduction and propensity to incite systemic infections, eastern spruce dwarf mistletoe was classified to section *Minuta* along with *A. douglasii* [17]. However, current phylogenetic data support the recognition of *A. bicarinatum* and *A. pusillum* as distinct species, as well as the recognition of section *Minuta* as these two species share a most recent common ancestor even though they express pronounced morphological differences (Table 9) and divergent biogeographical histories [2]. *Arceuthobium bicarinatum* is a subtropical biome endemic restricted to the island of Hispaniola, where it parasitizes Hispaniolan pine (*Pinus occidentalis* Sw.), and is separated by over 2000 km from *A. pusillum.* Eastern spruce dwarf mistletoe principally parasitizes spruce (*Picea glauca* (Moench) Voss, *P. mariana* (Mill.) Britton, Sterns & Poggenb, and *P. rubens* Sarg.) across temperate forests of northeastern and north central N. America, including the maritime provinces of Canada. Eastern larch/tamarack (*Larix laricina* (Du Roi) K. Koch) is an occasional host of *A. pusillum*, particularly in stands where eastern larch co-occurs with severely infected spruce. Witches’ brooms incited by *A. pusillum* on spruce are often large, spherical to ovate in outline and dense when compared with host branching and foliage. *Arceuthobium bicarinatum* and *A. pusillum* also can be differentiated according to plant color and height and basal diameter as well as fruit, seed, and third internode dimensions; however, no direct statistical comparisons have been executed comparing these two dwarf mistletoes. Plants of the Hispaniolan dwarf mistletoe are dark brownish red in color and reach upwards of 17 cm in height, whereas eastern sprue dwarf mistletoe produces the smallest plants of any N. American *Arceuthobium*. The shoots of *A. pusillum* typically are green–brown, solitary (rarely branched), and one cm or less in height with remarkably smaller basal diameters, internodal measurements, and fruit and seed dimensions when compared to *A. bicarinatum* and other members of the genus in N. America. For staminate flowering and seed dispersal, *A. bicarinatum* flowers in September and seed dispersal occurs in late summer where, interestingly, the uppermost node of pistillate plants is noticeably sterile [1]. Unlike the Hispaniolan dwarf mistletoe, eastern dwarf mistletoe flowers in the spring and disperses seed from late summer to early fall. However, many of the standard morphological characteristics are not well documented for *A. bicarinatum* and *A. pusillum* (e.g., fruit and seed dimensions; Table 9), and hence, future field studies should be executed to backfill missing morphological data, particularly elementary statistics for plant measurements to refine the species descriptions for these taxa.

### 5.5. Section Rubra

The section *Rubra* consists of four species—*Arceuthobium gillii* (Gill’s dwarf mistletoe), *A. nigrum* (black dwarf mistletoe), *A. rubrum* (ruby dwarf mistletoe; including *A. oaxacanum*—Oaxacan dwarf mistletoe), and *A. yecorense* (Yecoran dwarf mistletoe) [3,38]. Excluding populations of *A. gillii* in Arizona, USA, these five species are entirely endemic to Mexico (Table 1). Within this group, Gill’s dwarf mistletoe and the black dwarf mistletoe are the most morphologically and genetically similar (Table 10) [38,54]. However, their geographic distributions and staminate flowering periods do not overlap, and species membership between populations of *A. gillii* and *A. nigrum* can be determined by morphology alone [54]. Gill’s dwarf mistletoe has been reported to principally occur in the mountainous regions of southeastern Arizona and southwestern New Mexico, USA, into the state of Chihuahua, Mexico, with one population extending its geography over the Chihuahuan border into northern Durango near La Quebrada. Its host range includes Chihuahuan pine (*Pinus leiophylla* var. *chihuahuana*), Herrera’s pine (*P. herrerai* Mart.), and Lumholtz pine (*P. lumholtzii* B.L.Rob. & Fernald) (Table 11). *Arceuthobium gillii* can be readily distinguished from *A. nigrum* by its color (i.e., greenish brown, not dark brown–green to black) and significantly smaller plant dimensions (e.g., height, basal diameter, third internode measurements) and smaller plant parts (e.g., staminate flower diameter and dimensions of fruits and seeds). Moreover, Gill’s dwarf mistletoe produces green or green-yellow staminate flowers and anthesis occurs in the spring, which differs remarkably when compared to *A. nigrum* as the black dwarf mistletoe produces dark red staminate flowers in the fall. Lastly, *A. nigrum* is mainly distributed along the Central Volcanic Cordillera of central Mexico and north into the state of Durango, and although Hawksworth in 1987 reported *A. gillii* near Tepehuanes, Durango [1], these populations were confirmed to be *A. nigrum* [54]. Thus, the black dwarf mistletoe likely is the principal dwarf mistletoe on Chihuahuan pine and Lumholtz pine in Durango, Mexico (Table 11). *Arceuthobium nigrum* is also common on Aztec pine (*P. teocote* Schiede ex Schltdl. & Cham.) in Durango and severely affects several other hard pines throughout its geographic distribution, such as Chihuahuan pine, Lawson’s pine (*P. lawsonii* Roezl ex Gordon), Mexican weeping pine*,* and Oaxacan pine.

*Arceuthobium rubrum*—the ruby dwarf mistletoe—has a broad principal host range as well, parasitizing several of the same hosts as *A. nigrum* in the Sierra Madre Occidental of Durango, Mexico where they are sympatric (i.e., populations ≤ 2 km apart) [1]. The ruby dwarf mistletoe is common in Durango with Apache pine, Cooper’s pine, Durango pine, Herrera’s pine, and Aztec pine serving as its principal hosts (Table 11). *Arceuthobium rubrum* has also been reported to occur in two disjunct populations in western Oaxaca over 1000 km from the southernmost population of ruby dwarf mistletoe in Durango [1]. *Arceuthobium rubrum* severely infects Lawson’s pine, Michoacan pine, and smooth-bark Mexican pine in Oaxaca (Table 11) [45,48,52]. Although Hawksworth and Wiens ([1] indicated *A. rubrum* was present in Sinaloa, Mexico, the locality and host remain unknown. Likewise, given the wide geographic distributions of its principal hosts, *A. rubrum* likely occurs beyond Durango in Chihuahua, Nayarit, northern Jalisco, and western Zacatecas [52]. As indicated by its common name and specific epithet, the ruby dwarf mistletoe is distinguished from other taxa in section *Rubra* by its slender, reddish brown to dark red or blackish shoots and shiny, almost gloss-like, red fruits. *Arceuthobium rubrum* also produces small staminate flowers that are mostly 3-merous and have distinct petals that appear almost closed, connate, and cyanthiform during anthesis (Table 10). The ruby dwarf mistletoe also flowers in the summer to late summer, which differs from the staminate flowering period of *A. gillii* and *A. nigrum*.

It should be noted that the formal description of *Arceuthobium yecorense* provided little information regarding taxonomically informative characters [48], and no further morphologic studies have been completed for this dwarf mistletoe. Therefore, excluding molecular evidence [3,38], *A. yecorense* is principally segregated from other taxa in section *Rubra* by its slender form, yellow–green to brown shoots, and limited geographic distribution. Furthermore, anthesis and seed dispersal for *A. yecorense* have not been observed [1,48]. Hawksworth and Wiens [48] suggested that Yecoran dwarf mistletoe probably flowers in June and that it likely disperses seed in September and October, but further fieldwork is needed to confirm this. *Arceuthobium yecorense* is widespread near Yecora in Sonora, Mexico, and in eastern Sonora on Chihuahuan pine and Herrera’s pine (Table 11). The Yecoran dwarf mistletoe is also a principal parasite of Apache pine, Durango pine, and Lumholtz pine in isolated populations in western Durango (e.g., southwest of Altares) [41]. However, several principal hosts for *A. yecorense* are widespread throughout the Sierra Madre Occidental and may facilitate a “green bridge” between Sonora and Durango; thus, populations of *A. yecorense* may also exist in the pine forests of northern Durango and western Chihuahua [41].

**Table 10:** Section *Rubra*. Principal morphological and physiological characters among *Arceuthobium gillii*, *A. nigrum*, *A. rubrum*, and *A. yecorense*. Morphological measurements, unless indicated otherwise, are means followed by ranges [min., max.]; plant heights are in cm, and all other measurements are in mm. Consecutive en dashes indicate that the statistic has not been reported, while a solitary question mark indicates the character information remains unresolved. **a**—data published by Kenaley and Mathiasen [54]. **b**—data published by Hawksworth and Wiens [1] **c**—data published by Hawksworth and Wiens [48] and Hawksworth and Wiens [1].

Character	*Arceuthobium gillii * ^a^	*Arceuthobium nigrum * ^a^	*Arceuthobium rubrum * ^b^	*Arceuthobium yecorense * ^c^
**Plant color**	Green-brown	Dark brown–green, dark brown, black	Reddish brown, brown–red, dark brown, dark red, nearly black	Yellow–green, brown
**Plant height**			ca. 10.0 [---. 18.0]	ca. 12.0 [---, 17.0]
Female	14.2 [7.9, 28.4]	24.3 [10.3, 53.5]		
Male	12.3 [7.3, 21.6]	19.6 [9.3, 37.2]		
**Basal diameter**			2.4 [2.0, 3.0]	3.0 [2.0, 5.0]
Female	5.3 [2.9, 8.5]	7.8 [4.1, 13.1]		
Male	4.0 [2.4, 6.5]	7.0 [4.4, 12.5]		
**Third internode** length			6.9 [4.0, 12.0]	15.0 [10.0, 21.0]
Female	13.8 [7.8, 22.3]	16.5 [11.8, 31.8]		
Male	10.6 [6.1, 16.7]	16.8 [11.6, 28.7]		
**Third internode width**			2.3 [2.0, 3.0]	2.4 [2.0, 4.0]
Female	4.2 [2.6, 6.6]	5.5 [4.4, 9.6]		
Male	3.2 [2.1, 5.0]	4.9 [4.0, 7.8]		
**Staminate flowers**				
Anthesis	Mar.–Apr.	Sept.–Jan.	Early July–early to mid-Sept.	June?
Color	Green	Red	---	---
Spike length	15.3 [6.9, 25.6]	20.6 [8.1, 33.3]	--- [---, ---]	--- [---, ---]
Diameter			--- [1.0, 1.5]	
Diameter 3-merous	2.8 [2.1, 3.5]	3.2 [2.7, 4.0]	--- [---, ---]	--- [---, ---]
Diameter 4-merous	3.6 [3.1, 4.5]	4.8 [3.6, 5.3]	--- [---, ---]	--- [---, ---]
**Fruit color/glaucous**	Proximal portion glaucous, whitish blue	Proximal portion glaucous	Shiny red	?
**Fruit length**	5.8 [4.6, 7.2]	6.9 [5.2, 8.8]	ca. 3.5 [---, ---]	--- [---, ---]
**Fruit width**	3.6 [2.8, 4.4]	4.1 [3.4, 5.0]	ca. 2.0 [---, ---]	--- [---, ---]
**Seed length**	2.8 [2.0, 3.3]	3.1 (2.7, 3.9]	ca. 2.0 [---, ---]	--- [---, ---]
**Seed width**	1.4 [1.1, 1.6]	1.5 [1.3, 1.9]	ca. 1.0 [---, ---]	--- [---, ---]
**Seed dispersal**	Oct.	Sept.–Oct.	Mid-July–early Oct.	Sept.–Oct.?

**Table 11:** Section *Rubra*. Host susceptibility and sympatry among *Arceuthobium gillii*, *A. nigrum*, *A. rubrum*, and *A. yecorense*. These data were published by Hawksworth and Wiens [1]. Species determined to be sympatric co-occur within two kilometers of each other [1]. **a**—host susceptibility classification based on information by Hawksworth and Wiens [1] and Mathiasen et al. [41].

Character	*Arceuthobium gillii*	*Arceuthobium nigrum*	*Arceuthobium rubrum*	*Arceuthobium yecorense*
**Host susceptibility ^a^**				
Principal	*Pinus herrerai*; *P. leiophylla*, var. *chihuahuana (P. chihuahuana*); *P. lumholtzii*	*Pinus lawsonii*; *P. leiophylla* var. *leiophylla*, var. *chihuahuana* (*P. chihuahuana*); *P. lumholtzii*; *P. patula*; *P. pseudostrobus* subsp. *oaxacana* (*P. oaxacana*); *P. teocote*	*Pinus cooperi*; *P. devoniana* (*P. michoacana*); *P. durangensis*; *P. engelmannii*; *P. herrerai*; *P. lawsonii*; *P. pseudostrobus*; *P. teocote*	*Pinus durangensis*; *P. herrerai*; *P*. *leiophylla*, var. *chihuahuana* (*P. chihuahuana*); *P. lumholtzii*
Secondary				*Pinus engelmannii*
Occasional		*Pinus montezumae*; *P. pseudostrobus*	*P. pseudostrobus* subsp. *oaxacana* (*P. oaxacana*)	
Rare		*Pinus arizonica* var. *arizonica*; *P. cooperi*		
**Sympatry**	*A. yecorense*	*A. rubrum*	*A. nigrum*	*A. gillii*

### 5.6. Section Vaginata

Hawksworth and Wiens [1] utilized the available molecular, morphological, and phenological evidence for their classification of the subgenus *Vaginata*, section *Vaginata* to include four series (series *Globosa*, *Minuta*, *Rubra*, and *Vaginata*). However, Nickrent et al. [3] later reclassified the section *Vaginata* to include no series designations and only three species—*Arceuthobium hondurense* (Honduran dwarf mistletoe), *A. strictum* (unbranched dwarf mistletoe), and *A. vaginatum* (Mexican dwarf mistletoe). In so doing, *A. durangense* (Durangan dwarf mistletoe), which was first described as a subspecies of *A. vaginatum* [35] and later raised to a species based on geographic distribution as well as shoot color and size, was placed back into synonymy with *A. vaginatum* (*A. vaginatum* subsp. *durangense*). The latter reclassification of section *Vaginata* by Nickrent et al. [3], and hence, the dissolution of the intrasectional series proposed by Hawksworth and Wiens [1] was strongly supported with phylogenetic sequence data [3,38]. Thus, section *Vaginata* as demonstrated first by Nickrent et al. [3] is monophyletic and is composed of no series, three species (*A. hondurense*, *A. strictum*, and *A. vaginatum*), and five subspecies—*A. hondurense* subsp. *hawksworthii* and subsp. *hondurense*, as well as *A. vaginatum* subsp. *cryptopodum*, subsp. *durangense*, and subsp. *vaginatum*.

*Arceuthobium strictum* is the most host-specialized and, questionably, the most geographically restricted taxon in section *Vaginata*, as the unbranched dwarf mistletoe parasitizes only Chihuahuan pine in the eastern Sierra Madre Occidental in central and southern Durango, Mexico. However, given the widespread distribution of Chihuahuan pine within the region and the proximity of known Durangan populations of *A. strictum* to neighboring states, the unbranched dwarf mistletoe likely occurs on Chihuahuan pine in the states of Jalisco, Nayarit, and Zacatecas [41]. *Arceuthobium strictum* displays characteristically pale yellow to brownish shoots and male plants that are distinctively spike-like (unbranched) and produces staminate flowers that have up to seven lobes and bloom in late summer and early fall. The unbranched dwarf mistletoe also disperses seed in the fall, which differs from *A. vaginatum* subsp. *vaginatum* (Mexican dwarf mistletoe), which is abundant and widespread in Durango and throughout central and northern Mexico (Table 1). Mexican dwarf mistletoe produces some of the tallest plants in section *Vaginata*, and they are most frequently dark brown to black with long third internodes (Table 12). In addition to large and darkly colored plants, *A. vaginatum* subsp. *vaginatum* has one of the largest principal host distributions of any N. American *Arceuthobium*—infecting 11 species of hard pines and matched by only *A. globosum* subsp. *grandicaule* (Table 13). Several of the principal hosts of Mexican dwarf mistletoe (e.g., Apache pine and Cooper’s pine) are also principal hosts of *A. vaginatum* subsp. *durangense* (Durangan dwarf mistletoe) in Durango, Mexico, where the distributions of these two subspecies overlap in the vicinity of San Miguel de las Cruces (Table 13) [41]. The extant geographic distribution of Durangan dwarf mistletoe is unclear; however, it appears to be more abundant than previously reported by Hawksworth and Wiens [1]. Populations of *A. vaginatum* subsp. *durangense* occur north and west of El Salto (Durango), Mexico, and extend into eastern Sinaloa. Durangan dwarf mistletoe has also been found on Michoacán pine and Douglas pine (*Pinus douglasiana* Mart.) in the Sierra de Quila in Jalisco; thus, *A. vaginatum* subsp. *durangense* is likely in Nayarit, bridging the Sinaloan and Jalisco populations. Likewise, Hawksworth and Wiens [1] discussed the suggested reports of Durangan dwarf mistletoe parasitizing ocote pine in Durango and Jalisco and tentatively ascribed ocote pine as an occasional host of *A. vaginatum* subsp. *durangense*. There are, however, no collections or field evidence confirming this host–dwarf mistletoe relationship.

*Arceuthobium vaginatum* subsp. *durangense* and subsp. *vaginatum* are very similar morphologically and physiologically, differing by only plant color and possibly fruit length (Table 12). In contrast, *A. vaginatum* subsp. *cryptopodum* (southwestern dwarf mistletoe) can be differentiated from subsp. *durangense* and subsp. *vaginatum* by geographic and host distributions, as well as its period of anthesis. Southwestern dwarf mistletoe is common in Utah, Colorado, Arizona, New Mexico, and west Texas, USA, particularly on Rocky Mountain ponderosa pine, and in northern Mexico, where it is not sympatric with subsp. *vaginatum*. In addition to ponderosa pine, *A. vaginatum* subsp. *vaginatum* is a principal parasite of Apache pine, Arizona pine, and Durango pine, and, unlike subsp. *vaginatum*, Cooper’s pine is a secondary host, rather than a principal host, for southwestern dwarf mistletoe (Table 13). *Arceuthobium vaginatum* subsp. *cryptopodum* also frequently produces fertile staminate flowers in late spring and early summer (May–June), yet anthesis for this dwarf mistletoe reportedly initiates as early as late April and terminates as late as early July [1].

**Table 12:** Section *Vaginata*. Principal morphological and physiological characteristics of *Arceuthobium strictum*, as well as subspecies of *A. hondurense* and *A. vaginatum*. Morphological measurements, unless indicated otherwise, are means followed by ranges [min., max.]; plant heights are in cm, and all other measurements are in mm. Consecutive en dashes indicate that the statistic has not been reported. **a**—anther diameters were published by Hawksworth and Wiens [1]; the remaining data and information were published by Mathiasen [36]. **b**—internal perianth lobe color published by Mathiasen [36]; the remaining data and information were published by Hawksworth and Wiens [1]. **c**—data and information published by Hawksworth and Wiens [1,35].

Character	*Arceuthobium hondurense* subsp.		*Arceuthobium*	*Arceuthobium vaginatum* subsp.		
	*hawksworthii * ^a^	*hondurense * ^b^	*strictum * *^c^*	*cryptopodum * ^c^	*durangense * ^c^	*vaginatum * ^c^
**Plant color**	Green–brown, yellow–green, light green	Olive-brown, grayish green	Pale yellow, brownish	Predominately orange or reddish brown, occasionally nearly black	Bright orange, reddish orange	Dark brown, black, rarely reddish
**Branching habit**	Flabellate	Flabellate	Predominately unbranched	Flabellate	Flabellate	Flabellate
**Plant height**	16.0 [9.0, 34.0]	14.0 [---, 21.0]	ca. 7.0 [---, 13.0	ca. 10.0 [---, 27.0]	ca. 20.0-30.0 [---, 50.0]	ca. 20.0 [---, 55.0]
**Basal diameter**	3.8 [2.0, 8.0]	5.0 [3.0, 9.0]	3.1 [2.5, 4.0]	4.0 [2.0, 4.5]	6.0 [4.0, 8.0]	7.0 [4.0, 20.0]
**Third internode length**	12.0 [6.0, 21.0]	9.1 [7.0, 12.0]	3.6. [1.0, 8.0]	7.8 [4.0, 16.0]	17.9 [9.0, 22.0]	17.4 [5.0, 30.0]
**Third internode width**	2.8 [1.7, 4.7]	3.2 [2.5, 4.0]	2.3 [1.5, 3.5]	3.1 [2.0, 4.5]	4.5 [3.5, 6.0]	5.0 [2.5, 8.5]
**Staminate flowers**						
Anthesis	Dec.–Mar.	Aug.–Nov.	Late July–Oct.	May–June	April	Mar.–April
Perianth lobes	3-merous, occasionally 4-merous, rarely 2-merous; internally dark red to green	3-merous, occasionally 2- or 4-merous; internally dark red	3- or 4-merous, upwards of 7-merous	3-merous, occasionally 4-merous	3-merous, occasionally 4-merous	3-merous, occasionally 4-merous
Flower diameter	2.8 [2.0-3.6]	ca. 2.5 [---, ---]	ca. 3.0 [---, ---]	2.7 [2.5, 3.0]	ca. 2.5 [---, ---]	ca. 3.5 [---, ---]
Anther diameter	ca. 0.8 [---, ---]	--- [---, ---]	--- [---, ---]	0.5 [---, ---]	--- [---, ---]	0.6 [---, ---]
**Fruit length**	4.6 [3.9, 5.2]	ca. 5.5 [---, ---]	ca. 4.0 [---, ---]	5.0 [4.5, 5.5]	ca. 7.0 [---, ---]	ca. 5.5 [---, ---]
**Fruit width**	2.9 [2.3, 3.8]	ca. 3.0 [---, ---]	ca. 2.5 [---, ---]	2.5 [2.0, 3.0]	ca. 3.5 [---, --]	ca. 3.5 [---, ---]
**Seed length**	3.0 [2.6, 3.4]	ca. 3.1 [---, ---]	ca. 2.5 [---, ---]	ca. 2.7 [---, ---]	ca. 4.0 [---, ---]	--- [---, ---]
**Seed width**	1.3 [1.0, 1.6]	ca. 1.5 [---, ---]	ca. 1.0 [---, ---]	ca. 1.1 [---, ---]	ca. 1.5 [---, ---]	--- [---, ---]
**Seed dispersal**	Nov.–Jan.	Sept.–Oct.	Mid-Sept.–Oct.	Late July–early Aug.	Mid-July–Sept.	Aug.

**Table 13:** Section *Vaginata*. Host susceptibility and sympatry among *Arceuthobium strictum*, as well as subspecies of *A. hondurense* and *A. vaginatum*. These data were published by Hawksworth and Wiens [1]. Information with an accompanying question mark indicates that the character information is unresolved. The species and associated subsp. presented are not sympatric, as they do not co-occur within two kilometers of each other [1]. Host susceptibility classification based on information by Hawksworth and Wiens [1].

Character	*Arceuthobium hondurense* subsp.		*Arceuthobium*	*Arceuthobium vaginatum* subsp.		
	*hawksworthii*	*hondurense*	*strictum*	*cryptopodum*	*durangense*	*vaginatum*
**Host susceptibility**						
Principal	*Pinus caribeae* var. *hondurensis*; *Pinus oocarpa*?	*Pinus maximinoi*?; *P. oocarpa*; *Pinus tecunumanii* (*P. oocarpa* var. *ochoterenai*)	*P. leiophylla* var. *chihuahuana* (*P. chihuahuana*)	*Pinus arizonica*; *P. durangensis*; *P. englemannii*; *P. ponderosa* var. *scopulorum*	*Pinus cooperi*; *P. devoniana (P. michoacana)*; *P. douglasiana*; *P. durangensis*; *P. engelmannii*; *P. pseudostrobus*; *P. montezumae*	*Pinus arizonica*; *P. cooperi*; *P. durangensis*; *P. engelmannii*; *P. hartwegii;* *P. lawsonii*; *P. montenzumae*; *P. patula*; *P. rudis*
Secondary	*Pinus tecunumanii* (*P. oocarpa* var. *ochoterenai*)?			*Pinus cooperi*	*Pinus herrerai*	*P. teocote*
Occasional			*Pinus teocote*	*Pinus aristata*; *P. contorta* var. *latifolia*	*Pinus oocarpa*?	
Rare			*Pinus engelmannii*	*Pinus flexilis*; *P. strobiformis*		*P. culminicola*
**Sympatry**	None	None	None	None	None	None

*Arceuthobium hondurense* is segregated into two subspecies: *A. hondurense* subsp. *hondurense* (Honduran dwarf mistletoe) and subsp. *hawksworthii* (Hawksworth’s dwarf mistletoe). These dwarf mistletoes can be differentiated by geographic and host distributions, morphological differences, staminate flowering times, and seed dispersal periods. Prior to the late 1990s, Honduran dwarf mistletoe was considered rare [1]; yet, since that time, this dwarf mistletoe has now become known to occur in Mexico, Honduras, and Nicaragua [49,50,51,55,150,151,152,153,154,155]. Reports of *A. hondurense* subsp. *hondurense* in Guatemala (Department San Marcos) and El Salvador (Monte Cristo National Park), however, have not been confirmed [1]. Honduran dwarf mistletoe principally parasitizes Tecunumanii pine (*Pinus tecunumanii* F. Schwerdtf. ex Eguiluz & J.P. Perry) in Mexico, ocote pine and Tecunumanii pine in Honduras, and Tecunumannii pine in Nicaragua. Thinleaf pine has been reported to be a principal host of *A. hondurense* subsp. *hondurense* in Cusuco National Park, Honduras, but the parasitism of thinleaf pine by Honduran dwarf mistletoe at this locality has not been confirmed [50,51]. Although the geographic range of *A. hondurense* subsp. *hondurense* likely will increase with additional forest surveys, *Arceuthobium* subsp. *hawksworthii* is primarily a parasite of Caribbean pine (*P. caribaea* Morelet var. *hondurensis* (Senecl.) Barrett & Golf.) in the Mountain Pine Ridge of western Belize [49] with a remote population in north central Honduras [153]. Tecunumannii pine is also a host to *A. hondurense* subsp. *hawksworthii* in Belize, yet its susceptibility classification as a secondary host requires further assessment [51]. Sympatry between Honduran and Hawksworth’s dwarf mistletoes in Honduras has not been reported; however, they have several characteristics that separate them. Plants of *A. hondurense* subsp. *hawksworthii* are green–brown and yellow–green to light green and can exceed 34 cm in height, whereas the plants of subsp. *hondurense* are olive-brown and grayish green, and they attain a maximum height of 21 cm. Likewise, the staminate flower diameter for subsp. *hawksworthii* is greater than that for subsp. *hondurense*, and the internal perianth lobe color differs between subspecies (dark green to red vs. dark red, respectively). Male flowers of subsp. *hawksworthii* also undergo anthesis from mid-December through early March, while the period of anthesis for subsp. *hondurense* occurs from August through November. Similarly, subsp. *hawksworthii* disperses seed (November–January) after subsp. *hondurense* (September–October).

### 5.7. Section Campylopoda

Within the section *Campylopoda* (Table 4), 13 species and 11 subspecies are delineated into five groups based on their principal host(s): (1) the *Arceuthobium campylopodum*-*occidentale* complex affecting hard pines (*Pinus* subgenus *Pinus*; n = 4 species); (2) the white pine dwarf mistletoes parasitizing members of *Pinus* subgenus *Strobus* (n = 5 species); (3) the hemlock (*Tsuga* spp.) and larch (*Larix occidentalis* Nutt.) dwarf mistletoes (n = 2 species, 4 subspecies); (4) the fir dwarf mistletoes severely parasitizing hosts in the genus *Abies* (n = 1 species, 5 subspecies); and, (5) the western spruce dwarf mistletoes parasitizing members of *Picea* (n = 1 species, 2 subspecies). As discussed previously, past genetic and phylogenetic studies have supported the species recognition for 11 of the 13 species in section *Campylopoda* [3,38,69,117,118,119], including *A. abietinum* s.l., *A. apachecum*, *A. blumeri*, *A. californicum*. *A. campylopodum*, *A. cyanocarpum*, *A. littorum*, *A. microcarpum* s.l., *A. monticola*, *A. siskiyouense*, and *A. tsugense* s.l., *Arceuthobium laricis* and *A. occidentale* remain the only taxa within section *Campylopoda* without molecular evidence supporting their separation from *A. campylopodum* and, hence, their recognition at the specific rank [68,118]. Mathiasen and Kenaley [39] recently reviewed the taxonomically informative characteristics (e.g., morphologies, geographic distributions, staminate flowering periods, and principal hosts) unifying these groups within the section *Campylopoda* as well as the principal character difference among species. Thus, herein, a brief summary of key taxonomic characteristics is provided for taxa in section *Campylopoda* by host-group.

#### 5.7.1. *Arceuthobium campylopodum*-*occidentale* Complex

*Arceuthobium campylopodum* (western dwarf mistletoe), *A. littorum* (coastal dwarf mistletoe), *A. occidentale* (gray pine dwarf mistletoe), and *A. siskiyouense* (knobcone pine dwarf mistletoe) are principal parasites of hard pines (*Pinus*, subgenus *Pinus*, subsections *Ponderosae* and *Attenuatae*). Collectively, these four species are commonly referred to as the “*A. campylopodum*-*occidentale* complex”; however, despite their similarities, these species possess distinct morphological, phenological, host, and geographic differences that permit their discrimination and field diagnosis (Table 1, Table 14 and Table 15) [2,86,126,127]. Among the complex, *A. campylopodum* has the broadest geographic distribution and the only distribution that includes populations outside the western USA, spanning from Baja California, Mexico northward through California, Nevada, Oregon, Idaho, and Washington, USA to the Canadian border. In contrast, *A. littorum* and *A. occidentale* are California endemics, while *A. siskiyouense* is restricted geographically to northwestern California and southwestern Oregon [60,86,126,127]. Host affinities across the complex also differ by species (Table 14); whereby western dwarf mistletoe primarily parasitizes ponderosa pine, Jeffreyi pine, and Coulter pine (*P. coulteri* D. Don) with occasional hosts including knobcone pine, gray pine (*P. sabiniana* Douglas), and lodgepole pine [1,86]. Hawksworth and Wiens [1] reported that sugar pine (*P. lambertiana* Douglas)—a white pine—may be a rare host of *A. campylopodum* in southern Oregon; however, the population in question was confirmed to be a rare crossover infection of sugar pine by *A. abietinum* [86]. With that said, the knobcone pine dwarf mistletoe—*A. siskiyouense*—shares a principal host with western dwarf mistletoe in Jeffrey pine; however, unlike *A. campylopodum*, *A. siskiyouense* severely parasitizes knobcone pine, occasionally infects shore pine, and rarely parasitizes ponderosa pine [121]. Likewise, the host distribution of gray pine dwarf mistletoe (*A. occidentale*) and *A. campylopodum* also overlap; yet the principal host of *A. occidentale* is exclusively gray pine. Moreover, unique within the complex, coastal dwarf mistletoe (*A. littorum*) has a discrete host range that does not include hosts of *A. campylopodum*, *A. occidentale*, or *A. siskiyouense* (Table 14). This dwarf mistletoe is primarily a parasite of bishop pine (*P. muricata* D. Don) and Monterey pine (*P. radiata* D. Don).

All taxa in the *Arceuthobium campylopodum-occidentale* complex are morphologically similar (Table 15). *Arceuthobium littorum*, however, is the most morphologically distinct in this group with its dark green plants, non-branching and robust staminate spikes that are significantly longer and thicker than those of *A. campylopodum*, *A. occidentale*, and *A. siskiyouense*. The coastal dwarf mistletoe also distinctively possesses 3-, 4-, and 5-merous (rarely 6-merous) staminate flowers and dark green to red fruits [86,127]. *Arceuthobium siskiyouense* is uniquely dark brown, brown–green, red–brown, and rarely glaucous in color while producing smaller plants (height) and, typically, smaller plant parts (e.g., third internode width, staminate spike width, and the diameter of 4-merous flowers), and shorter seeds when compared to other taxa in the complex [86,126]. Plants of *A. campylopodum* have green, brown, or yellowish shoots with flabellate branching, while male plants produce nearly equivalent numbers of 3- or 4-merous flowers and its fruits are light green, closely resembling those of *A. occidentale*. However, *A. occidentale* produces plants with thinner stems (i.e., smaller basal diameters and third internode widths) when compared directly to *A. campylopodum* (Table 15). Moreover, the male flowers of gray pine dwarf mistletoe bloom from early October to early November (and occasionally into December), which differs from the summer–fall staminate flowering period of *A. campylopodum* (mid-Aug.–late Sept.), *A. littorum* (late Aug.–mid-Oct.), and *A. siskiyouense* (late July–Sept.) [86,127].

**Table 14:** Section *Campylopoda*: *Arceuthobium campylopodum*-*occidentale* complex. Host susceptibility and sympatry among *A. campylopodum*, *A. littorum*, *A. occidentale*, and *A. siskiyouense*. Species determined sympatric co-occur within two kilometers of each other [1]. These data were published originally by Mathiasen and Kenaley [86]. **a**—Host susceptibility classification based on information by Hawksworth and Wiens [1] and Mathiasen and Daugherty [126].

Character	*Arceuthobium* *campylopodum*	*Arceuthobium* *littorum*	*Arceuthobium* *occidentale*	*Arceuthobium* *siskiyouense*
**Host susceptibility ^a^**				
Principal	*Pinus coulteri*; *P. jeffreyi*; *P. ponderosa* var. *ponderosa*, var. *scopulorum*	*Pinus muricata*; *P. radiata*	*Pinus sabiniana*	*Pinus attenuata*; *P. jeffreyi*
Secondary	*Pinus attenuata*		*Pinus attenuata*; *P. coulteri*	
Occasional	*Pinus contorta* var. *latifoloia*, var. *murrayana*; *P. sabiniana*	*Pinus contorta* var. *bolanderi*	*Pinus jeffreyi*; *P. ponderosa* var. *ponderosa*	*Pinus contorta* var. *contorta*
Rare				*Pinus ponderosa* var. *ponderosa*
**Sympatry**	*A. occidentale*; *A. siskiyouense*	None	*A. campylopodum*	*A. campylopodum*

**Table 15:** Section *Campylopoda*: *Arceuthobium campylopodum*-*occidentale* complex. Principal morphological and physiological characters differentiating *A. campylopodum*, *A. littorum*, *A. occidentale*, and *A. siskiyouense*. Morphological measurements are means followed by ranges [min., max.]; plant heights are in cm, and all other measurements are in mm. These data were published originally by Mathiasen and Kenaley [86].

Character	*Arceuthobium* *campylopodum*	*Arceuthobium* *littorum*	*Arceuthobium* *occidentale*	*Arceuthobium* *siskiyouense*
**Plant color**	Olive-green, yellow	Brown–green, dark green, yellow–brown	Yellow, yellow–green, straw (males often more yellow than females)	Dark brown, brown–green, red–brown
**Plant glaucous**	Occasionally glaucous	Occasionally glaucous	Often glaucous	Seldom glaucous
**Plant height**				
Female	10.4 [3.9, 22.3]	10.3 [5.1, 18.7]	10.6 [4.9, 23.2]	9.1 [4.8, 14.8]
Male	9.7 [3.6, 21.6]	10.5 [5.4, 22.8]	10.1 [4.7, 15.6]	8.2 [4.8, 15.2]
**Basal diameter**				
Female	3.4 [1.7, 6.9]	3.9 [2.4, 6.9]	3.2 [1.7, 6.0]	3.0 [1.9, 5.7]
Male	3.2 [1.8, 6.8]	3.5 [2.5, 5.8]	3.0 [1.8, 5.4]	3.1 [1.8, 6.1]
**Third internode width**				
Female	2.5 [1.6, 3.7]	2.6 [1.9, 3.7]	2.2 [1.3, 3.5]	2.0 [1.3, 3.1]
Male	2.5 [1.4, 3.6]	2.7 [1.8, 3.6]	2.2 [1.4, 3.1]	2.1 [1.5, 2.9]
**Staminate flower**				
Anthesis (peak)	Mid-Aug.–late Sept. (late Aug.–mid-Sept.)	Late Aug.–mid-Oct. (mid- to late Sept.)	Oct.–Nov., Dec. (mid-Oct.–mid-Nov.)	Late July–Sept. (mid-Aug.)
Spike Length	12.7 [3.7, 41.0]	20.6 [6.1, 55.9]	13.9 [6.2, 33.9]	11.8 [6.1, 18.3]
Spike Width	3.0 [2.3, 4.2]	3.4 [2.1, 4.2]	2.9 [2.2, 3.9]	2.0 [1.5, 2.6]
Diameter 3-merous	3.1	3.5	3.0	3.2
Diameter 4-merous	4.2	5.2	4.1	4.5
Diameter 5-merous	None	5.7	Rare (n= 1)	None
Petal length	1.6 [0.9, 2.4]	1.9 [1.0, 2.6]	1.5 [1.0, 2.3]	1.5 [0.9, 2.2]
Petal width	1.4 [0.7, 2.4]	1.6 [0.8, 2.3]	1.3 [0.9, 2.2]	1.5 [0.8, 2.1]
Anther distance from tip	0.6 [0.2, 1.1]	0.9 [0.4, 1.4]	0.6 [0.2, 1.0]	0.8 [0.5, 1.1]
**Fruit color**	Light green	Dark green to red	Light green	Light green
**Fruit glaucous**	Lightly glaucous	Lightly glaucous	Highly glaucous	Lightly glaucous

#### 5.7.2. White Pine Dwarf Mistletoes

The five white pine dwarf mistletoes (WPDM)—*A. apachecum* (Apache dwarf mistletoe), *A. blumeri* (Blumer’s dwarf mistletoe), *A. californicum* (sugar pine dwarf mistletoe), *A. cyanocarpum* (limber pine dwarf mistletoe), and *A. monticola* (western white pine dwarf mistletoe)—possess significantly different plant morphologies and host specificities when compared to each other and directly to *A. campylopodum* (Table 1, Table 16 and Table 17) [75]. All WPDMs also have distinct, non-sympatric geographic distributions when compared to each other, and only populations of *A. cyanocarpum* and *A. monticola* are known to be sympatric with *A. campylopodum* (Table 17) [1]. Among the WPDMs, limber pine dwarf has the largest geographic distribution (Table 1) and is the most morphologically distinct (Table 14). *Arceuthobium cyanocarpum* is found in dispersed and often high-elevation populations in central Oregon east to central Idaho and southern Montana, south to Colorado, and west throughout Utah, Nevada, and eastern California. Limber pine dwarf mistletoe is the least host-specialized of the WPDMs in so far as it has multiple white pine species as principal hosts (e.g., limber pine, whitebark pine, and two species of bristlecone pine) and its secondary (western white pine—*Pinus monticola* Douglas ex D. Don) and occasional hosts (sugar pine and foxtail pine—*P. balfouriana* subsp. *balfouriana*) are also white pines—i.e., in *Pinus*, subgenus *Strobus*. It will also rarely crossover to lodgepole pine and Rocky Mountain ponderosa pine in Colorado and mountain hemlock in Oregon [1]. There is, however, some uncertainty regarding the susceptibility classification of sugar pine as an occasional host [1]. Mathiasen and Daugherty [156] reported *A. cyanocarpum* severely infecting sugar pine near Tahquitz Peak in the San Jacinto Mountains, California; yet, unfortunately, the limited number of sugar pine in the area precluded a formal reassessment of the susceptibility of sugar pine to *A. cyanocarpum*.

In addition to its dispersed but extensive geographic distribution, limber pine dwarf mistletoe is the most morphologically identifiable of the WPDMs, as it produces small green, yellow–green, reddish, or near purple plants and has noticeably shortened third internode dimensions, as well as smaller 4-merous staminate flowers, anther diameters, fruits, and seeds in comparison to the other four WPDMS (Table 16). *Arceuthobium cyanocarpum* is most morphologically, and genetically similar to Apache dwarf mistletoe [69]; however, the latter dwarf mistletoe—*A. apachecum*—is a strict parasite of southwestern white pine (*Pinus strobiformis* Engelm.) in southern Arizona and New Mexico with a remote population in the Sierra del Carmen, Chihuahua, Mexico (Table 1, Table 16 and Table 17). *Arceuthobium apachecum* produces yellow–green, green, or reddish plants that are larger in height than *A. cyanocarpum*, but smaller in comparison to *A. blumeri*, *A. californicum*, and *A. monticola*. Apache dwarf mistletoe can also be differentiated from the other WPDMs by its third internodal length and staminate flower diameter, as well as the length of its petals, fruits, and seeds (Table 16). Like all but one WPDM (i.e., *A. californicum*), *A. apachecum* begins anthesis in July and concludes staminate flowering in September. Thus, staminate flowering period cannot be utilized alone to delineate *A. apachecum* from *A. blumeri* (Blumer’s dwarf mistletoe) as *A. blumeri* parasitizes southwestern white pine in the Huachuca Mountains, Arizona and south through the Sierra Madre Occidental into southern Durango, Mexico with outlying populations in Coahuila and Nuevo Leon, Mexico (Table 1) [41]. Blumer’s dwarf mistletoe is, however, gray, straw, or light green in color and the most “*A. campylopodum*-like” WPDM, producing the most robust plants of the group (particularly male plants and flowers). *Arceuthobium blumeri* attains the greatest height of all WPDMs, has consistently thicker third internodes, and produces the longest staminate spikes and the largest male flowers of the group (3- and 4-merous; Table 16).

**Table 16:** Section *Campylopoda*: white pine dwarf mistletoes. Principal morphological and physiological characters differentiating *Arceuthobium apachecum*, *A. blumeri*, *A. californicum*, *A. cyanocarpum*, and *A. monticola*. Morphological measurements are means followed by ranges [min., max.]; plant heights are in cm, and all other measurements are in mm. These data were published by Kenaley et al. [75].

Character	*Arceuthobium* *apachecum*	*Arceuthobium blumeri*	*Arceuthobium* *californicum*	*Arceuthobium* *cyanocarpum*	*Arceuthobium* *monticola*
**Plant color**	Yellow–green, green, reddish; males often yellow	Gray, straw, or light green	Green, yellow–green, or yellow; old plants often brown at base	Green, yellow–green, reddish, or near purple	Dark brown to reddish brown, yellow–green
**Plant height**					
Female	5.1 [2.6. 10.4]	10.0 [2.7, 18.4]	9.9 [5.8, 14.9]	3.6 [1.7, 6.7]	8.5 [6.1, 13.4]
Male	3.7 [1.2, 10.6]	9.8 [2.5, 18.4]	8.1 [3.8, 14.4]	2.8 [0.8, 6.3]	7.8 [4.4, 12.6]
**Basal diameter**					
Female	2.1 [1.4, 4.1]	2.8 [1.6, 4.6]	3.0 [1.9, 3.8]	2.0 [1.0, 9.5]	2.9 [2.1, 3.8]
Male	1.9 [1.1, 3.2]	2.7 [1.9, 4.1]	2.7 [1.8, 4.0]	1.8 [1.0, 3.1]	2.8 [2.0, 3.5]
**Third internode length**					
Female	8.2 [2.5, 17.4]	11.3 [3.9, 22.1]	11.5 [7.6, 20.1]	6.5 [2.3, 11.8]	11.5 [6.9, 23.0]
Male	5.9 [1.9, 14.0]	10.9 [4.6, 21.6]	10.1 [4.4, 17.9]	5.2 [1.8, 10.3]	9.9 [4.8, 17.0]
**Third internode width**					
Female	1.6 [1.1, 2.4]	2.0 [1.4, 2.7]	1.9 [1.5, 2.5]	1.5 [1.1, 2.3]	1.7 [1.3, 2.4]
Male	1.6 [1.0, 2.2]	2.1 [1.6, 2.8]	1.7 [1.3, 2.4]	1.5 [0.9, 2.2]	1.7 [1.3, 2.4]
**Staminate flowers**					
Anthesis (peak)	Late July–mid-Sept. (mid-Aug.)	Late July–late Aug. (early Aug.)	Mid-June–late July (late June–early July)	Early July–mid-Sept. (mid-July–early Sept.)	Late July–early Sept. (early to mid-Aug.)
Spike length	9.3 [4.5, 19.3]	13.7 [5.5, 27.8]	8.7 [4.1, 14.8]	5.8 [1.6, 11.7]	8.6 [5.0, 14.2]
Spike width	2.6 [1.4, 4.0]	2.6 [1.7, 4.1]	1.8 [1.1, 2.1]	2.5 [1.1, 3.9]	1.4 [1.1, 1.8]
Diameter 3-merous	2.7 [1.9, 3.7]	3.0 [2.4, 4.0]	2.6 [1.9, 3.4]	2.6 [1.9, 3.5]	2.5 [2.0, 3.1]
Diameter 4-merous	3.1 [2.0, 4.4]	4.1 [2.6, 5.3]	3.5 [2.5, 4.2]	2.8 [1.8, 3.8]	3.6 [2.8, 4.6]
**Fruit glaucous**	Lightly glaucous	Lightly glaucous	Lightly glaucous	Moderate to highly glaucous	Highly glaucous
**Fruit length**	4.1 [3.0, 5.7]	4.7 [1.4, 5.9]	5.1 [4.3, 6.0]	3.5 [2.5, 4.5]	4.7 [4.0, 5.6]
**Fruit width**	2.9 [2.0, 4.0]	2.8 [1.9, 3.4]	3.1 [2.5, 3.8]	2.4 [1.7, 3.2]	3.0 [2.4, 3.5]
**Seed length**	2.1 [1.3, 2.9]	2.2 [1.9, 2.8]	2.7 [2.0, 4.3]	1.9 [1.3, 2.5]	2.5 [1.8, 3.2]
**Seed dispersal (peak)**	Mid-Aug.–Oct. (mid-Sept.)	Mid-Aug.–early Oct. (mid-Sept.)	Mid-Sept.–mid-Oct., rarely early Nov. (early Oct.)	Mid-Aug.–late Sept. (early Sept.)	Late Aug.–early Oct. (early to mid-Sept.)

*Arceuthobium californicum* is geographically restricted to California, USA, from the vicinity of Dillon Mountain into the North Coast Range and the southern portion of the Cascades through the Sierra Nevada Mountains into San Diego County, wherein it exclusively parasitizes sugar pine and does not crossover to hard pines or other white pines across its geographic range (Table 1 and Table 17). Morphologically, sugar pine dwarf mistletoe is most similar to *A. monticola* (Table 16); however, *A. californicum* has more yellow or yellow–green shoots, it flowers in mid-June to late July, and its northern geographic limit is south of the southernmost limit of *A. monticola* in northern California. In contrast, western white pine dwarf mistletoe produces dark brown to red–brown or, yellow–green plants, flowers in late July to early September, and it has a limited geographic distribution. *Arceuthobium monticola* is only known to occur in the Klamath and Siskiyou Mountains in the northwestern border region of California and Oregon (Table 1), and its principal host is western white pine (Table 17). Hosts of *A. monticola* also include Brewer spruce (*Picea breweriana*) and sugar pine, yet these conifers are secondary and occasional hosts, respectively.

**Table 17:** Section *Campylopoda*: white pine dwarf mistletoes. Host susceptibility and sympatry among *Arceuthobium apachecum*, *A. blumeri*, *A. californicum*, *A. cyanocarpum*, and *A. monticola*. Across pairwise-species comparisons, the white pine dwarf mistletoes do not co-occur throughout their collective geographic distributions, and, hence, these species are non-sympatric [1]. **a**—host susceptibility classification based on information by Hawksworth and Wiens [1].

Character	*Arceuthobium* *apachecum*	*Arceuthobium* *blumeri*	*Arceuthobium* *californicum*	*Arceuthobium* *cyanocarpum*	*Arceuthobium* *monticola*
**Host susceptibility** ^a^					
Principal	*Pinus strobiformis*	*Pinus strobiformis*	*Pinus lambertiana*	*Pinus albicaulis*; *P. aristata*; *P. flexilis*; *P. longaeva*	*Pinus monticola*
Secondary				*Pinus monticola*	*Picea breweriana*
Occasional				*Pinus balfouriana* subsp. *balfouriana*; *P. lambertiana*?	*Pinus lambertiana*
Rare				*Pinus contorta* var. *latifolia*; *P. ponderosa* var. *scopulorum*; *Tsuga mertensiana*	
**Sympatry**	None	None	None	None	None

#### 5.7.3. Hemlock and Larch Dwarf Mistletoes

The hemlock- and larch-infecting dwarf mistletoes of section *Campylopoda* consists of two species (*Arceuthobium tsugense* and *A. laricis*) and four subspecies (*A. tsugense* subsp. *amabilae*, subsp. *contortae*, subsp. *mertensianae*, and subsp. *tsugense*). From the perspective of host-species diversity, *A. laricis* (larch dwarf mistletoe) and *A. tsugense* (hemlock dwarf mistletoe) collectively parasitize the most diverse collection of conifers in N. America with principal to rare hosts in five genera within Pinaceae (*Abies*, *Picea*, *Pinus*, *Tsuga*, and *Pseudotsuga*; Table 18 and Table 19). However, what unifies both species is their parasitism of mountain hemlock (*T*. *mertensiana*). Larch dwarf mistletoe is a principal parasite of western larch as well as a secondary parasite of mountain hemlock and lodgepole pine from central Oregon to west-central Idaho and north through central and eastern WA, northern Idaho, and western Montana, USA, and into southern British Columbia, Canada (Table 1) [157]. It will also occasionally occur on subalpine fir, Rocky Mountain alpine fir, (*Abies bifolia* A. Murray bis), and ponderosa pine (Table 18). Given its secondary parasitism of mountain hemlock, larch dwarf mistletoe can be confused with mountain hemlock dwarf mistletoe (*A. tsugense* subsp. *mertensianae*), even though this mistletoe is not known to infect western larch. However, comparing larch and mountain hemlock dwarf mistletoes reveals that *A. laricis* is morphologically different, and its geographic range, as well as its secondary and occasional hosts, do not overlap with those of *A. tsugense* subsp. *mertensianae* (Table 18). Larch dwarf mistletoe has green–brown, red to purple shoots with shorter and wider third internodes, and smaller 3-merous staminate flowers when compared to *A. tsugense* subsp. *mertensianae*. Moreover, although staminate flowering periods are similar, peak seed dispersal for *A. laricis* (September) is in advance of *A. tsugense* subsp. *mertensianae* (mid-October).

In addition to mountain hemlock dwarf mistletoe, *A. tsugense* is also divided into three additional subspecies: *A. tsugense* subsp. *amabilae* (Pacific silver fir dwarf mistletoe), subsp. *contortae* (shore pine dwarf mistletoe), and subsp. *tsugense* (western hemlock dwarf mistletoe). The four subspecies are distinguished according to several morphological characters, host affinities, and minor differences in flowering and/or seed dispersal periods (Table 19 and Table 20) [43,91,92,159]. For plant characteristics, shoot color alone is not an effective characteristic to separate subspecies of *A. tsugense*, as western hemlock dwarf mistletoe (yellow–green, purple) is the lone subspecies that is not green to green–brown in plant color. Yet, across taxa, the basal diameter and third internode length of male plants, staminate spike and petal length, anther distance to tip, and seed length differ among subspecies of *A. tsugense* (Table 19). Shore pine dwarf mistletoe is the most morphologically and physiologically distinct among the hemlock dwarf mistletoes, exhibiting significantly greater staminate flower and spike dimensions (e.g., flower diameter, petal length and width, anther distance to tip, and spike length and width), as well as having different seed measurements when compared to the other hemlock dwarf mistletoes. Moreover, subsp. *contortae* is highly host-specific (Table 20); having one principal host in shore pine—a hard pine—and an occasional host in western hemlock in northwestern Washington, USA, and southwestern British Columbia, Canada, where it co-occurs with subsp. *tsugense*. In sympatric populations with subsp. *tsugense*, shore pine dwarf mistletoe maintains its morphological integrity, host affinities, and disperses seed in advance of western hemlock dwarf mistletoe [43,92,159]. Thus, shore pine dwarf mistletoe may warrant recognition at the specific rank if future molecular evidence can provide support for its separation from *A. tsugense* [43].

Differences in host and geographic distribution also exist among *A. tsugense* subsp. *amabilae*, subsp. *mertensianae*, and subsp. *tsugense* (Table 1 and Table 20). In addition to mountain hemlock, subsp. *amabilae* principally parasitizes Pacific silver fir and noble fir in central Oregon, USA, where it will also infect subalpine fir as a secondary host and western hemlock as an occasional host. Pacific silver fir dwarf mistletoe is sympatric with mountain hemlock dwarf mistletoe in central Oregon; however, its larger and thicker shoots, staminate inflorescences and flowers, and fruit dimensions readily differentiate subsp. *amabilae* from subsp, *mertensiana* (Table 19). Mountain hemlock dwarf mistletoe also does not cross-infect Pacific silver fir (Table 20) [1,85,91,125]. The geographic distribution of subsp. *mertensianae* continues south from central Oregon through the Sierra Nevada to Mosquito Lake, California. Mountain hemlock dwarf mistletoe is morphologically and physiologically different (e.g., plant color, shoot and staminate spike dimensions, male flowers, and principal host) when compared to western hemlock dwarf mistletoe (Table 19). Peak anthesis for subsp. *mertensianae* (early September) also typically occurs one week later than subsp. *tsugense* (mid-August). Conversely, seed dispersal for mountain hemlock dwarf mistletoes typically peaks in mid-September, approximately two weeks earlier than western hemlock dwarf mistletoe. Subspecies *mertensianae* will also occasionally, but severely, infect Brewer spruce and western white pine; whereas subsp. *tsugense* severely parasitizes western hemlock as a principal host, while mountain hemlock as well as Pacific silver fir, noble fir, and shore pine have been consistently characterized as occasional hosts for western hemlock dwarf mistletoe. Given the discussed sympatry with subsp. *contortae* and subsp. *mertensianae*, the geographic distribution of subsp. *tsugense* is indeed the most expansive of the hemlock dwarf mistletoes (Table 1), beginning with isolated populations in the northern coastal ranges of northwestern CA extending—and becoming common—in Oregon and Washington, and in the coastal western hemlock forests of British Columbia and southern Alaska.

**Table 20:** Section *Campylopoda*: hemlock dwarf mistletoes. Host susceptibility and sympatry among *Arceuthobium tsugense* subsp. *amabilae*, subsp. *contortae*, subsp. *mertensianae*, and subsp. *tsugense*. Subspecies determined sympatric co-occur within two kilometers of each other [1]. **a**—host susceptibility classification system [1]. Host classifications for subsp. *amabilae* are based on field observations [43]. Host classifications for subsp. *tsugense* are based on Mathiasen and Daugherty [124] and Shaw [160]. Host classifications for subsp. *mertensianae* are based on the works of Mathiasen and Hawksworth [158] and Mathiasen and Kenaley [43].

Character	*Arceuthobium tsugense* subsp.			
	*amabilae*	*contortae*	*mertensianae*	*tsugense*
**Host susceptibility ^a^**				
Principal	*Abies amabilis*; *A. procera*; *Tsuga mertensiana*	*Pinus contorta* var. *contorta*	*Tsuga mertensiana*	*Tsuga heterophylla*
Secondary	*Abies lasiocarp*		*Picea breweriana*	
Occasional	*Tsuga heterophylla*	*Tsuga heterophylla*	*Pinus monticola*; *Tsuga heterophylla*	*Abies amabilis*; *A. procera*; *Pinus contorta* var. *contorta*; *Tsuga mertensiana*
Rare	*Abies grandis*; *Pinus monticola*	*Pinus monticola*		*Abies grandis*; *Picea engelmannii*; *P. sitchensis*; *Pinus monticola*; *Pseudotsuga menziesii*
**Sympatry**	subsp. *mertensianae*	subsp. *tsugense*	subsp. *tsugense*	subsp. *contortae*; subsp. *mertensianae*

#### 5.7.4. The Fir Dwarf Mistletoes

Excluding Pacific silver fir dwarf mistletoe, all taxa in section *Campylopoda* with true firs (*Abies* spp.) as a principal host (or principal hosts) are classified as *Arceuthobium abietinum* (fir dwarf mistletoe) and one of five subspecies based on several differences in host susceptibility, geographic distribution, and morphological characters (Table 21) [1,44,61,71]. Fir dwarf mistletoe occurs from southern Washington throughout Oregon and California and into southern Nevada, southern Utah, and Arizona, USA, with isolated populations extending into northern Mexico (Table 1). However, the overall geographic distribution and host affinities of *A. abietinum* are segregated by its five subspecies. From north to south, *A. abietinum* subsp. *grandae* (grand fir dwarf mistletoe) is found principally parasitizing grand fir and hybrid populations of grand fir x concolor fir in southern Washington through the Cascade and Siskiyou mountains in Oregon to the Klamath and southern Cascade Mountains of northern California [44], where it overlaps with *A. abietinum* subsp. *magnificae* (red fir dwarf mistletoe) (Table 1 and Table 21). Populations of grand fir dwarf mistletoe also occur in northeastern California (Warner Mountains) and northwestern California (Northern Coast Ranges). Although red fir dwarf mistletoe is found in sympatry with subsp. *grandae* near Mt. Shasta, California, subsp. *magnificae* specializes in red fir and its geographic distribution extends from northern California through the Sierra Nevada and terminates in the Greenhorn Mountains, California. Much of the geographic range of *A. abietinum* subsp. *abietinum* (white fir dwarf mistletoe) is consistent with the geographic range of subsp. *magnificae* from the vicinity of Mt. Lassen through the Sierra Nevada; however, it extends as far south as the San Bernardino Mountains of the Transverse Ranges in southern California. Subspecies *abietinum* also severely parasitizes Sierra white fir (*Abies lowiana* (Gordon) A. Murray bis), occasionally infects subalpine fir, and does not infect red fir (Table 21). Unlike the latter fir dwarf mistletoes, *A. abietinum* subsp. *wiensii* (Wien’s dwarf mistletoe) and subsp. *mathiasenii* (Mathiasen’s dwarf mistletoe) are not sympatric with each other nor with the other three subspecies of fir dwarf mistletoe. Wien’s dwarf mistletoe is one of the rarest dwarf mistletoes in N. America and parasitizes red fir and Brewer spruce as principal hosts. The subspecies *wiensii* occurs in isolated populations in northwestern California and southwestern Oregon from the vicinity of South Fork Mountain north through the Siskiyou and Klamath Mountains to Flat Top Mountain. Grand fir x concolor fir hybrids are also an occasional host of subsp. *wiensii* within its restricted range. The geographic distribution of Mathiasen’s dwarf mistletoe also consists of isolated populations; however, unlike subsp. *wiensii*, populations of subsp. *mathiasenii* occur principally on Rocky Mountain white fir in southern Utah, southwestern Nevada, and northern (Grand Canyon) and southern Arizona (Santa Catalina and Chiricahua Mountains), USA (Table 1 and Table 21). The subspecies *mathiasenii* also is found principally parasitizing Durango fir (*Abies durangensis* Mart.) in remote populations in the Sierra Madre Occidental in Chihuahua and Durango, Mexico, where it can cross over to its occasional host, Mexican spruce (*Picea mexicana* Mart.) [44].

Given the observed patterns of sympatry, grand fir dwarf mistletoe can be differentiated from red fir dwarf mistletoe by shoot color (e.g., yellow–green, green–brown vs. yellow–red–brown, red, respectively), shorter plants, smaller basal diameter and third internode dimensions, and longer staminate spikes in addition to its exclusive parasitism of grand fir and associated hybrids (Table 21). Red fir and white fir dwarf mistletoes are very similar; however, the latter mistletoe does not cross over to red fir and its period of seed dispersal begins approximately two weeks earlier than red fir dwarf mistletoe—even when these subspecies co-occur. Morphologically, subsp. *abietinum* also differs from subsp. *magnificae* with its larger, yellow to yellow–green plants, wider third internode, greater staminate spike measurements, and larger 3-merous flowers (Table 21). In addition to being rare, Wien’s dwarf mistletoe is the only fir dwarf mistletoe that principally parasitizes red fir and Brewer spruce. It also possesses morphologies that separate it from subsp. *magnificae*, which also infects red fir but not spruce. The subspecies *wiensii* has brown–green, red–brown to red shoots and produces plants and plant parts that are smaller in size when compared to subsp. *magnificae*. Mathiasen’s dwarf mistletoe is most similar to subsp. *wiensii* insofar as plant color and anatomical dimensions. Plant color is highly variable in subsp. *mathiasenii* across its geographic range; however, the shoots of subsp. *mathiasenii* are a distinctive bluish green, reddish brown to near red color. Pistillate and staminate plants of subsp. *mathiasenii* are similar in size to subsp. *wiensii*; however, female and male plants of subsp. *mathiasenii* are smaller than those of subsp. *abietinum*, subsp. *grandae*, and subsp. *magnificae* (Table 21). The subspecies *mathiasenii* also has larger staminate flowers (3-merous and 4-merous) when compared to the other subspecies of *A. abietinum.*

**Table 21:** Section *Campylopoda*: fir dwarf mistletoes. Principal morphological and physiological characters differentiating *Arceuthobium abietinum* subsp. *abietinum*, subsp. *grandae*, subsp. *magnificae*, subsp. *mathiasenii*, and subsp. *wiensii*. Subspecies were determined sympatric if such taxa co-occur within two kilometers of each other [1]. Morphological measurements are means followed by ranges [min., max.]; plant heights are in cm, and all other measurements are in mm. **a**—host susceptibility classification is based on information by Hawksworth and Wiens [1] and Mathiasen and Daugherty [71]. **b**—data were published originally by Mathiasen and Kenaley [61]. **c**—data published by Kenaley [44]. **d**—data published by Mathiasen and Daugherty [71] and Mathiasen and Kenaley [61].

Character	*Arceuthobium abietinum* subsp.				
	*abietinum * ^b^	*grandae * ^c^	*magnificae * ^b^	*mathiasenii * ^c^	*wiensii * ^d^
**Plant color**	Yellow, yellow–green	Yellow, yellow–green, green–brown	Yellow, yellow–red–brown, red	Blue–green, yellow–brown, brown, red	Brown–green, red–brown, red
**Plant height**					
Female	13.2 [7.4, 24.5]	11.5 [6.3, 18.6]	12.2 [6.8, 25.1]	9.5 [3.1, 17.2]	9.5 [3.8, 16.1]
Male	12.7 [5.2, 20.5]	11.3 [5.6, 20.8]	11.9 [6.2, 19.7]	9.3 [3.2, 16.5]	8.9 [3.5, 15.7]
**Third internode width**					
Female	2.4 [1.4, 3.6]	2.0 [1.4, 3.3]	2.2 [1.6, 4.1]	2.2 [1.5, 3.3]	1.9 [1.3, 2.9]
Male	2.4 [1.3, 3.7]	2.0 [1.3, 3.3]	2.2 [1.6, 3.2]	2.2 [1.5, 3.1]	1.9 [1.4, 2.9]
**Staminate flowers**					
Spike length	10.4 [5.1, 16.5]	9.9 [4.6, 19.8]	9.4 [3.9, 19.2]	11.3 [4.9, 26.3]	8.7 [3.5, 17.0]
Spike width	2.1 [1.4, 2.8]	2.0 [1.3, 2.8]	2.0 [1.3, 3.1]	2.4 [1.6, 3.1]	1.5 [1.2, 2.0]
Diameter 3-merous	2.8 [2.1, 3.5]	2.7 [2.1, 3.7]	2.6 [2.0, 3.7]	3.1 [2.0, 3.9]	2.4 [2.0, 2.9]
Diameter 4-merous	3.7 [2.7, 4.8]	3.7 [2.7, 4.8]	3.8 [2.6, 5.0]	4.1 [2.8, 5.2]	3.2 [2.6, 3.8]
Petal length	1.4 [0.9, 2.0]	1.4 [1.0, 2.0]	1.4 [0.8, 2.0]	1.5 [1.0, 2.0]	1.2 [0.8, 1.9]
Petal width	1.2 [0.8, 1.6]	1.2 [0.8, 1.6]	1.2 [0.7, 1.7]	1.4 [0.7, 2.0]	1.0 [0.8, 1.4]
**Fruit length**	4.9 [3.5, 6.1]	4.7 [1.3, 6.2]	4.7 [3.4, 5.9]	4.7 [3.7, 6.0]	4.2 [3.1, 5.0]
**Seed dispersal (peak)**	Early Sept.–early Oct. (mid-Sept.)	Late Aug.–early Oct. (mid-Sept.)	Mid-Sept.–late Oct. (late Sept.)	Late Aug.–late Sept. (early Sept.)	Early Sept.–mid-Oct. (late Sept.–early Oct.)
**Host susceptibility ^a^**					
Principal	*Abies lowiana*	*Abies grandis*; *A. grandis* × *A. concolor*	*Abies magnifica*	*Abies concolor*; *A. durangensis*	*Abies magnifica; Picea breweriana*
Secondary					
Occasional	*Abies lasiocarpa*			*Picea mexicana*	*Abies grandis × concolor*
Rare	*Abies amabilis*; *Pinus contorta* var. *murrayana*; *P. lambertiana*; *P. monticola*	*Picea engelmannii*; *Pinus lambertiana*		*Pinus strobiformis*	*Pinus monticola*
**Sympatry**	subsp. *magnificae*	subsp. *magnificae*	subsp. *grandae*	None	None

#### 5.7.5. Western Spruce Dwarf Mistletoes

Western spruce dwarf mistletoe (*Arceuthobium microcarpum*) consists of two subspecies (subsp. *microcarpum* and subsp. *aristatae*) that are endemic to Arizona and New Mexico and have host distributions and several minor morphological differences that readily differentiate them (Table 22). *Arceuthobium microcarpum* subsp. *microcarpum* (western spruce dwarf mistletoe) primarily parasitizes blue spruce and Engelmann spruce and is known to occur in the North Rim Grand Canyon, White Mountains, and Pinaleño Mountains of Arizona and in the Mogollon Mountains and Sacramento Mountains of New Mexico. Western spruce dwarf mistletoe also parasitizes corkbark fir (*Abies lasiocarpa* (Hook) Nutt. var. *arizonica* (Merriam) Lemmon) as a rare host. In contrast, *A. microcarpum* subsp. *aristatae* (bristlecone pine dwarf mistletoe) occurs in very restricted populations on Kendrick Peak and in the San Francisco Peaks near Flagstaff, Arizona, where it parasitizes Rocky Mountain bristlecone pine as a principal host, Engelmann spruce as an occasional host, and southwestern white pine as a rare host (Table 22) [37]. Both subspecies are sympatric in the San Francisco Peaks; however, they maintain their host preferences, and they possess morphological and minor phenological differences. Bristlecone pine dwarf mistletoe produces light green to purple plants, whereas the shoots of western spruce dwarf mistletoes are light green to green–brown or blue-green. Moreover, plants of subsp. *aristatae* are smaller in size, and this difference in plant height is maintained when these dwarf mistletoes are compared on a shared host in Engelmann spruce. Anthesis for subsp. *aristatae* in the high peaks around Flagstaff occurs one to two weeks earlier than subsp. *microcarpum* in the White Mountains [37].

## 6. Conclusions

The last comprehensive review of *Arceuthobium* in N. America was published by Hawksworth and Wiens [1], and since then, nearly three decades of taxonomic and phylogenetic studies have significantly improved our understanding of these ecologically and economically important forest parasites. However, continued studies into the taxonomy and molecular phylogenetics of N. *Arceuthobium* are important due to the inherent taxonomic challenges of the genus, historically inconsistent sampling of plant characters, and knowledge gaps that have fueled the debate on the recognition of species and subspecies in the genus. To that end, across the preceding sections, we have provided a thorough review of the (1) taxonomic history of N. American *Arceuthobium*, (2) the molecular, morphological, and physiological evidence supporting the infrageneric classification of taxa amassed since 1996, and (3) the key taxonomic characteristics (e.g., plant characters/traits, host affinities, and geographic distribution) for all 44 taxa of *Arceuthobium* presently recognized to constitute the genus in N. America.

## Figures and Tables

**Table 2:** Classification systems of North American *Arceuthobium*: Gill [14] and Hawksworth and Wiens [17]. **A**. Gill [14] recognized five (5) species in the United States and divided *A. campylopodum* into eight (8) forms according to host. **B**. Hawksworth and Wiens [17] recognized 25 species in N. America and designated two subgenera, three sections, and three series.

**A**. Gill [14]—United States 1. *A. americanum* Nutt. ex Engelm. 2. *A. campylopodum* Engelm. 2a. *A. campylopodum* f. *typicum* Gill [including *A. occidentale* Engelm.] 2b. *A. campylopodum* f. *divaricatum* (Engelm.) Gill 2c. *A. campylopodum* f. *abietinum* (Engelm.) Gill 2d. *A. campylopodum* f. *tsugensis* (Rosendahl) Gill 2e. *A. campylopodum* f. *laricis* (Piper) Gill 2f. *A. campylopodum* f. *cyanocarpum* (A. Nelson) Gill 2g. *A. campylopodum* f. *blumeri* (A. Nelson) Gill 2h. *A. campylopodum* f. *microcarpum* (Engelm.) Gill 3. *A. douglasii* Engelm. 4. *A. pusillum* Peck 5. *A. vaginatum* (Willd.) Presl 5a. *A. vaginatum* f. *cryptopodum* (Engelm.) Gill	**B**. Hawksworth and Wiens [17], excluding Old World taxa. **Subgenus *Arceuthobium*** New World species 1. *A. abietis-religiosae* Heil 2. *A. americanum Nutt.* ex Engelm. 3. *A. verticilliflorum* Engelm. **Subgenus *Vaginata* Hawksw. & Wiens** Section *Vaginata* 4. *A. gillii* Hawksw. & Wiens 4a. *A. gillii* subsp. *gillii* 4b. *A. gillii* subsp. *nigrum* Hawksw. & Wiens 5. *A. globosum* Hawksw. & Wiens 6. *A. vaginatum* (Willd.) Presl 6a. *A. vaginatum* subsp. vaginatum 6b. *A. vaginatum* (Willd.) Presl subsp. cryptopodum (Engelm.) Hawksw. & Wiens 6c. *A. vaginatum* (Willd.) Presl subsp. durangense Hawksw & Wiens Section *Campylopoda* Hawksw. & Wiens Series *Campylopoda* 7. *A. abietinum* Engelm. ex Munz 7a. *A. abietinum* f. sp. *concoloris* Hawksw. & Wiens 7b. *A. abietinum* f. sp. *magnificae* Hawksw. & Wiens 8. *A. apachecum* Hawksw. & Wiens 9. *A. blumeri* A. Nelson 10. *A. californicum* Hawksw. & Wiens 11. *A. campylopodum* Engelm. 12. *A. cyanocarpum* Coulter & Nelson 13. *A. divaricatum* Engelm. 14. *A. guatemalense* Hawksw. & Wiens 15. *A. laricis* (Piper) St. John 16. *A. littorum* Hawksw., Wiens & Nickrent 17. *A. microcarpum* (Engelm.) Hawksw. & Wiens 18. *A. occidentale* Engelm. 19. *A. tsugense* (Rosendahl) G.N. Jones Series *Rubra* Hawksw. & Wiens 20. *A. bicarinatum* Urban 21. *A. hondurense* Hawksw. & Wiens 22. *A. rubrum* Hawksw. & Wiens Series *Stricta* Hawksw. & Wiens 23. *A. strictum* Hawksw. & Wiens Section *Minuta* Hawksw. & Wiens 24. *A. douglasii* Engelm. 25. *A. pusillum* Peck.

**Table 4:** Modified and updated classification system of North American *Arceuthobium* based on Hawksworth and Wiens [1], reflecting molecular, morphological, phenological, and physiological differences among taxa.

**Subgenus *Vaginata* Hawksw. & Wiens** Section *Americana* Nickrent 1. *A. abietis-religiosae* Hiel 2. *A. americanum* Nutt. ex Engelm. 3. *A. verticilliflorum* Engelm. Section *Penda* Nickrent 4. *A. guatemalense* Hawksw. & Wiens 5. *A. pendens* Hawksw. & Wiens Section *Globosa* Nickrent 6. *A. globosum* Hawksw. & Wiens 6a. *A. globosum* subsp. *globosum* 6b. *A. globosum* subsp. *aureum* (Hawksw. & Wiens) Mathiasen 6c. *A. globosum* subsp. *grandicaule* Hawksw. & Wiens 6d. *A. globosum* subsp. *petersonii* Hawksw. &Wiens Section *Pusilla* Nickrent 7. *A. bicarinatum* Urban. 8. *A. pusillum* Peck Section *Rubra* Hawksw. & Wiens 9. *A. gillii* Hawksw. & Wiens 10. *A. nigrum* Hawksw. &Wiens 11. *A. rubrum* Hawksw. & Wiens [including *A. oaxacanum* Hawksw. & Wiens] 12. *A. yecorense* Hawksw. & Wiens Section *Vaginata* Hawksw. & Wiens 13. *A. hondurense* Hawksw. & Wiens 13a. *A. hondurense* subsp. *hondurense* 13b. *A. hondurense* subsp. *hawksworthii* (Wiens & C.G. Shawn bis) Mathiasen 14. *A. strictum* Hawksw. & Wiens 15. *A. vaginatum* (Willd.) Presl. 15a. *A. vaginatum* subsp. *vaginatum* 15b. *A. vaginatum* subsp. *cryptopodum* (Engelm.) Hawksw. & Wiens 15c. *A. vaginatum* subsp. *durangense* Hawksw. & Wiens Section *Minuta* Hawksw. & Wiens 16. *A. divaricatum* Engelm. 17. *A. douglasii* Engelm. Section *Campylopoda* Hawksw. & Wiens 18. *A. abietinum* (Engelm.) Engelm. ex Munz 18a. *A. abietinum* subsp. *abietinum* 18b. *A. abietinum* subsp. *grandae* Kenaley 18c. *A. abietinum* subsp. *magnificae* Mathiasen & Kenaley 18d. *A. abietinum* subsp. *mathiasenii* Kenaley 18e. *A. abietinum* subsp. *wiensii* Mathiasen & C. Daugherty 19. *A. apachecum* Hawksw. & Wiens 20. *A. blumeri* A. Nelson 21. *A. californicum* Hawksw. & Wiens 22. *A. campylopodum* Engelm. 23. *A. cyanocarpum* (A. Nelson ex Rydberg) Coulter & Nelson 24. *A. laricis* (Piper) St. John 25. *A. littorum* Hawksw., Wiens & Nickrent 26. *A. microcarpum* (Engelm.) Hawksworth & Wiens 26a. *A. microcarpum* subsp. *microcarpum* 26b. *A. microcarpum* subsp. *aristatae* J.M. Scott & Mathiasen 27. *A. monticola* Hawksw. Wiens & Nickrent 28. *A. occidentale* Engelm. 29. *A. siskiyouense* Hawksw., Wiens & Nickrent 30. *A. tsugense* (Rosendahl) G.N. Jones] 30a. *A. tsugense* subsp. *tsugense* 30b. *A. tsugense* subsp. *amabilae* Mathiasen & C. Daugherty 30c. *A. tsugense* subsp. *contortae* Wass & Mathiasen 30d. *A. tsugense* subsp. *mertensianae* Hawksw. & Nickrent

**Table 8:** Section *Globosa.* Principal morphological and physiological differences, geographic distributions, and sympatry among subspecies of *Arceuthobium globosum*. Morphological measurements are means followed by ranges [min., max.]; plant heights are in cm and all other measurements are in mm. A solitary question mark indicates that the character information remains unresolved. Morphological data were published in Mathiasen [53], whereas host affinities were published by Hawksworth and Wiens [1] as well as Mathiasen [53].

Character	*Arceuthobium globosum* subsp.			
	*aureum*	*globosum*	*grandicaule*	*petersonii*
**Plant color**	Yellow (golden)	Yellow	Yellow–green	Yellow–brown
**Plant height**				
Female	14.2 [8.2, 21.6]	19.6 [8.0, 44.7]	37.5 [17.0, 66.0]	27.2 [18.1, 46.2]
Male	15.4 [8.1, 31.4]	21.0 [11.3, 42.3]	44.7 [17.1, 93.3]	32.6 [19.6, 46.8]
**Basal diameter**				
Female	5.7 [2.8, 13.3]	12.7 [4.2, 25.6]	17.5 [6.0, 40.1]	10.4 [6.6, 20.1]
Male	5.5 [3.0, 13.4]	11.3 [4.5, 31.6]	16.1 [6.0, 37.1]	10.2 [4.2, 22.9.]
**Third internode length**				
Female	16.9 [10.8, 30.2]	19.9 [4.2, 35.0]	33.7 [15.0, 56.2]	23.1 [9.4, 34.1]
Male	18.0 [9.7, 29.3]	20.0 [9.7, 45.6]	32.8 [15.0, 54.2]	25.7 [13.8, 38.3]
**Third internode width**				
Female	3.5 [1.8, 7.3]	7.3 [3.0, 18.8]	11.6 [4.0, 25.5]	6.4 [4.3, 16.7]
Male	3.2 [1.9, 6.4]	6.2 [2.7, 19.9]	10.4 [4.0, 31.4]	5.6 [3.4, 13.7]
**Staminate flowers**				
Anthesis (peak)	Mid-Feb.–June (no apparent peak)	Mar.–April (Mar.)	Jan.–May (Mar.–Apr.)	Mid-Aug.–early Oct. (mid-Sept.).
Staminate spike width	1.2 [0.8, 1.2]	2.1 [1.8, 2.3]	2.2 [1.9, 2.4]	1.3 [0.8, 1.4]
Diameter 3-merous	2.2 [1.9, 2.6]	3.1 [2.5, 4.0]	3.1 [2.1, 4.0]	2.2 [1.9, 2.4]
Diameter 4-merous	2.9 [3.1, 4.4]	4.2 [3.1, 5.6]	4.2 [3.0, 5.8]	3.0 [2.6, 3.5]
**Fruit length**	4.1 [3.6, 4.6]	6.7 [5.3, 8.2]	7.0 [5.6, 8.2]	5.1 [4.5, 5.7]
**Fruit width**	2.7 [2.3, 3.0]	4.1 [2.9, 5.2]	4.1 [3.1, 5.2]	3.4 [2.9, 4.0]
**Seed dispersal (peak)**	Aug.–early Oct. (no apparent peak)	? (June–July)	Mid-Aug.–Sept. (?)	Mid-Aug.–early Oct. (mid-Sept.)
**Witches’ brooms**	Common	Rare	Common	Common
**Host(s)**				
Principal	*Pinus maximinoi*; *P. montezumae*; *P. oocarpa*?; *P. pseudostrobus* subsp. *oaxacana* (*P. oaxacana*)	*Pinus cooperi*; *P. durangensis*; *P. engelmannii*	*Pinus douglasiana*; *P. devoniana* (*P. michoacana*); *P. durangensis*; *P. hartwegii* (*P. rudis*); *P. lawsonii*; *P. maximinoi*; *P. montezumae*; *P. patula*; *P. pringlei*; *P. pseudostrobus*; *P. teocote*	*Pinus maximinoi; P. montezumae*; *P. oocarpa*; *P. patula (P. oocarpa* var. *ochoterenai)*?; *P. pseudostrobus* subsp. *oaxacana* (*P. oaxacana*)?
Secondary				*Pinus devoniana (P*. *michoacana*)
Occasional		*Pinus arizonica*	-	
Rare		*Pinus teocote*		
**Distribution**	Chiapas, MEX; GTM	Northwest MEX	Central and southern MEX; GTM; HND	Chiapas, MEX
**Sympatry**	subsp. *grandicaule* (Chiapas, MEX)	None	subsp. *aureum* (Chiapas, MEX)	?

**Table 9:** Section *Pusilla*. Principal morphological and physiological differences, geographic distributions, and sympatry between *Arceuthobium bicarinatum* and *A. pusillum*. Morphological measurements are means followed by ranges [min., max.]; plant heights are in cm, and all other measurements are in mm. Consecutive en dashes indicate that the statistic has not been reported, while a solitary question mark indicates the character information remains unresolved. These data were published by Hawksworth and Wiens [1].

Character	*Arceuthobium bicarinatum*	*Arceuthobium pusillum*
**Plant color**	Dark brownish red	Green, brown
**Plant height**	10. 0 [---, 17.0]	1.0 [ ---, 3.0]
**Basal diameter**	3.0 [2.0, 4.0]	1.0 [---, ---]
**Third internode length**	10.5 [6.0, 14.0]	1.9 [1.0, 4.0]
**Third internode width**	2.0 [1.5, 4.0]	1.0 [0.5, 1.5]
**Anthesis**	Sept.	Apr.–May
**Apical nodes sterile on pistillate plants**	Yes	No
**Fruit length**	4.0 [---, ---]	3.0 [---, ---]
**Fruit width**	2.0 [---, ---]	1.5 [1.3, 1.8]
**Seed length**	2.5 [---, ---]	2.0 [---, ---]
**Seed width**	1.2 [---, ---]	0.9 [---, ---]
**Seed dispersal**	Late Aug.–Sept.	Sept.–early Oct.
**Systemic infection**	?	Frequent
**Witches’ brooms**	Frequent	Frequent
**Host susceptibility**		
Principal	*Pinus occidentalis*	*Picea mariana*; *P. glauca*; *P. rubens*
Secondary		
Occasional		*Larix laricina*
Rare		*Abies balsamea*; *Pinus banksiana*; *P. resinosa*; *P. strobus*
**Distribution**	Hispaniola (DMN, HTI)	North Central–Northeastern USA and South-central—Eastern CAN, including maritime provinces
**Sympatric**	No	No

**Table 18:** Section *Campylopoda*: larch dwarf mistletoe. Morphological and physiological characteristics comparing larch dwarf mistletoe (*Arceuthobium laricis*) and mountain hemlock dwarf mistletoe (*A. tsugense* subsp. *mertensianae*). The comparison was chosen since both species are principal parasites of mountain hemlock (*Tsuga mertensiana*). Morphological measurements are means followed by ranges [min., max.]; plant heights are in cm, and all other measurements are in mm. **a**—data for *Arceuthobium laricis* were published by Mathiasen and Kenaley [42]. **b**—data for *Arceuthobium tsugense* subsp. *mertensianae* were published by Mathiasen and Kenaley [43]. **c**—host susceptibility classification based on information by Hawksworth and Wiens [1], Mathiasen [157], and Mathiasen and Hawksworth [158].

Character	*Arceuthobium laricis * ^a^	*Arceuthobium tsugense subsp. mertensianae * ^b^
**Plant color**	Yellow–green, brown–green, occasionally purple–red	Green, green–brown, yellow–green
**Plant height**		
Female	5.3 [3.1, 9.8]	6.1 [3.0, 11.6]
Male	4.7 [2.1, 8.6]	5.7 [2.7, 9.8]
**Basal diameter**		
Female	2.4 [1.4, 4.1]	2.2 [1.4, 3.7]
Male	2.1 [1.4, 5.6]	1.9 [1.0, 3.3]
**Third internode length**		
Female	8.5 [3.8, 15.6]	9.8 [4.0, 19.0]
Male	7.5 [2.6, 11.6]	8.0 [2.0, 16.0]
**Third internode width**		
Female	1.7 [1.2, 2.4]	1.4 [1.0, 2.4]
Male	1.7 [1.3, 2.5]	1.3 [0.7, 1.9]
**Staminate flower**		
Anthesis	Mid-July to mid-Aug.–late August to mid-Sept.	Mid-Aug.–late Sept.
Spike length	10.1 [5.5, 18.1]	6.9 [2.0, 14.0]
Spike width	2.6 [2.0, 3.1]	1.2 [0.7, 1.4]
Diameter 3-merous	2.7 [2.0, 3.4]	2.4 [1.9, 2.9]
Diameter 4-merous	3.7 [3.0, 4.8]	3.1 [2.4, 3.8]
Petal lobe length	1.4 [0.9, 1.8]	1.1 [0.7, 1.5]
Petal lobe width	1.2 [0.8, 1.6]	1.0 [0.7, 1.3]
Anther diameter	0.5 [0.3, 0.8]	0.4 [0.3, 0.7]
Anther distance from tip	0.5 [0.2, 0.8]	0.4 [0.2, 0.7]
**Fruit length**	4.3 [3.5, 5.5]	4.4 [3.3, 5.5]
**Fruit width**	3.0 [2.1, 3.7]	2.6 [1.8, 3.5]
**Seed length**	2.4 [1.7, 3.2]	2.6 [1.8, 3.5]
**Seed width**	1.2 [0.7, 1.3]	1.1 [0.8, 1.4]
**Seed dispersal (peak)**	Late Aug.–early Oct. (Sept.)	Late Sept.–early Nov. (mid-Oct.)
**Host susceptibility** ^c^		
Principal	*Larix occidentalis*;	*Tsuga mertensiana*
Secondary	*Tsuga mertensiana* *Pinus contorta* var. *latifolia*	*Picea breweriana*
Occasional	*Abies lasiocarpa*; *A. bifolia*; *Pinus ponderosa* var. *ponderosa*	*Pinus monticola*; *Tsuga heterophylla*
Rare	*Abies grandis*; *Picea engelmannii*; *Pinus albicaulis*; *P. monticola*	
**Sympatric**	None; the southernmost distribution of *A. laricis* extends into central Oregon whereas the northern distribution of *A. tsugense* subsp. *mertensianae* terminates in south-central Oregon.	

**Table 19:** Section *Campylopoda*: hemlock dwarf mistletoes. Principal morphological and physiological characters differentiating *A. tsugense* subsp. *amabilae*, subsp. *contortae*, subsp. *mertensianae*, and subsp. *tsugense*. Morphological measurements are means followed by ranges [min., max.]; plant heights are in cm, and all other measurements are in mm. These data were published originally in Mathiasen and Kenaley [43]. **a**—measurements of staminate flower diameter for subsp. *contortae* did not distinguish between 3- and 4-merous flowers; thus, to compare flower diameter across all taxa, 3- and 4-merous flowers were combined.

Character	*Arceuthobium tsugense* subsp.			
	*amabiliae*	*contortae*	*mertensianae*	*tsugense*
**Plant color**				
Female	Green, green–brown	Green–brown	Green, green–brown	Yellow–green, purple
Male	Green–brown, yellow–green, green	Green–brown	Yellow–green, green–brown	Yellow–green
**Plant height**				
Female	10.6 [4.6, 18.5]	6.6 [4.0, 9.5]	6.1 [3.0, 11.6]	8.0 [3.8, 13.7]
Male	9.4 [4.1, 17.9]	5.6 [3.2, 10.8]	5.7 [2.7, 9.8]	7.8 [3.4, 16.1]
**Basal diameter**				
Female	3.4 [1.3, 5.8]	3.3 [1.8, 5.0]	2.2 [1.4, 3.7]	2.7 [1.3, 5.5]
Male	3.1 [1.8, 4.7]	2.8 [1.7, 4.7]	1.9 [1.0, 3.3]	2.6 [1.3, 5.0]
**Third internode length**				
Female	15.0 [7.0, 28.0]	10.7 [5.3, 16.4]	9.8 [4.0, 19.0]	12.3 [6.0, 22.0]
Male	12.6 [6.0, 22.0]	9.2 [5.8, 15.7]	8.0 [2.0, 16.0]	11.8 [4.5, 23.0]
**Third internode width**				
Female	2.0 [1.0, 3.0]	1.7 [1.3, 2.5]	1.4 [1.0, 2.4]	1.6 [1.0, 3.1]
Male	1.9 [1.1, 3.0]	1.8 [1.1, 2.5]	1.3 [0.7, 1.9]	1.6 [0.8, 3.0]
**Staminate flowers**				
Anthesis	Mid-July–mid-Sept. (late July–mid-Aug.)	Mid-July–early Oct. (late July–mid-Aug.)	Mid-Aug.–late Sept. (early Sept.; 1 wk later than subsp. *tsugense*)	Late July–late Sept. (mid-Aug.; 1 wk earlier than subsp. *mertensianae*)
Spike length	9.5 [3.0, 22.0]	12.6 [5.0, 22.0]	6.9 [2.0, 14.0]	10.8 [5.0, 27.4]
Spike width	1.3 [1.0, 2.1]	3.4 [2.0, 5.5]	1.2 [0.7, 1.4]	1.6 [0.9, 5.0]
Diameter 3-merous	3.2 [2.3, 4.9]	---	2.4 [1.9, 2.9]	3.1 [2.2, 4.7]
Diameter 4-merous	3.7 [2.6, 5.1]	---	3.1 [2.4, 3.8]	3.8 [2.5, 5.5]
3- and 4-merous **^a^**	3.4 [2.3, 5.1]	4.3 [2.8, 5.9]	2.7 [1.9, 3.8]	3.5 [2.2, 5.5]
**Petal length**	1.4 [1.0, 1.9]	1.7 [1.2, 2.8]	1.1 [0.7, 1.5]	1.5 [1.0, 2.1]
**Petal width**	1.2 [0.8, 1.8]	1.4 [1.0, 2.3]	1.0 [0.7, 1.3]	1.2 [0.8, 2.1]
**Fruit length**	4.7 [3.2, 6.0]	4.6 [3.3, 5.5]	3.8 [2.8, 4.8]	4.4 [3.3, 5.5]
**Seed length**	3.0 [2.3, 3.8]	2.5 [1.8, 3.0]	2.8 [2.2, 3.4]	2.6 [1.8, 3.5]
**Seed width**	1.2 [0.8, 1.5]	1.4 [1.0. 1.7]	1.1 [0.8, 1.4]	1.1 [0.8, 1.4]
**Seed dispersal (peak)**	Early Sept.–late Oct. (late Sept.–early Oct.; 2 wks earlier than subsp. *tsugense*)	Mid-Sept.–early Nov. (mid-Sept.–mid-Oct.; 1 wk earlier than subsp. *tsugense*)	Mid-Aug.–early Oct. (mid-Sept.; 2 wks earlier than subsp. *tsugense*)	Late Sept.–early Nov. (mid-Oct.; 2 wks later subsp. *amabilae* and subsp. *mertensianae*)

**Table 22:** Section *Campylopoda*: western spruce dwarf mistletoes. Principal morphological and physiological characters differentiating bristlecone pine dwarf mistletoe (*Arceuthobium microcarpum* subsp. *aristatae*) and western spruce dwarf mistletoes (*A. microcarpum* subsp. *microcarpum*). Morphological measurements are means followed by ranges [min., max.]; plant heights are in cm, and all other measurements are in mm. These data were published by Scott and Mathiasen [37]. **a**—host susceptibility classification system follows that of Hawksworth and Wiens [1] with host determinations according to Mathiasen and Hawksworth [113], Hawksworth and Wiens [1], and Scott and Mathiasen [37].

Character	*Arceuthobium microcarpum* subsp. *aristatae*	*Arceuthobium microcarpum* subsp. *microcarpum*
**Plant color**	Light green, green–brown, purple	Light green, green–brown, blue-green
**Plant height**		
Female	3.6 [1.4, 7.0]	6.9 [2.0, 15.7]
Male	2.7 [0.8, 7.0]	6.0 [1.8, 14.9]
**Basal diameter**		
Female	1.8 [0.6, 3.0]	2.0 [0.8, 3.8]
Male	1.8 1.0, 3.0]	1.9 [0.8, 3.4]
**Anthesis (peak)**	Mid-July–early Sept. (mid-Sept.; 1-2 wks. earlier than subsp. *microcarpum*)	Mid-July–late Sept. (late Aug.–mid-Sept.; 1-2 wks. later than subsp. *aristatae*)
**Staminate flower diameter**	2.5 [1.8, 4.0]	2.4 [1.6, 3.1]
**Fruit length**	3.3 [3.5–5.1]	3.5 [3.4 [3.4–5.2]
**Fruit width**	2.1 [1.7–2.9]	2.2 [1.9–3.1]
**Seed length**	2.4 [1.5–3.4]	2.4 [1.3–3.4]
**Seed dispersal (peak)**	Sept. (Sept.)	Sept.–Oct. (Sept.)
**Host susceptibility ^a^**		
Principal	*Pinus aristata*	*Picea engelmannii*; *P. pungens*
Secondary	*Picea engelmannii*	
Occasional		
Rare	*Abies lasiocarpa* var. *arizonica*; *Pinus strobiformis*	*Abies lasiocarpa* var. *arizonica*; *Pinus strobiformis*
**Distribution**	Limited to high elevation; USA—AZ (Kendrick Peak and San Francisco Peaks)	Limited to high elevation; USA—AZ and NM
**Sympatry**	subsp. *microcarpum*; San Francisco Peaks, AZ, USA	subsp. *aristatae*; San Francisco Peaks, AZ, USA

## Data Availability

No new data were created or analyzed in this study.
